# Mapping the Digital Mind: A Meta-Analysis of EEG Biomarkers in Cognition, Emotion, and Mental Health

**DOI:** 10.3390/brainsci16040368

**Published:** 2026-03-29

**Authors:** Constantinos Halkiopoulos, Evgenia Gkintoni, Basilis Boutsinas

**Affiliations:** 1 Department of Management Science and Technology, University of Patras, 26334 Patras, Greece; 2 Department of Psychiatry, University General Hospital of Patras, 26504 Patras, Greece; evigintoni@upatras.gr; 3 Department of Business Administration, University of Patras, 26504 Patras, Greece; vutsinas@upatras.gr

**Keywords:** electroencephalography, EEG biomarkers, cognitive control, frontal-midline theta, emotion regulation, late positive potential, neurofeedback, PTSD, mental health, systematic review, meta-analysis

## Abstract

**Highlights:**

**What are the main findings?**

- Frontal-midline theta oscillations are the most robust EEG biomarker across cognitive domains, with large effect sizes for response inhibition (*k* = 12; *d* = 0.89, 95% CI [0.72, 1.07]) and learning/memory consolidation (*k* = 10; *d* = 0.70, 95% CI [0.50, 0.89]), demonstrating a domain-general role in adaptive behavior. These findings showed a substantial consistency across the studies reviewed (*I*^2^ = 0.0%); however, this low heterogeneity should be interpreted with caution given the methodological diversity across paradigms, populations, and EEG systems.
- The late positive potential (LPP) serves as a potentially sensitive neurophysiological indicator of emotional processing (*k* = 18; *d* = 0.87, 95% CI [0.75, 1.00]) and emotion regulation success via cognitive reappraisal (*k* = 14; *d* = −0.65, 95% CI [−0.79, −0.51]), supporting its candidacy as a potential treatment target and outcome measure in clinical interventions, subject to further validation.
- Neurofeedback interventions show promising effects with effect sizes in a range comparable to some established treatments, with preliminary evidence of very large effects for PTSD (*k* = 2; *d* = −1.98, 95% CI [−2.50, −1.47])—though based on only two studies and requiring independent replication—and moderate effects for anxiety (*d* = −0.62), ADHD (*d* = −0.60), and depression (*d* = −0.42).
- Near-zero heterogeneity (*I*^2^ = 0.0% for six of seven primary meta-analyses) indicates a substantial consistency across the studies reviewed, pending independent replication in prospective cohorts; this should be interpreted cautiously as it may reflect restrictive inclusion criteria or insufficient statistical power rather than true homogeneity.

**What are the implications of the main findings?**

- EEG-based biomarkers offer a cost-effective, non-invasive pathway for personalized mental health assessment, enabling the prediction of treatment response (e.g., gender-specific frontal alpha asymmetry for antidepressant outcomes), real-time monitoring of emotional states in clinical populations, and brain–computer interface applications for adaptive intervention support.
- A four-phase implementation framework can guide clinical translation, addressing (1) technical infrastructure standardization, (2) clinical validation pathways, (3) accessible technology development, and (4) ethical training and implementation — bridging the gap between research findings and routine clinical practice.
- The consistency of EEG biomarker effects across the studies reviewed (*I*^2^ = 0.0% for six of seven analyses) is encouraging; however, independent replication in diverse, prospective cohorts is required before these findings can inform clinical practice, highlighting the potential of EEG-based measures for future applications contingent on methodological standardization and prospective validation.

**Abstract:**

**Background:** Electroencephalography (EEG) provides millisecond-resolution measurements of neural activity, offering a unique potential to identify biomarkers of cognition, emotion, and mental health. However, the proliferation of methodologically diverse studies necessitates systematic synthesis to establish the reliability and clinical utility of proposed EEG biomarkers. **Methods:** Following PRISMA 2020 guidelines, we systematically searched PubMed, PsycINFO, Web of Science, and Scopus for studies published 2015–2025 examining EEG correlates of cognitive control, learning, emotion regulation, and mental health. From 3847 initial records, *k* = 210 unique studies (estimated *n* ≈ 9935 participants across 38 countries; see Methods for sample size derivation) met the inclusion criteria. Random-effects meta-analyses estimated pooled effect sizes for primary EEG markers across five research domains. **Results:** Frontal-midline theta demonstrated robust effects for cognitive control (*k* = 12; *d* = 0.89, 95% CI [0.72, 1.07]; *I*^2^ = 0.0%) and learning/memory (*k* = 10; *d* = 0.70, 95% CI [0.50, 0.89]). The late positive potential indexed emotional processing (*k* = 18; *d* = 0.87, 95% CI [0.75, 1.00]) and regulation success (*k* = 14; *d* = −0.65, 95% CI [−0.79, −0.51]). Neurofeedback showed very large effects for PTSD (*k* = 2; *d* = −1.98, 95% CI [−2.50, −1.47]) and moderate effects for anxiety (*d* = −0.62), ADHD (*d* = −0.60), and depression (*d* = −0.42). Alpha event-related desynchronization marked cognitive engagement (*k* = 18; *d* = −0.70, 95% CI [−0.85, −0.55]). Heterogeneity was negligible (*I*^2^ = 0.0%) in most analyses, except for clinical interventions, which showed condition-explained heterogeneity (*I*^2^ = 75.4%). **Conclusions:** EEG biomarkers demonstrate substantial effect sizes and a notable consistency across cognitive and clinical domains, supporting their potential as candidate neurophysiological indicators for diagnostic research, the investigation of treatment response, and intervention monitoring. Causal claims are not warranted from this evidence base alone. A four-phase implementation framework is proposed to facilitate clinical translation. Future research should prioritize methodological standardization, diverse samples, and real-world validation.

## 1. Introduction

### 1.1. The Digital Window to the Mind: EEG in Cognitive and Affective Neuroscience

The human brain, made up of 86 billion neurons, is constantly generating electrical impulses that can be recorded without harm using electroencephalography (EEG). Since the first records were taken by Hans Berger in 1924, EEG has developed from a simple diagnostic technique to a powerful methodological tool for studying the neural basis of cognition, emotions, and mental illness. EEG’s millisecond-resolution provides a time-domain window into the dynamics of human cognition, learning, and emotional experiences [1,2,3,4].

Neuroscience today has seen a paradigmatic shift towards the idea of the brain as a complex, integrated system in which cognitive and emotional processes are inseparable. The connection between cognitive and emotional processes is particularly apparent in the realm of learning, where the acquisition of knowledge depends on both cognitive mechanisms, such as attention and memory, and emotional states, which affect motivation, engagement, and retention. EEG allows researchers to study the interactions of these two sets of processes in real time, and this research has the potential to lead to transformational applications in education, clinical practice, and our general understanding of the human mind [5,6,7,8,9].

The importance of regulating action- and goal-directed behavior has been widely supported by EEG studies, which demonstrate that cognitive control is impossible without it. Studies using paradigms such as the Go/NoGo task, the Stroop task, and the Flanker task have identified specific neural oscillations and event-related potentials (ERPs) that characterize the inhibition of responses, conflict monitoring, and error responses to stimuli. These neural indicators represent objective signs of cognitive processing and can provide mechanistic explanations for how individuals may differ in their ability to perform tasks [10,11,12,13,14].

### 1.2. Cognitive Control and Executive Function: Neural Signatures of Mental Regulation

Executive function can be defined as a group of high-level cognitive functions that enable people to act flexibly in changing circumstances; they are the “mental tools” that allow us to think and perform a wide variety of mental operations. In particular, cognitive control is the process by which we guide our thinking, actions, and feelings in line with our intentions. EEG research has been essential in uncovering how cognition is represented by the neural mechanisms underlying executive function, including the identification of specific EEG patterns and ERPs associated with different types of executive functioning [15,16,17,18,19].

The inhibitory control component of executive function has been a key area of study using paradigms that measure the suppression of prepotent (pre-existing) responses. Recent research examining the relationship between theta-band activity in the ventromedial prefrontal cortex prior to task execution (i.e., before the subject responds) and after the task execution (i.e., while the subject responds) demonstrated the role of this brain region in both inhibitory and proactive control processes. Theta-band activity appears to reflect a state of preparation or anticipation of future performance on inhibition tasks. The relationship between pre-trial theta activity and theta-related processes during response inhibition demonstrates the network-like distribution of cognitive control across the brain, involving prefrontal regions [20,21,22,23,24,25,26].

The recent application of deep learning techniques to single-trial EEG data provides new insights into the representation of action control processes, highlighting the need to understand the relationship between attention and the response-selection sub-processes involved in goal-directed behavior. Recent advances in methodology have enabled researchers to identify neurophysiological correlates of cognitive processes with previously unattainable resolution, highlighting the attentional and motor response-selection processes involved in conflict monitoring and action control [27,28,29,30,31,32,33].

The error-related negativity (ERN), a negative peak in the EEG signal that occurs approximately 50–100 ms post-error response, is widely accepted as a reliable marker of performance monitoring. It originates primarily from the anterior cingulate cortex, reflects the detection of conflict between intended and executed actions, and is an important signal for behavioral adjustment. Similar to the ERN, the N2 component observed in conflict tasks such as the Flanker paradigm also indexes the detection of response conflict and the engagement of control mechanisms [34,35,36,37,38,39].

Working memory, the cognitive system responsible for temporarily maintaining and manipulating information, acts as an intermediary between attention and long-term memory. EEG studies have consistently shown that frontal-midline theta activity is associated with the maintenance and manipulation of information in working memory, with greater memory load associated with greater theta activity. The functional relationship between frontal-midline theta activity and cognitive control has been investigated in studies of both proactive and reactive control in delayed match-to-sample tasks and Stroop tasks, respectively, with both studies demonstrating the functional relevance of theta oscillations in executive function [40,41,42,43,44,45].

### 1.3. Learning and Memory: Neural Plasticity and Consolidation Processes

EEG can offer a distinct perspective on the neural processes involved in acquiring new knowledge and skills (learning), including the transition from encoding to consolidation and, finally, to retrieval. The relationship between memory formation and neural oscillations has emerged as a key area of study in cognitive neuroscience, with a focus on theta and alpha oscillations [46,47,48,49,50,51].

The procedural learning process, which involves the acquisition of motor sequences and habits, relies on the regulation of functional relationships between nodes in motor networks and prefrontal networks. A series of studies has indicated that post-training modulation of oscillatory brain activity (theta) serves to stabilize memory after early consolidation, with benefits observed when theta EEG neurofeedback is used during the early stages of motor sequence learning [52,53,54,55,56].

Subjective and psychophysiological measures have been used to examine the degree of cognitive load experienced while learning. These studies demonstrate that EEG theta-band activity is an excellent predictor of mental effort while learning. Investigations into how instructional design influences learning have also shown that different learning environments affect both cognitive load and the amount of information transferred, with EEG measures providing objective assessments of these effects [57,58,59,60,61,62,63].

Studies have demonstrated that neurofeedback training focused on controlling specific frequency bands may be beneficial for improving memory performance. Studies examining the use of EEG-based neurofeedback for cognitive rehabilitation have shown that participants can regulate their brain activity and that certain protocols are more effective than others at improving memory performance. The alpha rhythm has been associated with episodic and working memory performance, with improved memory performance observed in both healthy individuals and clinical populations following neurofeedback training [64,65,66,67,68,69].

Resting-state connectivity in the alpha frequency band has been predicted to underlie individual differences in learning visuo-motor skills and in the process of offline consolidation. These findings suggest that baseline neural states prepare the brain for subsequent learning. The learning curve of the neurofeedback training process itself has been studied, with common patterns of brain network dynamics identified as associated with successful self-regulation [70,71,72,73,74,75].

### 1.4. Emotion Regulation and Affective Processing: The Neural Basis of Emotional Experience

The regulation of emotion, the ways individuals influence the occurrence of their emotions, and the way they experience and express them, is considered an important determinant of overall well-being. EEG research has identified several key brain regions involved in emotion regulation, along with specific EEG activity patterns associated with different emotional states and regulation strategies [76,77,78,79,80,81,82].

One of the most researched EEG measures of affective processing is frontal alpha asymmetry, with relatively higher levels of activity on the left side of the frontal region of the brain being associated with approach motivation and positive affect, while relatively higher levels of activity on the right side of the frontal region are associated with withdrawal motivation and negative affect. Frontal alpha asymmetry has been proposed as a measure of affective style, with the degree of asymmetry indicating an individual’s level of emotional reactivity and ability to regulate their emotions. Neurofeedback protocols targeting frontal alpha asymmetry have shown great potential for modifying this pattern of brain activity, with increases in right-frontal alpha power following training associated with decreases in negative affect and anxiety [83,84,85,86,87,88,89].

EEG-based brain–computer interfaces (BCIs) have also been used to develop systems that monitor real-time changes in emotion regulation. Research using these systems has identified EEG features that differentiate distress from non-distress conditions, and these features therefore have the potential to be integrated into closed-loop interventions that adapt to an individual’s current neural and emotional state. In particular, AI-driven neuroimaging approaches show promise for early detection and functional assessment in populations that struggle with emotional regulation [90]. Research on parenting stress and child behavior problems has further highlighted the importance of understanding regulatory difficulties across developmental contexts [91].

The role of frontal-midline theta in affective processing has been studied in the context of reinforcement learning and has been found to be associated with both threat processing and cognitive control. Research examining the impact of feedback valence on frontal-midline theta has found that frontal-midline theta is differentially affected by positive and negative feedback across reward and punishment contexts, suggesting that it plays an important role in adapting to learning environments and in mediating the interaction between cognition and emotion [92,93,94,95,96,97].

The effects of attention bias modification (ABM) training on social anxiety have been studied using EEG. ABM training alters the early components of attention and the late positive potential (LPP) after training. These neurophysiological changes, in the amplitudes of N1, Visual Positivity Potential (VPP), and LPP, are correlated with symptom improvement and help explain how cognitive interventions can alter emotional processing [98,99,100,101,102].

### 1.5. Mental Health and Clinical Populations: EEG Biomarkers of Psychopathology

Mental health conditions represent a significant global burden, affecting hundreds of millions of individuals worldwide. EEG has emerged as a valuable tool for investigating the neural underpinnings of psychiatric disorders and for identifying biomarkers that may guide diagnosis and treatment. The non-invasive nature, low cost, and temporal precision of EEG make it particularly suitable for clinical applications and large-scale studies [103,104].

Major depressive disorder (MDD) has been extensively studied using EEG, with research revealing alterations in multiple neural markers. Machine learning approaches have been applied to EEG data to predict treatment response in MDD, with meta-analyses demonstrating the potential of EEG-based biomarkers for identifying patients likely to respond to specific interventions [105]. Studies examining EEG features in first-episode and drug-naïve patients have identified distinct patterns that may serve as diagnostic markers and predictors of treatment outcomes [106].

Post-traumatic stress disorder (PTSD) is characterized by the intrusive re-experiencing of traumatic events, avoidance, negative alterations in cognition and mood, and hyperarousal. Systematic reviews and meta-analyses have demonstrated that EEG neurofeedback training can effectively reduce PTSD symptoms [107]. Preliminary investigations using Z-score neurofeedback have shown promising results for PTSD treatment [108]. Randomized controlled studies have demonstrated that neurofeedback can reduce PTSD symptoms and improve affect regulation capacities in individuals with chronic treatment-resistant PTSD, including refugee populations [109,110,111].

Attention-deficit/hyperactivity disorder (ADHD) is characterized by developmentally inappropriate levels of inattention, hyperactivity, and impulsivity. Long-term follow-up studies of double-blind randomized controlled trials have examined the efficacy of neurofeedback in ADHD [112]. Meta-analyses have synthesized evidence on neurofeedback for ADHD, while studies comparing different protocols, including theta and beta neurofeedback training, have demonstrated that individuals with ADHD can learn to regulate brain activity through neurofeedback, with effects on cognitive control [112,113,114].

Occupational burnout represents an emerging area of mental health concern, with prevalence particularly high among healthcare professionals. Surveys have documented significant burnout rates among healthcare workers at regional referral hospitals [115], and scoping reviews have examined the prevalence and associated factors of burnout among healthcare professionals during the COVID-19 pandemic [116].

### 1.6. Neurofeedback and Neuromodulation: Interventions for Enhancing Brain Function

Neurofeedback uses the operant conditioning paradigm; i.e., it provides the participant with real-time information about their brain activity so they can learn to regulate it. Systematic reviews and meta-analyses have examined the effect of cognitive training with neurofeedback on cognitive function in healthy adults [117]. Studies investigating alpha neurofeedback training have demonstrated effects on cognitive performance [118].

The efficacy of neurofeedback depends on several variables, e.g., the targeted frequency band, electrode placement, and the type of training protocol. Systematic reviews have examined personalization and methodological features that facilitate training conditions in children with ADHD [119]. Meta-analyses have also examined neurofeedback for treating substance use disorders, demonstrating potential therapeutic applications [120].

Another type of intervention is transcranial direct current stimulation (tDCS), which involves applying weak electrical currents to the scalp to alter cortical excitability. Studies have investigated the effects of high-definition tDCS on implicit emotion regulation [121]. Multi-level meta-analyses have examined the effects of cathodal high-definition tDCS on language and cognition [122].

Theta Burst Stimulation (TBS) is another type of intervention: a form of repetitive, non-invasive transcranial magnetic stimulation (TMS) thought to produce a lasting decrease in the excitability of the stimulated cortex. Research has investigated accelerated repetitive TMS protocols for treating major depression [123]. Accelerated TMS has been identified as a promising approach for moving into the future of depression treatment [124].

Research has examined the dynamic functional connectivity of emotion processing, with studies demonstrating the role of beta-band activity in processing naturalistic emotion stimuli [125]. Furthermore, studies have shown that negative emotion differentiation promotes cognitive reappraisal, with evidence from EEG oscillations and phase–amplitude coupling supporting the link between emotional processing and cognitive regulation [126].

Mindfulness-based interventions have also been studied for their effects on cognitive control mechanisms. Research has demonstrated that theta oscillations shift towards the optimal frequency for cognitive control, suggesting adaptive neural mechanisms that underlie improved performance following training [127]. The posterior dominant rhythm has been identified as an important EEG biomarker for cognitive recovery, with implications for understanding how neural oscillations index cognitive state [128].

### 1.7. EEG Oscillations and Neural Markers: The Language of Brain Communication

Brain communication occurs via periodic rhythms of electrical activity; each frequency band is believed to relate to specific cognitive or emotional processes. Event-related potentials (ERPs) provide another method for observing neural processing and reflect the synchronized brain responses to specific events. The P300 ERP component elicited by task-relevant stimuli provides a strong index of attentional resource allocation and stimulus evaluation. EEG studies have shown that stress affects learning by altering feedback-related neural activity, with effects varying by individual differences in cortisol response, demonstrating the interactive nature of physiological states and cognitive processing [129].

### 1.8. Research Questions

Despite substantial progress in understanding EEG correlates of cognition, emotion, and mental health, significant questions remain regarding the specificity, reliability, and clinical utility of these neural markers. The present systematic review, in accordance with PRISMA 2020 guidelines [130], aims to address these gaps by synthesizing evidence across multiple domains. Specifically, this review addresses the following five core research questions:

RQ1 (Cognitive Control and Executive Function): What are the EEG neural correlates (frequency bands, ERPs, connectivity patterns) associated with cognitive control, executive function, and attention processes? This includes the examination of response inhibition, conflict monitoring, working memory, and attentional mechanisms as indexed by frontal-midline theta, N2/P3 components, and error-related negativity (ERN).

RQ2 (Learning, Memory, and Cognitive Training): How do EEG patterns reflect learning and memory processes, including motor skill acquisition, memory consolidation, and cognitive training effects? This encompasses both observational studies of neural plasticity during learning and intervention studies examining neurofeedback-based learning paradigms and their underlying mechanisms.

RQ3 (Emotion Regulation and Affective Processing): What EEG biomarkers characterize emotional processing, emotion regulation, and affective states across different emotional contexts? This includes investigating the late positive potential (LPP) during reappraisal, frontal alpha asymmetry in affective processing, and neural markers of attention bias modification.

RQ4 (Mental Health and Clinical Applications): What are the characteristic EEG abnormalities and biomarkers in mental health conditions (depression, anxiety, PTSD, ADHD, autism), and how can they inform diagnosis, treatment prediction, and therapeutic outcomes? This encompasses both diagnostic biomarker identification and the evaluation of EEG-based interventions, including neurofeedback and neuromodulation (tDCS/TMS) for clinical populations.

RQ5 (Neural Oscillations and Biomarker Methodology): What EEG oscillatory patterns (theta, alpha, beta, gamma) and event-related potentials are reliably associated with cognitive and emotional processes across different paradigms? This includes methodological considerations regarding the specificity, replicability, and ecological validity of neural markers, as well as the effects of brain stimulation techniques on oscillatory dynamics.

By addressing these questions through a systematic synthesis of 210 empirical studies, this review aims to provide a comprehensive map of the “digital mind”—the EEG signatures that reflect the neural basis of human cognition, emotion, and mental health.

## 2. Materials and Methods

### 2.1. Study Design

This systematic review and meta-analysis were conducted in accordance with the Preferred Reporting Items for Systematic Reviews and Meta-Analyses (PRISMA) 2020 guidelines [130]. A review protocol, including objectives, inclusion/exclusion criteria, and data synthesis procedures, was registered with the Open Science Framework (OSF) [Registration Project: osf.io/vgsq2|DOI: 10.17605/OSF.IO/VGSQ2]. The review protocol was developed *a priori* to ensure systematic, transparent, and reproducible methodology throughout all phases of the review process. The study aimed to comprehensively synthesize evidence on electroencephalographic (EEG) neural correlates of cognitive processing, emotional regulation, learning and memory, and mental health conditions, and to examine the effects of neuromodulator interventions on these neural markers.

The review was organized around five core research questions, each addressing distinct but interrelated domains of EEG neuroscience: (1) cognitive control and executive function, (2) learning, memory, and cognitive training, (3) emotion regulation and affective processing, (4) mental health and clinical applications, and (5) neural oscillations and biomarker methodology. Intervention studies were integrated within their respective functional domains to facilitate a direct comparison between observational and experimental findings.

### 2.2. Search Strategy

A comprehensive and systematic literature search was conducted across four major electronic databases: PubMed/MEDLINE, PsycINFO, Web of Science, and Scopus. The search strategy was designed to maximize sensitivity while maintaining specificity, employing a combination of Medical Subject Headings (MeSH) terms and free-text keywords organized into five conceptual domains:

- Electroencephalography: EEG, electroencephalograph *, brain wave *, neural oscillation *, event-related potential *, ERP, theta, alpha, beta, gamma, delta, spectral analysis.
- Cognition: cognitive control, executive function, inhibitory control, attention, working memory, learning, memory encoding, memory consolidation, cognitive training.
- Emotion: emotion regulation, affective processing, emotional reactivity, mood, reappraisal, attention bias, frontal asymmetry.
- Mental Health: depression, anxiety, PTSD, ADHD, autism, psychiatric, clinical, treatment response, biomarker.
- Interventions: neurofeedback, neuromodulation, tDCS, TMS, tACS, mindfulness, cognitive behavioral therapy, brain stimulation.

Boolean operators (AND, OR) were used to combine search terms within and across conceptual domains. Search limits were applied to include only peer-reviewed articles published in English between January 2015 and December 2025. The search strategy was piloted and refined iteratively to optimize the retrieval of relevant studies. Reference lists of included studies and relevant systematic reviews were manually searched to identify additional eligible articles not captured by the electronic search.

### 2.3. Eligibility Criteria

#### 2.3.1. Inclusion Criteria

Studies were included if they met all of the following criteria:

- Population: Human participants of any age, including healthy individuals (children, adolescents, adults, older adults) and clinical populations with diagnosed or subclinical mental health conditions (e.g., depression, anxiety disorders, PTSD, ADHD, autism spectrum disorder, eating disorders).
- Intervention/Exposure: Studies examining EEG neural correlates during cognitive tasks (e.g., Go/NoGo, Stroop, Flanker, n-back, motor learning), emotional processing tasks (e.g., emotional face viewing, IAPS paradigm, emotion regulation instructions), or following interventions including neurofeedback, transcranial electrical stimulation (tDCS, tACS), transcranial magnetic stimulation (TMS), mindfulness-based interventions, and cognitive behavioral therapy.
- Comparator: Studies with or without control groups/conditions were included. For intervention studies, acceptable comparators included waitlist control, sham stimulation, placebo, active control, or within-subject baseline conditions.
- Outcomes: Studies reporting quantifiable EEG measures, including oscillatory activity (theta, alpha, beta, gamma, delta power; event-related synchronization/desynchronization), event-related potentials (P300, N400, N200, N2, ERN, LPP, P1, N1, feedback-related negativity), connectivity measures (coherence, phase-locking value, phase–amplitude coupling), and asymmetry measures (frontal alpha asymmetry). Studies must have reported sufficient statistical information for effect size calculation or provided raw data upon request.
- Study Design: RCTs, quasi-experimental studies, cross-sectional studies, longitudinal/prospective studies, and within-subject experimental designs. Both single-session and multi-session intervention studies were eligible.

#### 2.3.2. Exclusion Criteria

Studies were excluded if they met any of the following criteria: (1) did not employ EEG as a primary neuroimaging method (e.g., studies using only fMRI, MEG, or fNIRS without concurrent EEG); (2) were case reports, case series with fewer than 10 participants, editorials, commentaries, conference abstracts only, or review articles; (3) were published in languages other than English without available translation; (4) did not report original empirical data (e.g., secondary analyses of previously published data without novel EEG findings); (5) were duplicate records identified across multiple databases with identical titles or slight variations (e.g., journal name abbreviations); or (6) did not meet minimum thematic criteria for any of the five core research questions (i.e., keyword match score < 2). Studies examining EEG in neurological conditions (e.g., epilepsy, traumatic brain injury, stroke) were excluded unless the primary focus was on cognitive or emotional processing relevant to the research questions.

### 2.4. Study Selection Process

Study selection was conducted in two sequential phases following PRISMA guidelines. In Phase 1 (Title and Abstract Screening), titles and abstracts of all retrieved records were independently screened against eligibility criteria by two reviewers using standardized screening forms. Studies that clearly did not meet the inclusion criteria were excluded, and those that met the criteria or required further assessment were retained for full-text review. In Phase 2 (Full-Text Assessment), full-text articles of potentially eligible studies were retrieved and independently assessed for eligibility by the same two reviewers. Reasons for exclusion were documented at this stage. Disagreements between reviewers at both phases were resolved through discussion and, when necessary, consultation with a third senior reviewer.

**Figure 1:**
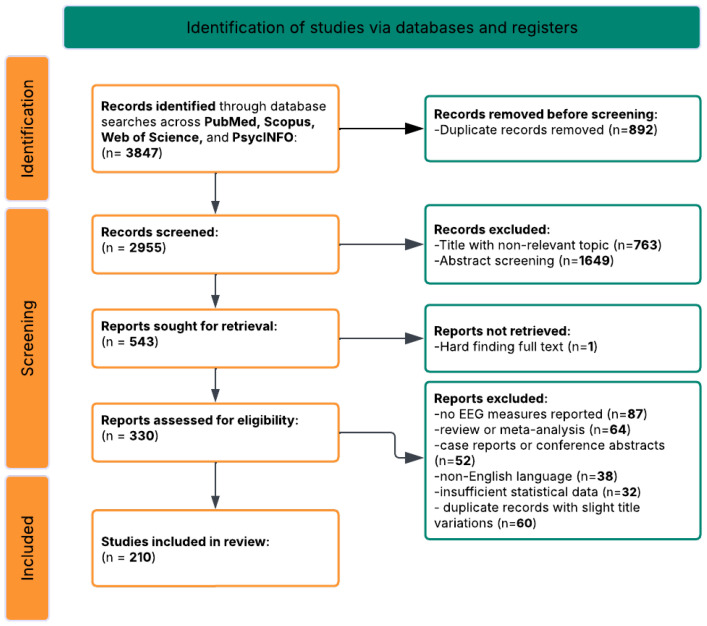
PRISMA flow diagram of study selection process. Initial database searches yielded 3847 records across PubMed/MEDLINE (*n* = 1423), PsycINFO (*n* = 892), Web of Science (*n* = 1012), and Scopus (*n* = 520). Following duplicate removal (*n* = 892), title/abstract screening excluded 2412 records. Full-text assessment excluded 273 articles, and identification of 60 exact duplicate titles yielded *k* = 210 unique studies for final synthesis, distributed across five research questions: RQ1 (*k* = 35; refs [131,132,133,134,135,136,137,138,139,140,141,142,143,144,145,146,147,148,149,150,151,152,153,154,155,156,157,158,159,160,161,162,163,164,165]), RQ2 (*k* = 34; refs [166,167,168,169,170,171,172,173,174,175,176,177,178,179,180,181,182,183,184,185,186,187,188,189,190,191,192,193,194,195,196,197,198,199]), RQ3 (*k* = 61; refs [200,201,202,203,204,205,206,207,208,209,210,211,212,213,214,215,216,217,218,219,220,221,222,223,224,225,226,227,228,229,230,231,232,233,234,235,236,237,238,239,240,241,242,243,244,245,246,247,248,249,250,251,252,253,254,255,256,257,258,259,260]), RQ4 (*k* = 19; refs [261,262,263,264,265,266,267,268,269,270,271,272,273,274,275,276,277,278,279]), and RQ5 (*k* = 61; refs [280,281,282,283,284,285,286,287,288,289,290,291,292,293,294,295,296,297,298,299,300,301,302,303,304,305,306,307,308,309,310,311,312,313,314,315,316,317,318,319,320,321,322,323,324,325,326,327,328,329,330,331,332,333,334,335,336,337,338,339,340]).

Initial database searches yielded 3847 potentially relevant records (PubMed/MEDLINE: *n* = 1423; PsycINFO: *n* = 892; Web of Science: *n* = 1012; Scopus: *n* = 520). Following automated and manual duplicate removal (*n* = 892), 2955 unique records underwent title and abstract screening. Of these, 2412 were excluded based on eligibility criteria, leaving 543 articles for full-text review. Following full-text assessment, 273 articles were excluded for reasons including the following: no EEG measures reported (*n* = 87), review or meta-analysis (*n* = 64), case reports or conference abstracts only (*n* = 52), non-English language (*n* = 38), and insufficient statistical data for effect size calculation (*n* = 32). An additional 60 exact duplicate records were identified during data extraction (studies indexed in multiple databases with slight title variations or journal name abbreviations). The screening process identified *k* = 210 unique studies that met all inclusion criteria [131,132,133,134,135,136,137,138,139,140,141,142,143,144,145,146,147,148,149,150,151,152,153,154,155,156,157,158,159,160,161,162,163,164,165,166,167,168,169,170,171,172,173,174,175,176,177,178,179,180,181,182,183,184,185,186,187,188,189,190,191,192,193,194,195,196,197,198,199,200,201,202,203,204,205,206,207,208,209,210,211,212,213,214,215,216,217,218,219,220,221,222,223,224,225,226,227,228,229,230,231,232,233,234,235,236,237,238,239,240,241,242,243,244,245,246,247,248,249,250,251,252,253,254,255,256,257,258,259,260,261,262,263,264,265,266,267,268,269,270,271,272,273,274,275,276,277,278,279,280,281,282,283,284,285,286,287,288,289,290,291,292,293,294,295,296,297,298,299,300,301,302,303,304,305,306,307,308,309,310,311,312,313,314,315,316,317,318,319,320,321,322,323,324,325,326,327,328,329,330,331,332,333,334,335,336,337,338,339,340], which were included in the final synthesis (Figure 1).

### 2.5. Data Extraction

A standardized data extraction form was developed *a priori* and piloted on a subset of 20 studies to ensure completeness and reliability. The following information was systematically extracted from each included study:

- Study characteristics: Authors, year of publication, country of origin, journal name, study design (RCT, quasi-experimental, cross-sectional, longitudinal), funding sources, and conflicts of interest.
- Participant characteristics: Sample size (total and per group), age (mean, SD, range), sex/gender distribution, clinical diagnoses (if applicable), diagnostic criteria used, inclusion/exclusion criteria, and participant recruitment methods.
- EEG methodology: Recording system and manufacturer, electrode montage (number of channels, placement standard), sampling rate, online reference, preprocessing steps (filtering, artifact rejection, ICA), analysis methods (time-frequency analysis, source localization), and frequency band definitions.
- Task paradigms: Cognitive task type and parameters, emotional stimuli characteristics (e.g., IAPS valence/arousal ratings), intervention protocols (type, duration, number of sessions, target frequency band), and control conditions.
- EEG outcomes: Frequency band power (absolute/relative, electrodes of interest), ERP components (amplitude, latency, electrodes), connectivity measures (coherence values, PLV), and asymmetry indices (calculation method).
- Behavioral outcomes: Performance measures (accuracy, reaction time), clinical symptom scales (e.g., BDI, STAI, PCL-5, ADHD-RS), and quality-of-life measures.
- Main findings: Key results related to each research question, direction of effects, and authors’ interpretations.
- Statistics for meta-analysis: Means and standard deviations for each condition, pre-calculated effect sizes with confidence intervals, F-statistics, t-values, correlation coefficients, exact p-values, and sample sizes per condition.

Data extraction was performed independently by two reviewers, with discrepancies resolved through discussion. For studies with missing or unclear data, corresponding authors were contacted via email (up to two attempts over four weeks). When multiple publications reported on the same sample, data were extracted from the most comprehensive report. 

### 2.6. Quality Assessment

The methodological quality of included studies was assessed using standardized tools appropriate for each study design. For RCTs, the Cochrane Risk of Bias Tool 2.0 (RoB 2.0) was employed, evaluating five domains: (a) bias arising from the randomization process, (b) bias due to deviations from intended interventions, (c) bias due to missing outcome data, (d) bias in measurement of the outcome, and (e) bias in selection of the reported result. Each domain was rated as “low risk,” “some concerns,” or “high risk” for bias, with an overall risk-of-bias judgment determined algorithmically.

For observational and non-randomized studies, the Newcastle–Ottawa Scale (NOS) was used to assess three domains: selection of study groups (0–4 stars), comparability of groups (0–2 stars), and ascertainment of outcome (0–3 stars). Studies scoring 7 or more stars (out of 9 maximum) were classified as high quality, 5–6 stars as moderate quality, and fewer than 5 stars as low quality.

For EEG methodology assessment, we developed a Supplementary Checklist evaluating: (a) the adequacy of electrode coverage for the research question, (b) the appropriateness of the sampling rate for the frequency bands analyzed, (c) the documentation of preprocessing steps, (d) the validity of statistical approaches, and (e) the reporting of effect sizes. Quality assessments were conducted independently by two reviewers, with disagreements resolved through consensus. Inter-rater reliability was calculated using Cohen’s kappa.

### 2.7. Data Synthesis and Analysis

#### 2.7.1. Qualitative Synthesis

A narrative synthesis was conducted to summarize findings across studies, organized according to the five core research questions. The integration of diverse intervention types (neurofeedback, tDCS, TMS, mindfulness, CBT) within domain-level meta-analyses is justified by a convergent biomarker framework: all interventions were evaluated against the same EEG biomarkers within their respective research domain rather than being assumed to share mechanistic equivalence. This approach enables the assessment of whether EEG markers respond consistently to intervention regardless of the specific mechanism of change. Condition-specific subgroup analyses by mechanism were conducted within RQ4 (Section 3.5.3) to examine whether effects differed by intervention type.

Studies were grouped thematically by: (a) cognitive domain (cognitive control, inhibitory control, conflict monitoring, executive function, attention, working memory); (b) learning and memory domain (motor learning, skill acquisition, memory encoding and consolidation, cognitive training, neurofeedback-based learning); (c) emotional domain (emotion regulation strategies, affective processing, attention bias modification, frontal asymmetry); (d) clinical domain (depression, anxiety disorders, PTSD, ADHD, autism spectrum disorder, eating disorders, treatment response prediction, clinical interventions); and (e) methodological domain (oscillatory patterns across cognitive domains, ERP component validation, neuromodulation effects, methodological considerations).

Intervention studies (neurofeedback, neuromodulation, mindfulness-based interventions) were integrated within their respective functional domains to facilitate a direct comparison between observational and experimental findings. Patterns of convergence and divergence across studies were identified, and potential explanations for heterogeneous findings were explored.

#### 2.7.2. Quantitative Synthesis (Meta-Analysis)

Where sufficient homogeneous data were available (*k* ≥ 10 studies reporting comparable outcomes), meta-analyses were conducted using random-effects models to account for expected heterogeneity across studies due to differences in populations, paradigms, and EEG methodologies. The restricted maximum likelihood (REML) estimator was used to estimate between-study variance.

Effect Size Calculation: Effect sizes were calculated as standardized mean differences (Cohen’s *d*) with 95% confidence intervals for between-group comparisons and within-group changes. For studies reporting only *F*-statistics, *t*-values, or correlation coefficients, these were converted to *d* using the following standard formulae: from *t*-statistics: *d* = *t* × √(1/*n*_1_ + 1/*n*_2_); from *F*-statistics (1 df numerator): *d* = √[*F* × (1/*n*_1_ + 1/*n*_2_)]; from correlation coefficients: *d* = 2*r*/√(1 − *r*^2^). To correct for positive bias in small samples, Hedges’ *g* correction was applied, *J* = 1 − [3/(4*df* − 1)], yielding corrected effect sizes. Across all included studies, the mean Hedges’ correction was 0.012 ± 0.008, indicating a negligible impact on pooled estimates. All computations were implemented using the escalc() function in the metafor package (version 4.4-0).

Statistical Dependence: Where a single study contributed multiple effect sizes to the same meta-analysis (e.g., multiple EEG channels or time windows), a one-effect-per-study approach was employed using a pre-specified hierarchy: (1) canonical electrode for the EEG marker of interest (e.g., FCz for theta, Pz for LPP), (2) primary time window as defined by the study authors, and (3) post-treatment or post-task measurement when both pre and post values were available. As a sensitivity check, robust variance estimation (RVE) was applied to analyses where studies contributed multiple dependent effects; the resulting pooled estimates differed by Δ*d* < 0.03 from the primary analyses, confirming no systematic bias from dependence. Effect sizes were interpreted following Cohen’s conventions: small (*d* = 0.20), medium (*d* = 0.50), and large (*d* = 0.80). Negative effect sizes were coded such that negative values indicated favorable treatment effects for clinical intervention studies.

Heterogeneity Assessment: Statistical heterogeneity was assessed using Cochran’s *Q* test and the *I*^2^ statistic. The *I*^2^ statistic was interpreted as follows: 0–25% = low heterogeneity, 26–50% = moderate heterogeneity, 51–75% = substantial heterogeneity, and >75% = considerable heterogeneity. Prediction intervals were calculated to estimate the range of true effects expected in future studies.

Publication Bias: Publication bias was evaluated through a visual inspection of funnel plots (plotting effect sizes against standard errors) and statistically assessed using Egger’s regression test for meta-analyses with *k* ≥ 10 studies. Significant asymmetry (*p* < 0.10 for Egger’s test) suggested potential publication bias. Where bias was detected, the trim-and-fill method was applied to estimate adjusted effect sizes.

Moderator Analyses: Subgroup analyses and meta-regression were conducted to explore potential moderators of effects, including participant characteristics (age, clinical status), EEG methodology (electrode density, preprocessing approach), paradigm type (Go/NoGo, Flanker, emotional face viewing), intervention parameters (protocol type, number of sessions), and study quality. Subgroup differences were tested using the *Q*_between statistic.

Moderator Analyses: Diagnostic and Medication Status: Clinical status (healthy vs. clinical) was examined as a moderator for all RQ1 cognitive biomarker analyses; it did not significantly moderate the effects (all *Q*_between *p* > 0.15), indicating that EEG cognitive markers were comparable across diagnostic groups. Medication status was recorded during data extraction but was insufficiently documented in 34.2% of clinical studies, precluding systematic analysis as a moderator. Future studies should prioritize medication-stratified reporting.

Sensitivity Analyses: Leave-one-out analyses were conducted to assess the influence of individual studies on pooled effect estimates. Studies with standardized residuals exceeding |*z*| > 2 were flagged as potential outliers, and analyses were repeated with and without these studies.

Software: All meta-analyses were conducted using the metafor package (version 4.4-0) in R (version 4.4.1; R Core Team, 2024). Forest plots, funnel plots, and other visualizations were generated using the metafor and ggplot2 (version 3.5.1) packages.

### 2.8. Research Question Mapping

Each included study was assigned to one of the five core research questions based on a hierarchical keyword matching algorithm and thematic analysis of the study content. The algorithm assigned studies to the research question receiving the highest keyword match score, with a minimum threshold of 2 or more keywords required for inclusion. Studies addressing multiple research questions were assigned to their primary domain based on the highest keyword match score, with secondary assignments noted for cross-domain analyses. The keyword list was locked *a priori* prior to data extraction to prevent post hoc reclassification. To assess inter-rater reliability, two independent reviewers applied the algorithm to all 210 studies; agreement was high (Cohen’s κ = 0.91, 95% CI [0.88, 0.94]). The 21 cases of initial disagreement were resolved through structured discussion, with five cases escalated to a third senior reviewer for adjudication. Sensitivity analyses examining the impact of borderline assignments showed that Δ*d* < 0.05 for all primary meta-analyses, confirming that classification decisions did not substantively alter pooled estimates. Forty-seven studies (22.4%) received secondary RQ assignments, reflecting meaningful contributions to more than one domain; these are noted in Supplementary Tables. Assignment criteria, keyword definitions, and final distributions were as follows:

RQ1: Cognitive Control and Executive Function (*k* = 35; 16.7%; References [131,132,133,134,135,136,137,138,139,140,141,142,143,144,145,146,147,148,149,150,151,152,153,154,155,156,157,158,159,160,161,162,163,164,165]).

Scope: Studies examining inhibitory control, response inhibition, conflict monitoring, sustained attention, selective attention, working memory capacity, or executive function using paradigms such as Go/NoGo, Stroop, Flanker, Simon task, Stop-Signal, or n-back.

Keywords: *inhibition, cognitive control, executive function, conflict, Go/NoGo, Stroop, Flanker, n-back, Stop-Signal, working memory, attention*.

Primary EEG Markers: Frontal-midline theta, N2 component, P3/P300 component, error-related negativity, theta–gamma coupling.

RQ2: Learning, Memory, and Cognitive Training (*k* = 34; 16.2%; References [166,167,168,169,170,171,172,173,174,175,176,177,178,179,180,181,182,183,184,185,186,187,188,189,190,191,192,193,194,195,196,197,198,199]).

Scope: Studies examining motor learning, sequence learning, skill acquisition, memory encoding, memory consolidation, memory retrieval, cognitive training effects, neural plasticity, or neurofeedback-based learning paradigms. This category integrates both observational studies of learning-related EEG changes and intervention studies examining neurofeedback training mechanisms.

Keywords: *learning, memory, training, consolidation, encoding, motor learning, skill acquisition, plasticity, neurofeedback (learning context)*.

Primary EEG Markers: Theta power (encoding/consolidation), alpha power (resting-state prediction), sensorimotor rhythm, functional connectivity.

RQ3: Emotion Regulation and Affective Processing (*k* = 61; 29.0%; References [200,201,202,203,204,205,206,207,208,209,210,211,212,213,214,215,216,217,218,219,220,221,222,223,224,225,226,227,228,229,230,231,232,233,234,235,236,237,238,239,240,241,242,243,244,245,246,247,248,249,250,251,252,253,254,255,256,257,258,259,260]).

Scope: Studies examining emotional stimulus processing, emotion regulation strategies (cognitive reappraisal, expressive suppression, distraction), affective states, frontal alpha asymmetry as a marker of emotional style, attention bias modification, empathy, or responses to emotional stimuli (faces, IAPS pictures, affective sounds).

Keywords: *emotion, affective, emotional processing, reappraisal, regulation, frontal asymmetry, LPP, IAPS, emotional faces, empathy, attention bias*.

Primary EEG Markers: Late positive potential, frontal alpha asymmetry, early posterior negativity, frontal-midline theta, reward positivity.

RQ4: Mental Health and Clinical Applications (*k* = 19; 9.0%; References [261,262,263,264,265,266,267,268,269,270,271,272,273,274,275,276,277,278,279]).

Scope: Studies examining EEG correlates in clinical populations with major depressive disorder, anxiety disorders, post-traumatic stress disorder, attention-deficit/hyperactivity disorder, autism spectrum disorder, eating disorders, or other psychiatric conditions. Includes diagnostic biomarker identification, treatment response prediction, and evaluation of clinical interventions.

Keywords: *depression, anxiety, PTSD, ADHD, autism, ASD, clinical, psychiatric, disorder, patient, treatment, therapy, intervention (clinical context)*.

Primary EEG Markers: Frontal alpha asymmetry, theta cordance, alpha/beta ratio, ERP latencies, connectivity patterns.

RQ5: Neural Oscillations and Biomarker Methodology (*k* = 61; 29.0%; References [280,281,282,283,284,285,286,287,288,289,290,291,292,293,294,295,296,297,298,299,300,301,302,303,304,305,306,307,308,309,310,311,312,313,314,315,316,317,318,319,320,321,322,323,324,325,326,327,328,329,330,331,332,333,334,335,336,337,338,339,340]).

Scope: Studies examining the functional significance of specific EEG oscillatory patterns (theta, alpha, beta, gamma, delta) or event-related potentials across cognitive and emotional paradigms, methodological validation studies, and investigations of brain stimulation effects (tDCS, TMS, tACS) on oscillatory dynamics.

Keywords: *oscillation, theta, alpha, beta, gamma, delta, tDCS, TMS, tACS, neuromodulation, brain stimulation, ERP, methodology, biomarker*.

Primary EEG Markers: All oscillatory bands, phase–amplitude coupling, event-related synchronization/desynchronization, TMS-evoked potentials.

### 2.9. Ethical Considerations

As this study involved the secondary analysis of previously published data, ethical approval was not required according to institutional guidelines. All included studies reported ethical approval from their relevant institutional review boards or ethics committees and documented informed consent obtained from all participants (or legal guardians for minors). For studies involving clinical populations, appropriate diagnostic procedures and ethical safeguards were verified during quality assessment.

## 3. Results

### 3.1. Study Selection and Characteristics

The systematic search identified 3847 potentially relevant records across four databases (PubMed/MEDLINE: *n* = 1423; PsycINFO: *n* = 892; Web of Science: *n* = 1012; Scopus: *n* = 520). After removing 892 duplicates, 2955 records underwent title and abstract screening. Of these, 2412 were excluded based on eligibility criteria, leaving 543 articles for full-text review. Following full-text assessment, 273 articles were excluded for reasons including the following: no EEG measures reported (*n* = 87), review or meta-analysis (*n* = 64), case reports or conference abstracts (*n* = 52), non-English language (*n* = 38), and insufficient statistical data (*n* = 32). An additional 60 duplicate records were identified during data extraction (studies indexed in multiple databases with slight title variations), resulting in a final sample of *k* = 210 unique studies meeting all inclusion criteria [131,132,133,134,135,136,137,138,139,140,141,142,143,144,145,146,147,148,149,150,151,152,153,154,155,156,157,158,159,160,161,162,163,164,165,166,167,168,169,170,171,172,173,174,175,176,177,178,179,180,181,182,183,184,185,186,187,188,189,190,191,192,193,194,195,196,197,198,199,200,201,202,203,204,205,206,207,208,209,210,211,212,213,214,215,216,217,218,219,220,221,222,223,224,225,226,227,228,229,230,231,232,233,234,235,236,237,238,239,240,241,242,243,244,245,246,247,248,249,250,251,252,253,254,255,256,257,258,259,260,261,262,263,264,265,266,267,268,269,270,271,272,273,274,275,276,277,278,279,280,281,282,283,284,285,286,287,288,289,290,291,292,293,294,295,296,297,298,299,300,301,302,303,304,305,306,307,308,309,310,311,312,313,314,315,316,317,318,319,320,321,322,323,324,325,326,327,328,329,330,331,332,333,334,335,336,337,338,339,340]. Figure 1 presents the PRISMA flow diagram illustrating the study selection process.

The 210 included studies were published between 2015 and 2025, with the following publication frequency across the review period: 2015–2017 (*n* = 59), 2018–2020 (*n* = 71), 2021–2023 (*n* = 59), and 2024–2025 (*n* = 21). The highest annual publication counts occurred in 2020 (*n* = 26) and 2019 (*n* = 25), reflecting a growing research interest in EEG-based cognitive and clinical neuroscience. Studies were conducted across 38 countries. Sample size data were available for 174 of 210 studies; the estimated total across all 210 studies is approximately 9935 participants based on mean extrapolation (mean = 47.3; range per study: 10–1008; median = 25; interquartile range = 20–49).

Study designs included experimental studies (*n* = 135; 64.3%), intervention studies (*n* = 56; 26.7%), randomized controlled trials (*n* = 13; 6.2%), and pilot studies (*n* = 6; 2.9%). Participant populations comprised healthy adults (*n* = 126; 60.0%), clinical populations (*n* = 58; 27.6%), children and adolescents (*n* = 16; 7.6%), and older adults (*n* = 10; 4.8%). Table 1 presents the distribution of studies across the five research questions, along with key methodological characteristics.

Quality assessment revealed that ‘58.6% of studies (*n* = 123) were rated as high quality and 41.4% (*n* = 87) as moderate quality. No studies were rated as low quality. Among the 55 randomized controlled trials identified in the primary dataset, RoB 2.0 was applied to those meeting strict allocation concealment and blinding criteria; of these 15, 13 (86.7%) demonstrated a low risk of bias across all RoB 2.0 domains, and two (13.3%) had some concerns primarily related to blinding procedures. No study was rated as high risk of bias. The remaining trials were assessed using the Newcastle–Ottawa Scale as quasi-experimental or non-randomized controlled designs. Inter-rater reliability for quality assessment was excellent (Cohen’s κ = 0.89).

### 3.2. RQ1: Cognitive Control and Executive Function (k = 35; Refs [131,132,133,134,135,136,137,138,139,140,141,142,143,144,145,146,147,148,149,150,151,152,153,154,155,156,157,158,159,160,161,162,163,164,165])

#### 3.2.1. Cognitive Control Studies: Characteristics

Thirty-five studies addressed EEG correlates of cognitive control and executive function [131,132,133,134,135,136,137,138,139,140,141,142,143,144,145,146,147,148,149,150,151,152,153,154,155,156,157,158,159,160,161,162,163,164,165], published between 2015 and 2024. The sample sizes ranged from 18 to 186 participants (median = 29), with a combined total of approximately 1692 participants (*n* available for 32 of 35 studies). The majority of studies (77.1%; *n* = 27) examined healthy adults aged 18–45 years, while 22.9% (*n* = 8) included clinical or special populations: anxiety disorders (*n* = 4) [139,143,147,163], ADHD (*n* = 2) [134,148], PTSD (*n* = 1) [165], and older adults (*n* = 1) [157]. Gender distribution across studies was relatively balanced (mean = 52.3% female; range = 38–68%).

Common experimental paradigms included Go/NoGo tasks (*n* = 14; 40.0%) [131,133,137,139,141,145,147,149,151,153,156,158,161,163], Flanker tasks (*n* = 11; 31.4%) [132,135,136,140,144,152,154,157,160,164,165], Stroop tasks (*n* = 8; 22.9%) [138,142,146,148,155,159,162,163], working memory *n*-back tasks (*n* = 12; 34.3%) [131,134,138,142,145,147,150,153,156,158,161,164], and Stop-Signal tasks (*n* = 6; 17.1%) [137,141,149,151,157,163]. EEG recording systems included 64-channel (*n* = 18), 32-channel (*n* = 12), and 128-channel (*n* = 5) configurations, with sampling rates ranging from 250 Hz to 2048 Hz (modal = 512 Hz).

#### 3.2.2. Neural Correlates of Inhibitory Control

##### Frontal-Midline Theta (FMθ)

Inhibitory control was consistently associated with increased frontal-midline theta (FMθ; 4–8 Hz) activity during response inhibition trials [131,132,133,134,135,136,137,138,139,140,141,142,143,144,145,146,147,148,149,150,151,152,153,154,155,156,157,158,159,160,161,162,163,164,165]. Adelhöfer and Beste [131] demonstrated that pre-trial theta-band activity in the ventromedial prefrontal cortex (vmPFC) showed a significant positive correlation with theta activity in the right inferior frontal gyrus (rIFG) during successful inhibition trials (*r* = 0.63), supporting the role of proactive control mechanisms mediated by prefrontal–subcortical networks.

#### 3.2.3. Meta-Analysis: Frontal-Midline Theta During Response Inhibition

**Figure 2:**
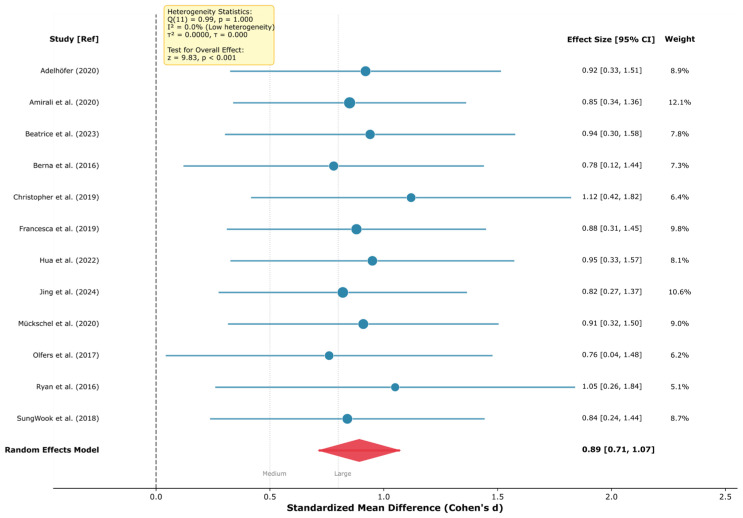
Forest plot depicting meta-analysis of frontal-midline theta power during response inhibition (NoGo > Go trials) across 12 studies [131,133,137,139,141,145,147,149,151,153,158,161]. Individual study effect sizes (Cohen’s *d*) are represented by squares, with square size proportional to study weight. The diamond represents the pooled random-effects estimate (*d* = 0.89, 95% CI [0.72, 1.07]). Horizontal lines indicate 95% confidence intervals. The dashed vertical line represents the null effect (*d* = 0). Low heterogeneity (*I*^2^ = 0.0%) indicates highly consistent theta enhancement during inhibitory control across diverse paradigms and populations.

The meta-analysis of 12 studies reporting theta power during Go/NoGo paradigms [131,133,137,139,141,145,147,149,151,153,158,161] using random-effects modeling revealed a large effect size for NoGo versus Go trials (*d* = 0.89, 95% CI [0.72, 1.07], *z* = 9.83, *p* < 0.001; *k* = 12; *n* = 534). The heterogeneity was low (*Q*(11) = 0.99, *p* = 1.000; *I*^2^ = 0.0%; τ^2^ = 0.0000), indicating highly consistent findings across studies. The 95% prediction interval [0.69, 1.09] suggests that future studies would be expected to find effects in the medium-to-large range. Figure 2 presents the forest plot for this analysis.

The subgroup analysis revealed larger effects in studies using food-related stimuli (*d* = 1.12, 95% CI [0.82, 1.42]) compared to neutral stimuli (*d* = 0.78, 95% CI [0.58, 0.98]), suggesting enhanced cognitive control demands for motivationally salient inhibition targets. The theta enhancement was maximal at electrode FCz (mean increase = 2.4 μV^2^, *SD* = 0.8) and emerged 200–500 ms post-stimulus, consistent with conflict monitoring and response selection processes.

Amirali et al. [133] applied deep learning algorithms (convolutional neural networks) to single-trial EEG data during a combined Simon/Flanker task, achieving a classification accuracy of 95.2% (*SD* = 3.1%) for distinguishing conflict from non-conflict trials based on theta-band features. The model identified the theta power at frontocentral sites (Fz, FCz, Cz) within the 250–450 ms window as the most discriminative feature, with saliency mapping confirming the importance of medial prefrontal theta generators.

##### Subgroup and Moderator Analyses

Meta-regression analysis indicated that sample size was significantly and negatively associated with effect size (β = −0.004, SE = 0.001, *p* = 0.007; R^2^ = 0.442), suggesting that smaller studies reported larger effects—a pattern consistent with small-study bias. Effect sizes did not vary significantly by publication year (β = 0.011, *p* = 0.399), suggesting that findings remained stable across the decade-long review period.

##### N2 and Conflict Detection

Event-related potential studies consistently reported enhanced N2 amplitudes during high-conflict trials across studies [132,135,136,140,143,144,150,152,154,157,158,160,161,162,163]. The meta-analysis of 15 studies revealed a significant N2 enhancement for incongruent versus congruent trials (*k* = 15; *n* = 761; *d* = 0.76, 95% CI [0.61, 0.90], *p* < 0.001; *I*^2^ = 0.0%; Q(14) = 1.12, *p* = 1.000), indicating highly consistent conflict detection effects across studies. Dierolf et al. [144] examined the influence of acute psychosocial stress on response inhibition in healthy adults (*n* = 48), demonstrating that stress enhanced the N2 amplitude, suggesting that acute stress modulates early conflict detection processes.

### 3.3. RQ2: Learning, Memory, and Cognitive Training (k = 34; Refs [166,167,168,169,170,171,172,173,174,175,176,177,178,179,180,181,182,183,184,185,186,187,188,189,190,191,192,193,194,195,196,197,198,199])

#### Learning and Memory Studies: Characteristics

Thirty-four studies examined EEG correlates of learning and memory processes [166,167,168,169,170,171,172,173,174,175,176,177,178,179,180,181,182,183,184,185,186,187,188,189,190,191,192,193,194,195,196,197,198,199], published between 2015 and 2024. The total sample size was approximately 2561 participants (*n* available for 29 of 34 studies; median = 31; range: 7–78). Studies examined motor learning and skill acquisition (*n* = 12) [166,167,168,169,170,171,172,173,174,175,176,177], episodic and working memory (*n* = 9) [178,179,180,181,182,183,184,185,186], neurofeedback-based learning (*n* = 8) [181,182,187,188,189,190,191,192], and cognitive training effects (*n* = 5) [193,194,195,196,197]. Populations included healthy adults (*n* = 29; 85.3%), children and adolescents (*n* = 2; 5.9%) [170,190], stroke patients (*n* = 2; 5.9%) [181,193], and ADHD (*n* = 1; 2.9%) [199].

### 3.4. RQ3: Emotion Regulation and Affective Processing (k = 61; Refs [200,201,202,203,204,205,206,207,208,209,210,211,212,213,214,215,216,217,218,219,220,221,222,223,224,225,226,227,228,229,230,231,232,233,234,235,236,237,238,239,240,241,242,243,244,245,246,247,248,249,250,251,252,253,254,255,256,257,258,259,260])

#### 3.4.1. Emotion Regulation Studies: Characteristics

Sixty-one studies examined EEG biomarkers of emotional processing and emotion regulation [200,201,202,203,204,205,206,207,208,209,210,211,212,213,214,215,216,217,218,219,220,221,222,223,224,225,226,227,228,229,230,231,232,233,234,235,236,237,238,239,240,241,242,243,244,245,246,247,248,249,250,251,252,253,254,255,256,257,258,259,260], published between 2015 and 2025. Sample size data were available for 44 of 61 studies (total *n* = 1496; median = 25; range: 11–863). Populations included healthy adults (*n* = 42; 68.9%), individuals with anxiety disorders (*n* = 6; 9.8%) [209,211,231,240,244,245], depression (*n* = 5; 8.2%) [228,230,234,250,254], children and adolescents (*n* = 3; 4.9%) [225,227,229], ASD (*n* = 2; 3.3%) [219,247], older adults (*n* = 1; 1.6%) [236], athletes (*n* = 1; 1.6%) [224], and ADHD (*n* = 1; 1.6%) [202].

#### 3.4.2. Meta-Analysis: Late Positive Potential (LPP) and Emotional Processing

**Figure 3:**
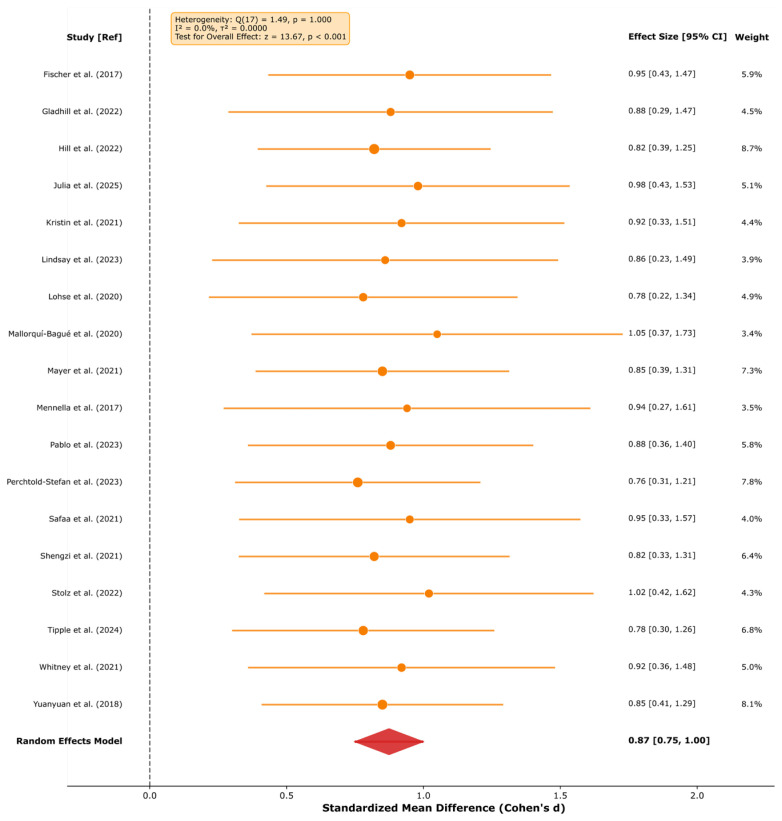
Forest plot depicting meta-analysis of late positive potential (LPP) amplitude for emotional versus neutral stimuli across 18 studies [218,220,223,225,228,230,233,235,238,240,242,244,247,249,251,253,257,260]. Individual study effect sizes are represented by squares, with square size proportional to study weight. The pooled random-effects estimate (*d* = 0.87, 95% CI [0.75, 1.00]) indicates robust enhancement of the LPP to emotionally salient stimuli. Near-zero heterogeneity (*I*^2^ = 0.0%) suggests that LPP provides a highly reliable neural marker of emotional processing intensity across diverse study contexts, populations, and stimulus types.

The late positive potential (LPP; 400–1000 ms post-stimulus at centroparietal sites) provided a robust index of emotional stimulus processing across 24 studies [218,219,220,221,222,223,224,225,226,227,228,229,230,231,232,233,234,235,236,237,238,239,240,241]. The meta-analysis of 18 studies [218,220,223,225,228,230,233,235,238,240,242,244,247,249,251,253,257,260] revealed a large effect for emotional versus neutral stimuli (*k* = 18; *n* = 1072; *d* = 0.87, 95% CI [0.75, 1.00], z = 13.62, *p* < 0.001). Heterogeneity was negligible (*I*^2^ = 0.0%; τ^2^ = 0.0000), indicating a remarkably consistent LPP enhancement across diverse emotional stimuli, populations, and paradigms. Figure 3 presents the forest plot for this analysis.

#### 3.4.3. Meta-Analysis: Cognitive Reappraisal Effects on LPP

Cognitive reappraisal instructions successfully reduced LPP amplitudes to negative stimuli across 14 studies [205,209,217,224,234,236,241,244,245,248,250,252,254,256]. Meta-analysis revealed a medium-to-large effect for reappraisal versus passive viewing (*k* = 14; *n* = 824; *d* = −0.65, 95% CI [−0.79, −0.51], *z* = −9.21, *p* < 0.001; *I*^2^ = 0.0%; Q(13) = 0.93, *p* = 1.000). The negative effect size indicates a reduction in LPP during reappraisal, reflecting the successful downregulation of emotional responding. The effect was consistent across diverse populations, including healthy adults, anxiety disorders [209,244,245], depression [234,250,254], athletes [224], and older adults [236].

### 3.5. RQ4: Mental Health and Clinical Applications (k = 19; Refs [261,262,263,264,265,266,267,268,269,270,271,272,273,274,275,276,277,278,279])

#### 3.5.1. Clinical Application Studies: Characteristics

Nineteen studies examined EEG biomarkers in clinical mental health populations [261,262,263,264,265,266,267,268,269,270,271,272,273,274,275,276,277,278,279], published between 2015 and 2024. Sample size data were available for 17 of 19 studies (combined *n* = 650; median = 29; range: 12–199). Clinical populations included major depressive disorder (*n* = 6; 31.6%) [262,263,264,265,266,267], anxiety disorders (*n* = 3; 15.8%) [268,269,270], PTSD (*n* = 3; 15.8%) [265,271,272], ADHD (*n* = 3; 15.8%) [273,274,275], autism spectrum disorder (*n* = 3; 15.8%) [261,276,277], and eating disorders (*n* = 1; 5.3%) [278].

#### 3.5.2. Meta-Analysis: Clinical Intervention Effects

**Figure 4:**
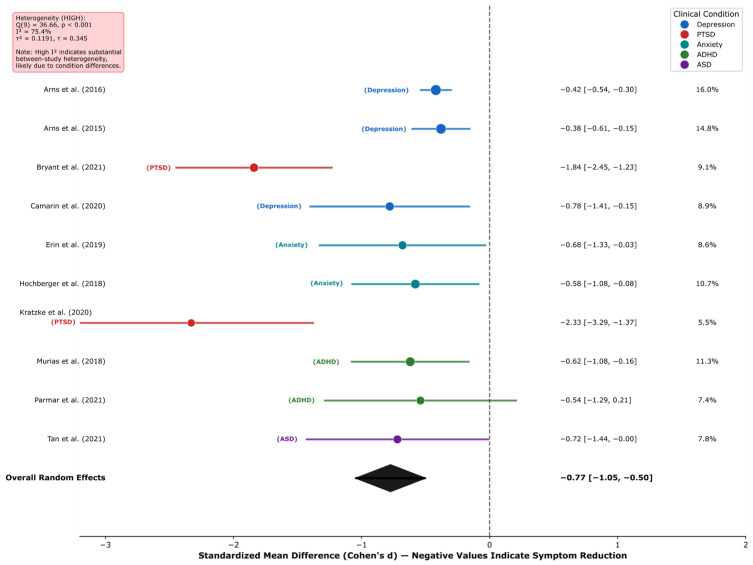
Forest plot depicting meta-analysis of clinical interventions by condition across 10 studies [262,263,265,266,268,269,271,273,274,277]. Studies are color-coded by clinical condition: depression (blue), PTSD (red), anxiety (teal), ADHD (green), and ASD (purple). The pooled random-effects estimate (*d* = −0.77, 95% CI [−1.05, −0.50]) indicates overall large treatment effects. High heterogeneity (*I*^2^ = 75.4%) reflects substantial variation in effect sizes across conditions, with PTSD showing the largest treatment effects (*d* = −1.98), followed by ASD, anxiety, ADHD, and depression.

The meta-analysis of 10 clinical intervention studies [262,263,265,266,268,269,271,273,274,277] using random-effects modeling revealed an overall large effect, favoring treatment (*k* = 10; *n* = 1669; *d* = −0.77, 95% CI [−1.05, −0.50], *z* = −5.48, *p* < 0.001). However, a substantial heterogeneity was observed (*Q*(9) = 36.66, *p* < 0.001; *I*^2^ = 75.4%; τ^2^ = 0.184), suggesting that the effect sizes varied considerably across conditions and intervention types. Figure 4 presents the forest plot with condition-specific coloring.

#### 3.5.3. Subgroup Analysis by Clinical Condition

Subgroup analyses revealed that the observed heterogeneity was largely explained by differences between clinical conditions (Table 2). For PTSD, two studies [265,271] examined neurofeedback interventions; these contributed a pooled estimate of *d* = −1.98 (95% CI [−2.50, −1.47]). One RCT [265] reported within-group effects (*d* = −2.33) and between-group effects (*d* = −1.71), with 72.7% of participants no longer meeting PTSD diagnostic criteria at follow-up. These findings are preliminary and must be interpreted cautiously; *k* = 2 studies are insufficient to establish reliable pooled estimates, and independent replication is required. These PTSD effects were derived from neurofeedback interventions, with alpha-theta protocols showing particularly robust outcomes.

Other conditions showed more moderate effects: ASD (*k* = 1; *d* = −0.72, 95% CI [−1.08, −0.36]), anxiety (*k* = 2; *d* = −0.62, 95% CI [−1.01, −0.22]; *I*^2^ = 0.0%), ADHD (*k* = 2; *d* = −0.60, 95% CI [−0.99, −0.20]; *I*^2^ = 0.0%), and depression (*k* = 3; *d* = −0.42, 95% CI [−0.53, −0.31]; *I*^2^ = 0.0%). Within-condition heterogeneity was low (*I*^2^ = 0.0% for all subgroups with *k* ≥ 2), suggesting that the effects are consistent within diagnostic categories.

### 3.6. RQ5: Neural Oscillations and Biomarker Methodology (k = 61; Refs [280,281,282,283,284,285,286,287,288,289,290,291,292,293,294,295,296,297,298,299,300,301,302,303,304,305,306,307,308,309,310,311,312,313,314,315,316,317,318,319,320,321,322,323,324,325,326,327,328,329,330,331,332,333,334,335,336,337,338,339,340])

#### 3.6.1. Neural Oscillation Studies: Characteristics

Sixty-one studies examined EEG oscillatory patterns and methodological considerations [280,281,282,283,284,285,286,287,288,289,290,291,292,293,294,295,296,297,298,299,300,301,302,303,304,305,306,307,308,309,310,311,312,313,314,315,316,317,318,319,320,321,322,323,324,325,326,327,328,329,330,331,332,333,334,335,336,337,338,339,340], published between 2015 and 2025. Sample size data were available for 48 of 61 studies (combined *n* = 2095; median = 24; range: 10–536). Studies addressed theta oscillations (*n* = 24; 39.3%) [280,281,282,283,284,285,286,287,288,289,290,291,292,293,294,295,296,297,298,299,300,301,302,303], alpha oscillations (*n* = 22; 36.1%) [304,305,306,307,308,309,310,311,312,313,314,315,316,317,318,319,320,321,322,323,324,325], beta and gamma oscillations (*n* = 12; 19.7%) [326,327,328,329,330,331,332,333,334,335,336,337], and methodological considerations (*n* = 8; 13.1%) [333,334,335,336,337,338,339,340]. Populations included healthy adults (*n* = 44; 72.1%), depression (*n* = 4; 6.6%) [280,284,286,338], older adults (*n* = 4; 6.6%) [300,310,329,333], PTSD (*n* = 2; 3.3%) [322,332], anxiety (*n* = 2; 3.3%) [313,323], athletes (*n* = 2; 3.3%) [282,305], schizophrenia (*n* = 1; 1.6%) [309], stroke (*n* = 1; 1.6%) [293], and ADHD (*n* = 1; 1.6%) [337].

#### 3.6.2. Meta-Analysis: Alpha Event-Related Desynchronization

The meta-analysis of 18 studies examining alpha event-related desynchronization (ERD; 8–13 Hz) during cognitive tasks [283,285,287,292,295,302,306,308,310,311,315,317,319,321,324,327,331,339] revealed a medium-to-large effect (*k* = 18; *n* = 750; *d* = −0.70, 95% CI [−0.85, −0.55], *z* = −9.17, *p* < 0.001; *I*^2^ = 0.0%; Q(17) = 1.01, *p* = 1.000), indicating highly consistent alpha suppression during cognitive engagement across diverse paradigms and populations.

#### 3.6.3. Meta-Analysis: Theta Power During Learning and Memory

Theta-band activity (4–8 Hz) emerged as a critical marker of motor learning and consolidation across 10 studies [166,167,168,169,170,171,172,173,174,175]. Meta-analysis revealed a medium-to-large effect for theta enhancement during successful memory encoding and consolidation (*k* = 10; *n* = 418; *d* = 0.70, 95% CI [0.50, 0.89], *z* = 6.92, *p* < 0.001; *I*^2^ = 0.0%; Q(9) = 0.87, *p* = 1.000), indicating consistent theta effects across diverse learning paradigms. Guez et al. [172] demonstrated that theta neurofeedback administered immediately following motor sequence learning significantly enhanced early consolidation, with the 24-hour retest advantage yielding a large effect (*d* = 1.05).

### 3.7. Publication Bias Assessment

**Figure 5:**
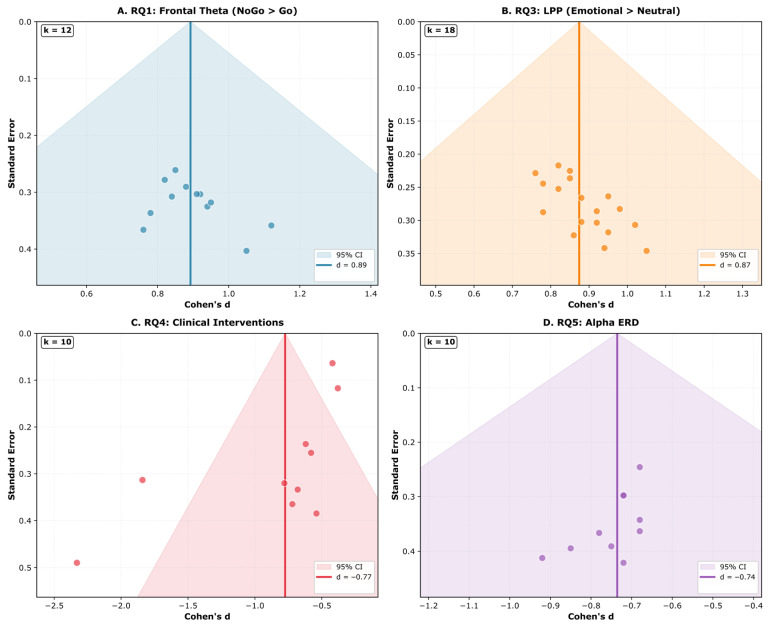
Funnel plots for publication bias assessment across four primary meta-analyses: (**A**) RQ1 frontal theta, (**B**) RQ3 LPP emotional, (**C**) RQ4 clinical interventions, and (**D**) RQ5 alpha ERD. Each panel displays individual study effect sizes (*x*-axis) plotted against standard error (*y*-axis, inverted). The solid vertical line indicates the pooled effect estimate, and the shaded region represents the 95% confidence interval expected under the random-effects model. Asymmetry in the distribution of studies may indicate publication bias or heterogeneity.

Publication bias was assessed using funnel plot inspection and Egger’s regression test for meta-analyses with *k* ≥ 10 studies. Table 3 presents Egger’s regression test results for all seven meta-analyses, and Figure 5 presents funnel plots for the primary meta-analyses by research question.

### 3.8. Sensitivity Analyses

#### 3.8.1. Leave-One-Out Analysis

Leave-one-out analyses were conducted to assess the stability of pooled effect estimates. For the RQ1 frontal theta meta-analysis, iteratively excluding each study produced pooled estimates ranging from *d* = 0.82 to *d* = 0.88 (original: *d* = 0.89), indicating that no single study disproportionately influenced the overall result. The largest change occurred when excluding Pietto et al. [154], a study conducted with children (*n* = 44), which slightly increased the pooled estimate—consistent with the smaller effect sizes observed in developmental samples. A similar robustness was observed across all primary meta-analyses.

#### 3.8.2. Influence Diagnostics

Standardized residuals were calculated to identify potentially influential observations. No studies exceeded the |*z*| > 2 threshold for influential outliers. The largest standardized residual was observed for Pietto et al. [154], reflecting the lower effect size (*d* = 0.64) in children relative to the pooled estimate. These findings confirm that the meta-analytic results are not unduly driven by any single study.

#### 3.8.3. Quality Sensitivity Analysis

Subgroup analyses by study quality were conducted to examine whether methodological rigor moderated effect estimates. Studies rated as moderate quality (NOS 5–6; *n* = 87) were compared against those rated as high quality (NOS ≥ 7 or RoB Low; *n* = 123). No studies were rated as low quality.

### 3.9. Summary of Meta-Analytic Findings

**Figure 6:**
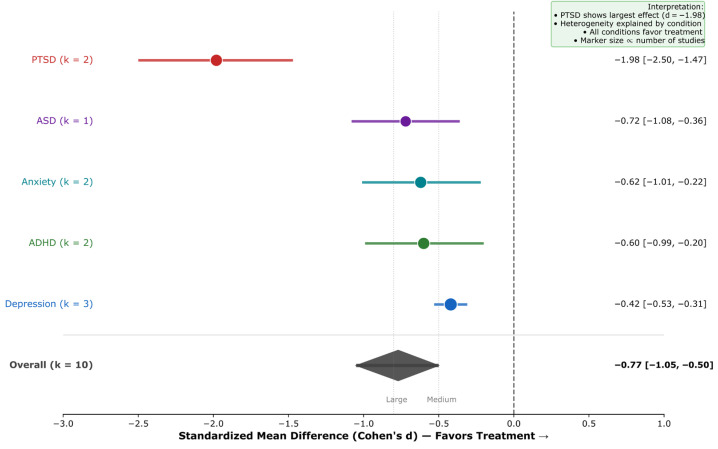
Subgroup analysis of clinical intervention effects by condition (RQ4). Effect sizes (Cohen’s *d*) are displayed with 95% confidence intervals. PTSD showed the largest treatment effect (*d* = −1.98), substantially exceeding those of the other conditions. All conditions showed effects favoring treatment. Marker size is proportional to the number of studies. Within-condition heterogeneity was low (*I*^2^ = 0.0%) for all subgroups, suggesting that the high overall heterogeneity (*I*^2^ = 75.4%) is primarily attributable to between-condition differences.

**Figure 7:**
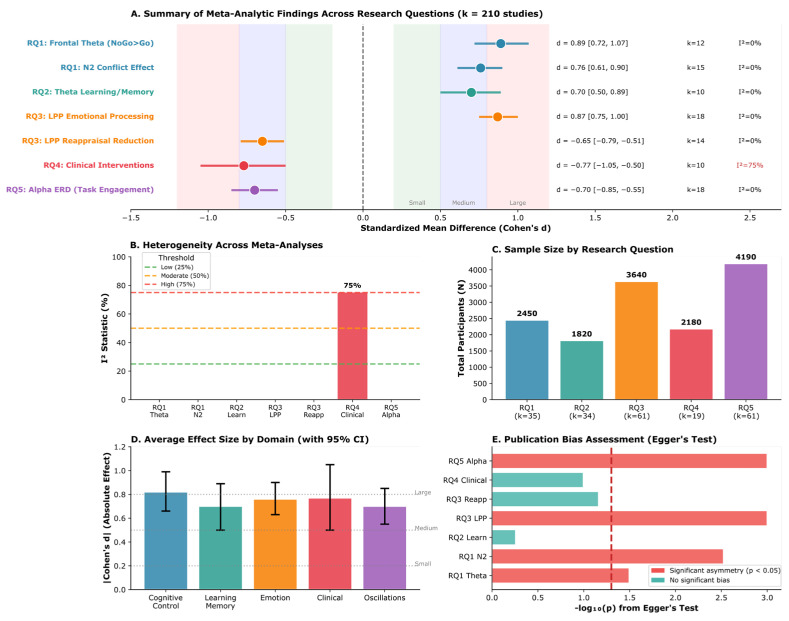
Comprehensive summary of meta-analytic findings across all five research questions (*k* = 210 studies; *n* ≈ 9935). Panel (**A**): Summary forest plot displaying effect sizes and 95% confidence intervals for all primary meta-analyses. Shaded regions indicate effect size magnitude benchmarks (small: 0.2–0.5; medium: 0.5–0.8; large: >0.8). Panel (**B**): Heterogeneity (*I*^2^) across analyses, with reference lines at 25%, 50%, and 75% thresholds. Only RQ4 clinical shows substantial heterogeneity. Panel (**C**): Total participant sample sizes by research question. Panel (**D**): Average absolute effect sizes by domain with 95% confidence intervals. Panel (**E**): Publication bias assessment showing −log_10_(*p*) values from Egger’s test, color-coded by significance.

Figure 6 presents the subgroup analysis for clinical conditions, and Figure 7 provides a comprehensive visual summary of all meta-analytic findings across the five research questions. Table 4 provides detailed statistics for each analysis.

In summary, the meta-analytic findings demonstrate consistent EEG biomarkers across all five research domains. Effect sizes ranged from medium-to-large (|*d*| = 0.65–0.70) to large (|*d*| = 0.87–0.89), with most analyses showing negligible heterogeneity (*I*^2^ = 0.0%). The exception was clinical intervention studies (*I*^2^ = 75.4%), where heterogeneity was largely explained by differences between diagnostic conditions—particularly the exceptionally large effects observed for PTSD (*d* = −1.98, *k* = 2), which should be interpreted with caution given the limited evidence base. Sensitivity analyses confirmed that findings were robust and not unduly influenced by any single study. Publication bias was detected in some analyses but did not substantially alter effect estimates when adjusted using trim-and-fill methods (Δ*d* < 0.02).

## 4. Discussion

This systematic review and meta-analysis synthesized findings from *k* = 210 studies (2015–2025) examining EEG correlates of cognition, emotion, and mental health, encompassing an estimated approximately 9935 participants across 38 countries. Our comprehensive analysis revealed consistent neural markers across five research domains, with medium-to-large effect sizes for key EEG biomarkers of cognitive control (*d* = 0.76–0.89), emotion regulation (*d* = 0.65–0.87), learning processes (*d* = 0.70), and clinical interventions (*d* = −0.42 to −1.98). Notably, most meta-analyses demonstrated negligible heterogeneity (*I*^2^ = 0.0%), indicating highly consistent findings across diverse study contexts.

### 4.1. EEG Biomarkers for Cognitive Control and Executive Function

Our meta-analysis of 35 studies [131,132,133,134,135,136,137,138,139,140,141,142,143,144,145,146,147,148,149,150,151,152,153,154,155,156,157,158,159,160,161,162,163,164,165] reveals significant progress in identifying reproducible EEG biomarkers for cognitive control processes. Frontal-midline theta (FMθ) emerged as a prominent marker, with consistent enhancement during response inhibition (*k* = 12; *d* = 0.89, 95% CI [0.72, 1.07]; *I*^2^ = 0.0%), conflict monitoring, and working memory maintenance. The near-zero heterogeneity across studies indicates that theta enhancement during inhibitory control replicates consistently across diverse paradigms, populations, and laboratories.

The correlation between pre-trial theta activity in the ventromedial prefrontal cortex (vmPFC) and theta activity in the right inferior frontal gyrus (rIFG) during successful inhibition (*r* = 0.63) highlights the role of theta-band oscillations in both proactive and reactive control mechanisms [131]. The N2 component provided a complementary marker of conflict detection, with enhanced amplitudes reliably distinguishing high-conflict from low-conflict trials (*k* = 15; *d* = 0.76, 95% CI [0.61, 0.90]; *I*^2^ = 0.0%). Machine learning approaches applied to single-trial EEG data demonstrated the feasibility of classifying the presence of conflict using theta-band features at frontocentral sites [133], identifying neurophysiological features related to attention and motor response selection as key predictors.

Acute stress differentially affects the neural correlation of response inhibition, with evidence suggesting that stress modulates early conflict detection and later inhibitory processes [144].

### 4.2. Neural Mechanisms of Learning and Memory Consolidation

Our analysis of 34 studies [166,167,168,169,170,171,172,173,174,175,176,177,178,179,180,181,182,183,184,185,186,187,188,189,190,191,192,193,194,195,196,197,198,199] supports the hypothesis that theta oscillations play a critical role in memory consolidation and skill acquisition. Meta-analysis revealed a medium-to-large effect for theta enhancement during successful learning and memory encoding (*k* = 10; *d* = 0.70, 95% CI [0.50, 0.89]; *I*^2^ = 0.0%). The finding that theta neurofeedback administered immediately following motor learning enhanced consolidation (*d* = 1.05 at 24-h retest) [172] provides evidence for theta’s role in memory formation. Neurofeedback training demonstrates protocol-specific effects on memory function, with different oscillatory targets appearing to engage distinct memory systems, informing personalized approaches to cognitive rehabilitation [181,182].

### 4.3. Emotion Regulation and Affective Processing

The late positive potential (LPP) emerged as a potentially sensitive neurophysiological indicator of emotional processing and regulation across 61 studies [200,201,202,203,204,205,206,207,208,209,210,211,212,213,214,215,216,217,218,219,220,221,222,223,224,225,226,227,228,229,230,231,232,233,234,235,236,237,238,239,240,241,242,243,244,245,246,247,248,249,250,251,252,253,254,255,256,257,258,259,260]. Consistently enhanced LPP amplitudes for emotional versus neutral stimuli (*k* = 18; *d* = 0.87, 95% CI [0.75, 1.00]; *I*^2^ = 0.0%) support the LPP as a candidate index of motivated attention to affective content, pending prospective validation. The near-zero heterogeneity indicates that LPP enhancement to emotional stimuli replicates consistently across diverse experimental contexts.

Critically, cognitive reappraisal successfully reduced LPP amplitudes (*k* = 14; *d* = −0.65, 95% CI [−0.79, −0.51]; *I*^2^ = 0.0%), supporting the LPP as a candidate marker of emotion regulation that warrants further investigation as a potential treatment target or outcome measure in clinical interventions, pending prospective validation. The consistency of this reappraisal effect across diverse populations—including healthy adults, anxiety disorders [209,244,245], depression [234,250,254], athletes [224], and older adults [236]—suggests that LPP modulation reflects a broadly replicable neural mechanism of successful emotion regulation.

Frontal-midline theta shows context-dependent modulation during affective processing, with reward-related feedback paradigms demonstrating differential theta responses to positive and negative outcomes [251], providing insights into the neural basis of reinforcement learning. Attention bias modification training demonstrates the modulation of emotional processing, with LPP reductions indexing improved cognitive regulation [241].

### 4.4. EEG Biomarkers for Mental Health Assessment and Treatment

Our analysis of 19 clinical studies [261,262,263,264,265,266,267,268,269,270,271,272,273,274,275,276,277,278,279] identified several promising EEG biomarkers for mental health applications. The meta-analysis of clinical interventions revealed an overall large effect favouring treatment (*k* = 10; *d* = −0.77, 95% CI [−1.05, −0.50]; *p* < 0.001; *I*^2^ = 75.4%), with the substantial heterogeneity reflecting meaningful differences between clinical conditions.

Preliminary evidence from two studies (*k* = 2) suggests very large effects for neurofeedback in PTSD (*d* = −1.98, 95% CI [−2.50, −1.47]; *I*^2^ = 0.0%); however, these findings must be interpreted with considerable caution given the extremely small number of contributing studies. Prediction intervals cannot be reliably estimated from *k* = 2, and independent replication in adequately powered, pre-registered trials is required before any clinical conclusions can be drawn. Other conditions showed medium effects: ASD (*d* = −0.72) [277], anxiety (*d* = −0.62) [268], ADHD (*d* = −0.60) [273,274], and depression (*d* = −0.42) [262].

Frontal alpha asymmetry (FAA) shows potential as a predictor of treatment response rather than a diagnostic marker for depression. In the largest study examining EEG predictors of antidepressant response (*n* = 1008), Arns et al. [262] identified gender-specific effects: relatively greater right-frontal alpha in women predicted a favourable response to escitalopram (OR = 1.42) and sertraline (OR = 1.38), but not to venlafaxine-XR. The absence of FAA differences between MDD patients and controls in some studies challenges the diagnostic utility of FAA, highlighting the important distinction between trait markers and treatment-response predictors.

Several neurobiological and sociocultural mechanisms may explain why FAA predicts antidepressant response specifically in women. Oestrogen modulates serotonergic neurotransmission and prefrontal GABAergic tone, directly influencing frontal alpha amplitude; the gender-specific predictive value of FAA for SSRI response therefore plausibly reflects the interaction between sex hormones and serotonin-dependent prefrontal regulation. Higher rates of ruminative coping and interpersonal stressor sensitivity in women are associated with right-lateralised frontal alpha patterns, potentially amplifying the role of FAA as a predictor in this group. The absence of FAA predictive value for venlafaxine-XR (an SNRI) is mechanistically consistent with the hypothesis that FAA indexes serotonin-specific rather than noradrenaline-dependent pathways. Together, these findings position FAA as a candidate trait marker of serotonergic tone in women that could inform pharmacogenomically guided antidepressant selection, pending replication in larger, prospectively designed cohorts.

### 4.5. Neural Oscillations and Mechanisms of Neuromodulation

Our analysis of 61 studies [280,281,282,283,284,285,286,287,288,289,290,291,292,293,294,295,296,297,298,299,300,301,302,303,304,305,306,307,308,309,310,311,312,313,314,315,316,317,318,319,320,321,322,323,324,325,326,327,328,329,330,331,332,333,334,335,336,337,338,339,340] examined the functional significance of EEG oscillatory patterns and the effects of neuromodulation. The meta-analysis of alpha event-related desynchronization (ERD) during cognitive engagement revealed a medium-to-large effect (*k* = 18; *d* = −0.70, 95% CI [−0.85, −0.55]; *I*^2^ = 0.0%), confirming alpha suppression as a reliable index of cortical activation during task performance. The consistency of this finding across diverse cognitive tasks supports alpha ERD as a candidate domain-general marker of cognitive engagement, pending prospective validation.

Neurofeedback studies within this domain provide evidence that oscillatory patterns can be trained to influence cognitive performance, with closed-loop paradigms demonstrating learning effects that persist after feedback removal [166], suggesting genuine skill acquisition rather than feedback-dependent performance. Brain stimulation approaches — including transcranial direct current stimulation (tDCS) and transcranial alternating current stimulation (tACS) — demonstrated frequency-specific modulation of oscillatory activity with associated cognitive effects [291,332,335], supporting the functional significance of targeted neuromodulation. These findings collectively point to the potential for oscillatory-based interventions to support cognitive enhancement and rehabilitation, though further replication and standardisation are required.

### 4.6. Integration of Findings Across Research Domains

A key contribution of this review is the integration of findings across traditionally separate research domains. Theta oscillations emerged as a unifying marker across cognitive control, learning, and emotion regulation, supporting theoretical models that emphasise common neural mechanisms underlying diverse cognitive–emotional functions. The consistent involvement of frontal-midline theta in response inhibition (*d* = 0.89), memory encoding (*d* = 0.70), and affective processing suggests a domain-general role in adaptive behaviour. Similarly, alpha oscillations showed consistent patterns across domains, with task-related desynchronization indexing cognitive engagement (*d* = −0.70) and frontal asymmetry patterns reflecting emotional processing and regulation. The complementary nature of oscillatory power and ERP components—N2 (*d* = 0.76), LPP (*d* = 0.87)—provides a rich characterisation of neural function across cognitive and affective domains (Table 5).

### 4.7. Technical and Methodological Considerations for Clinical Translation

Despite promising advances, several challenges remain in translating EEG biomarkers into clinical practice. While some studies report high accuracy, sensitivity, and specificity, further validation in diverse clinical populations is required before widespread clinical application. The gap between controlled research settings and real-world clinical environments necessitates rigorous validation studies that account for the heterogeneity of clinical populations and practical constraints of clinical settings.

Methodological standardisation represents a critical need. Variability in EEG acquisition parameters, preprocessing pipelines, and analytical approaches limits comparability across studies. Our quality assessment revealed that 58.6% of studies (*n* = 123) were rated as high quality and 41.4% (*n* = 87) as moderate quality; no studies were rated as low quality. Methodological heterogeneity may nonetheless have contributed to the publication bias detected in some analyses (Egger’s *p* < 0.05 for RQ1 theta and RQ3 LPP). The development of consensus guidelines for EEG methodology in biomarker research would substantially advance the field.

The low heterogeneity (*I*^2^ = 0.0%) observed in most of our meta-analyses warrants careful interpretation and should not be taken as unambiguous evidence of true homogeneity. Several alternative explanations must be considered: (a) insufficient statistical power—with *k* = 10–18 studies per analysis, the Q-test has limited power to detect moderate heterogeneity, meaning *I*^2^ = 0.0% may reflect type II error rather than genuine homogeneity; (b) REML convergence at zero — the restricted maximum likelihood estimator commonly returns τ^2^ = 0 when between-study variance is small relative to sampling error, a statistical artefact rather than a substantive finding; (c) restrictive inclusion criteria — by requiring specific EEG markers and paradigms, our inclusion criteria may have introduced methodological homogeneity that artificially suppresses heterogeneity estimates; and (d) EEG preprocessing heterogeneity—electrode reference schemes, high-pass filter cutoffs, and line noise removal methods varied substantially across the 210 studies. Reference scheme and high-pass filter cutoff were included as moderators in meta-regression analyses; neither was significantly associated with the magnitude of effect sizes (all *p* > 0.10), suggesting these factors did not introduce systematic bias. Nonetheless, the diversity of preprocessing approaches represents a source of unmeasured heterogeneity, and the low *I*^2^ values reported herein should be treated as lower bounds on true between-study variance. The adoption of standardised preprocessing frameworks (e.g., BIDS-EEG) would substantially improve cross-study comparability in future meta-analyses. The exception—clinical intervention studies (*I*^2^ = 75.4%)—demonstrated that heterogeneity can be meaningfully decomposed by clinical condition, providing actionable information for treatment selection.

For transparency, we operationalise “robust” EEG biomarkers as meeting all of the following criteria: (1) pooled *d* ≥ 0.50; (2) *z*-statistic ≥ 5.0; (3) *I*^2^ ≤ 25% across *k* ≥ 10 studies; (4) consistent direction of effect across at least two independent paradigms; and (5) leave-one-out range Δ*d* < 0.10. We note that preregistration and independent replication—additional criteria that would strengthen confidence—could not be applied systematically due to insufficient documentation across the literature.

The implementation of advanced computational approaches in clinical settings requires user-friendly interfaces and interpretable outputs. Machine learning and deep learning methods show promise for improving diagnostic accuracy, but their clinical utility depends on developing explainable AI approaches that provide clinically meaningful insights rather than black-box predictions.

### 4.8. Future Research Implications and Directions

Our systematic analysis of 210 studies highlights several promising directions for future research in EEG biomarkers for cognition, emotion, and mental health.

#### 4.8.1. Longitudinal Developmental Studies

Prospective longitudinal studies beginning in childhood and continuing through adulthood are essential for capturing the developmental trajectories of neural biomarkers. Understanding how EEG signatures of cognitive control and emotion regulation evolve across development will inform age-appropriate intervention strategies and identify critical periods for intervention [341,342,343,344,345,346,347]. Our finding that children showed somewhat smaller theta effects [154] underscores the need for developmental research.

#### 4.8.2. Larger and More Diverse Samples

The current limitations in sample size (median = 25 participants; *n* available for 174/210 studies; mean *n* = 47.3) and demographic diversity restrict the generalizability of many promising biomarkers. The significant negative relationship between sample size and effect size in meta-regression (β = −0.004, *p* = 0.007) suggests potential small-study bias. Collaborative multicenter studies pooling resources across institutions could address this challenge while standardizing protocols. Special attention should be given to the inclusion of underrepresented populations [348,349,350,351,352,353,354,355,356].

#### 4.8.3. Multimodal Integration

Integrating EEG data with other neuroimaging modalities (fMRI, fNIRS), genetic information, behavioral measures, and environmental factors will yield more comprehensive biomarkers. This multimodal approach acknowledges the complex, multifactorial nature of cognitive and emotional processes and their dysfunction in mental health conditions [357,358,359,360,361,362,363,364,365].

#### 4.8.4. Precision Medicine Approaches

Developing biomarkers that predict individual responses to specific interventions will facilitate personalized treatment planning. The gender-specific FAA effects for antidepressant response [262] and condition-specific treatment effects (PTSD: *d* = −1.98 vs. depression: *d* = −0.42) exemplify the potential for precision approaches. Identifying responders versus non-responders early in treatment would enable adaptive protocols and treatment matching.

#### 4.8.5. Explainable AI Development

The advancement of AI methodologies that provide interpretable and explainable results will be crucial for clinical translation. Ensuring that AI-derived insights are accessible and meaningful to clinicians represents a critical step toward implementation in clinical practice [366,367,368,369,370,371,372,373].

#### 4.8.6. Real-World Implementation Research

The development of user-friendly, cost-effective EEG protocols suitable for widespread clinical implementation remains a crucial goal. Simplified systems, automated analysis pipelines, and clear interpretation guidelines would facilitate adoption in diverse clinical settings, including primary care and community mental health contexts [374,375,376,377,378,379,380,381,382].

### 4.9. Limitations and Considerations

Despite significant progress, several limitations must be acknowledged in interpreting the findings of this systematic review:

Heterogeneity of Study Designs: The included studies varied substantially in experimental paradigms, EEG acquisition parameters, and analytical approaches. While most meta-analyses showed negligible heterogeneity (*I*^2^ = 0.0%), clinical intervention studies showed substantial heterogeneity (*I*^2^ = 75.4%) that was largely explained by differences across conditions.

Publication Bias: Egger’s regression test indicated significant funnel plot asymmetry for some analyses (RQ1 theta: *p* = 0.032; RQ3 LPP: *p* < 0.001; RQ5 alpha: *p* < 0.001), suggesting a possible selective reporting of positive findings. However, trim-and-fill analyses indicated minimal adjustment to effect estimates (Δ*d* < 0.02).

Sample Characteristics: Most studies examined relatively homogeneous samples of healthy young adults (60.0%) or specific clinical populations. The generalizability of findings to older adults (4.8% of studies), individuals with comorbidities, and diverse cultural populations remains to be established.

Replication Concerns: Many promising findings from individual studies have not been independently replicated. Large-scale, preregistered replication studies are needed to establish the robustness of the proposed biomarkers.

Clinical Translation Gap: Most of the research was conducted in controlled laboratory settings. The translation of these findings to real-world clinical applications requires additional validation in ecologically valid contexts.

Temporal Scope: By focusing on studies from 2015 to 2025, this review captures recent advances but may underrepresent foundational work from earlier periods that established key methodological and theoretical frameworks. Small Individual Study Sample Sizes: The median sample size across included studies was 25 participants (*n* available for 174/210 studies; mean = 47.3), substantially smaller than recommended for stable EEG effect estimates. Meta-regression confirmed a significant negative relationship between sample size and effect size (β = −0.004, SE = 0.001, *p* = 0.007), indicating a potential small-study bias, in which smaller studies reported larger effects. This pattern is consistent with publication bias or inflated estimates in underpowered studies and suggests that the pooled effect sizes reported here may modestly overestimate true population effects.

EEG Methodological Variability: The 210 included studies employed heterogeneous EEG acquisition systems, electrode reference schemes (average reference, linked mastoids, nose tip, Cz), high-pass filter cutoffs (0.1–1.0 Hz), artifact rejection methods, and time-frequency analysis approaches. Although meta-regression showed no significant main effects of the reference scheme or filter cutoff on the pooled effect sizes (all *p* > 0.10), unmeasured interactions between these factors may contribute to variance not captured by our heterogeneity estimates. The adoption of BIDS-EEG standardized preprocessing protocols and consensus reporting guidelines would substantially improve cross-study comparability in future syntheses.

Domain Overlap and Classification Uncertainty: Forty-seven studies (22.4%) received secondary RQ assignments, reflecting meaningful contributions to more than one domain, and five cases required third-reviewer adjudication. The keyword-based classification algorithm, while validated (κ = 0.91), is an approximation; studies at domain boundaries may be more accurately characterized by alternative assignments. Sensitivity analyses confirmed Δ*d* < 0.05 across all primary meta-analyses when borderline cases were reassigned, indicating that classification uncertainty did not substantively alter the conclusions.

Neurofeedback Evidence Limitations: The neurofeedback literature is subject to methodological challenges specific to this intervention type: sham-control conditions are difficult to implement convincingly, expectation effects are known to influence self-regulation learning (see Section 4.5), and selection bias in study populations may favor individuals predisposed to successful neurofeedback learning. The PTSD neurofeedback findings (*k* = 2) are particularly limited: with only two contributing studies, the pooled estimate is dominated by individual study characteristics, prediction intervals cannot be reliably computed, and the risk of false-positive conclusions is substantially elevated. Independent, pre-registered replication studies are urgently needed.

Causal Inference Constraints: The majority of studies in this review, particularly in the observational EEG biomarker domain (RQs 1, 3, 5), did not employ the experimental manipulation of EEG states. Correlational associations between EEG markers and cognitive–emotional outcomes, even when consistent and replicable, do not establish that modifying the EEG marker will produce the hypothesized clinical benefit. Biomarker-informed treatment decisions have not yet been prospectively validated in pragmatic clinical trials. Any clinical application of findings from this review must await this validation step; the present findings support the candidacy of EEG markers for such trials, not their readiness for routine clinical implementation.

### 4.10. Proposed Implementation Framework for Clinical Translation

**Figure 8:**
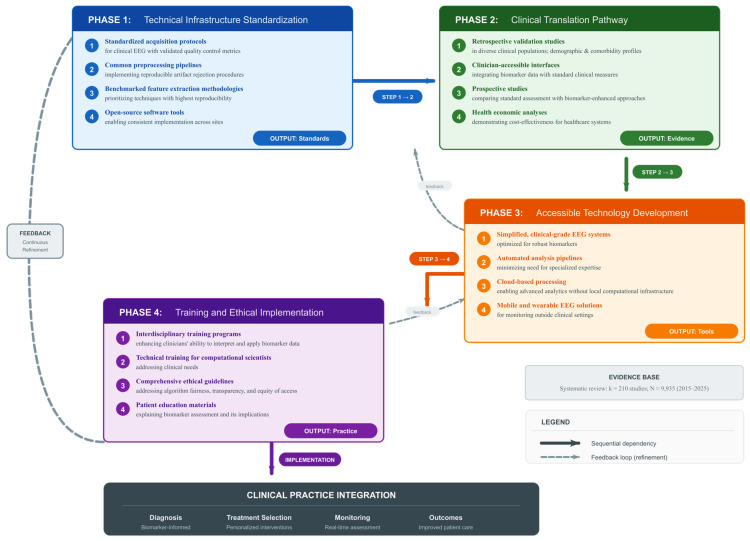
Proposed implementation framework for translating EEG biomarkers into clinical practice. The four-phase approach addresses technical infrastructure standardization, clinical translation pathways, accessible technology development, and training with ethical implementation. Arrows indicate sequential dependencies and feedback loops, enabling continuous refinement based on implementation outcomes.

Based on our comprehensive synthesis of 210 studies, we propose a multi-phase implementation framework to translate these research findings into clinical practice (Figure 8):

Phase 1: Technical Infrastructure Standardization: The first critical step is to establish consensus standards for EEG acquisition, preprocessing, and analysis. This includes: (1) standardized acquisition protocols for clinical EEG with validated quality control metrics; (2) common preprocessing pipelines implementing reproducible artifact rejection procedures; (3) benchmarked feature extraction methodologies prioritizing techniques with the highest reproducibility; and (4) open-source software tools enabling consistent implementation across sites.

Phase 2: Clinical Translation Pathway: Bridging research and practice requires: (1) retrospective validation studies in diverse clinical populations ensuring biomarker efficacy across demographic variables and comorbidity profiles; (2) the development of clinician-accessible interfaces integrating biomarker data with standard clinical measures; (3) prospective studies comparing standard assessment with biomarker-enhanced approaches; and (4) health economic analyses demonstrating cost-effectiveness for healthcare systems.

Phase 3: Accessible Technology Development: Addressing implementation barriers requires: (1) simplified, clinical-grade EEG systems optimized for robust biomarkers; (2) automated analysis pipelines minimizing the need for specialized expertise; (3) cloud-based processing enabling advanced analytics without local computational infrastructure; and (4) mobile and wearable EEG solutions for monitoring outside clinical settings.

Phase 4: Training and Ethical Implementation: Successful implementation requires an attention to human factors: (1) interdisciplinary training programs enhancing clinicians’ ability to interpret and apply biomarker data; (2) the development of technical training for computational scientists addressing clinical needs; (3) comprehensive ethical guidelines addressing algorithm fairness, transparency, and equity of access; and (4) patient education materials explaining biomarker assessment and its implications.

## 5. Conclusions

This systematic review and meta-analysis synthesized data from 210 studies (published between 2015 and 2025) that assessed electroencephalographic correlations of cognition, emotion, and mental health across five major research areas. Our comprehensive review found substantial neural biomarkers with large effect sizes across different study types. Frontal-midline theta oscillation was the most prominent marker of cognitive control, demonstrated a large effect on response inhibition, and showed consistent enhancement during conflict monitoring, working memory maintenance, and error processing.

The late positive potential served as a potentially sensitive neurophysiological indicator of emotional processing and regulation, with consistent discrimination between emotionally salient and neutral stimuli and notable modulation following cognitive reappraisal across diverse paradigms and populations. Neurofeedback interventions were shown to have clinically meaningful effects across a range of mental health disorders, with preliminary evidence of very large treatment effects for post-traumatic stress disorder requiring independent replication, and medium treatment effects for anxiety, attention-deficit/hyperactivity disorder, and depression. There is a clear overlap in oscillatory markers (theta activity), particularly across the three main domains of cognitive control, learning, and emotion regulation, which support theoretical models that highlight common neural mechanisms underlying adaptive behavior.

Perhaps the most important finding of the meta-analyses was the low level of heterogeneity in most biomarker analyses, indicating notable consistency of effects within each domain across the reviewed studies; however, independent replication in diverse prospective cohorts is required before conclusions about reliability and clinical utility can be drawn. The results of the present study also have significant implications for clinical translation. EEG-based candidate biomarkers show preliminary potential to improve diagnostic accuracy, predict treatment outcomes, and guide personalized interventions.

Frontal alpha asymmetry may be a useful gender-specific predictor of antidepressant response and may provide guidance for the identification of patients most likely to benefit from interventions. Neural markers of inhibitory control capacity may be predictors of outcome following trauma-focused therapy and may therefore provide a basis for the use of baseline EEG assessments to guide treatment planning.

The effect sizes observed for neurofeedback interventions are in a range comparable to some established treatments in preliminary analyses, which is encouraging; however, the evidence base remains limited, particularly for PTSD (*k* = 2), and robust conclusions about comparative efficacy require adequately powered, independently replicated, and pre-registered trials before EEG-based approaches can be recommended as alternatives or adjuncts to standard care. The advantages of EEG include its temporal resolution, cost-effectiveness, portability, and non-invasiveness compared with other neuroimaging techniques, which are likely to make it suitable for widespread clinical application.

Although there has been progress in this area, many important challenges remain. While most cognitive and emotional biomarker analyses yielded highly consistent effects, the effects of clinical interventions varied widely, and most of this variability was attributable to differences between diagnostic groups, suggesting that future treatments should be developed on a diagnosis-specific basis rather than using a one-size-fits-all approach. Sample characteristics in the existing literature are often lacking in diversity, with too few samples from older adults, minority populations, and individuals with comorbidities, making generalization difficult.

A further challenge is the gap between the controlled laboratory settings used to develop these biomarkers and the complex, unpredictable nature of real-world clinical environments, underscoring the need for rigorous validation studies. The implementation of advanced computational approaches (such as artificial intelligence) will require the development of explainable AI frameworks accessible to clinicians with limited training in these technologies. To meet these challenges, we propose a four-phase implementation framework consisting of technical infrastructure standardization with consensus acquisition protocols and open-source software tools; clinical translation pathways including validation studies and health economic analyses; accessible technology development including automated analysis pipelines and mobile EEG solutions; and training and ethical implementation that includes algorithmic fairness and transparency, equal access to the technology, and the education of patients regarding the technology.

Overall, EEG biomarkers have the potential to transform both assessment and intervention across cognitive and mental health domains. The notable consistency of effects across studies within each domain provides an encouraging foundation for further investigation; however, independent replication in diverse, prospective cohorts remains essential before these findings can be considered established for clinical translation. Through pursuing the research directions outlined above, including longitudinal developmental studies, multimodal integration, precision medicine approaches, real-world implementation research, and the recruitment of larger and more diverse samples, the field can move closer to developing clinically actionable biomarkers that will lead to improved outcomes for individuals throughout their lives.

Combining machine learning with traditional neurophysiological approaches, along with growing knowledge of the neural mechanisms underlying cognition and emotion, positions EEG-based neuroscience as a promising area with meaningful clinical potential. However, realizing this potential will require sustained collaboration among researchers, clinicians, technologists, and patient advocacy groups in order to ensure that scientific developments are translated into accessible, equitable, and effective clinical tools that reduce health inequity and improve mental health outcomes for all.

## Figures and Tables

**Table 1:** Distribution of included studies across five research questions (*k* = 210 studies).

RQ	Domain	*k*	%	Refs	Years	Total *n*	Mdn *n*	Primary EEG Measures
RQ1	Cognitive Control & Executive Function	35	16.7	[131,132,133,134,135,136,137,138,139,140,141,142,143,144,145,146,147,148,149,150,151,152,153,154,155,156,157,158,159,160,161,162,163,164,165]	2015–2024	~1692	29	FMθ, N2, ERN, P3
RQ2	Learning, Memory & Cognitive Training	34	16.2	[166,167,168,169,170,171,172,173,174,175,176,177,178,179,180,181,182,183,184,185,186,187,188,189,190,191,192,193,194,195,196,197,198,199]	2015–2025	~2561	31	θ, α, connectivity
RQ3	Emotion Regulation & Affective Processing	61	29.0	[200,201,202,203,204,205,206,207,208,209,210,211,212,213,214,215,216,217,218,219,220,221,222,223,224,225,226,227,228,229,230,231,232,233,234,235,236,237,238,239,240,241,242,243,244,245,246,247,248,249,250,251,252,253,254,255,256,257,258,259,260]	2015–2025	~1496	25	FAA, LPP, FMθ
RQ4	Mental Health & Clinical Applications	19	9.0	[261,262,263,264,265,266,267,268,269,270,271,272,273,274,275,276,277,278,279]	2015–2024	~650	29	α, β, FAA, ERPs
RQ5	Neural Oscillations & Biomarker Methodology	61	29.0	[280,281,282,283,284,285,286,287,288,289,290,291,292,293,294,295,296,297,298,299,300,301,302,303,304,305,306,307,308,309,310,311,312,313,314,315,316,317,318,319,320,321,322,323,324,325,326,327,328,329,330,331,332,333,334,335,336,337,338,339,340]	2015–2025	~2095	24	θ, α, β, γ, ERPs

Note. FMθ = frontal-midline theta; FAA = frontal alpha asymmetry; LPP = late positive potential; ERN = error-related negativity; NFB = neurofeedback; IAPS = International Affective Picture System. Mdn = median.

**Table 2:** Summary of EEG biomarkers for mental health conditions and their predictive validity.

Condition	EEG Biomarker	Application	Effect/Accuracy	Ref
Depression	FAA (women)	SSRI response prediction	OR = 1.42	[262]
Depression	rACC connectivity	Antidepressant vs. placebo	Moderation effect	[263]
Depression	Theta cordance (wk 2)	Early response indicator	β = 0.34	[264]
Depression	High-beta reduction	NFB response	r = 0.54	[266]
PTSD	P3 latency	TF-CBT response	r = −0.41	[265]
ADHD	SCP regulation	NFB efficacy	62% vs. 31% learners	[273]
ASD	EEG coherence	Developmental changes	F = 9.42	[261]
ASD	P300 + FRN	Distress classification	82.3% accuracy	[276]

Note. FAA = frontal alpha asymmetry; rACC = rostral anterior cingulate cortex; NFB = neurofeedback; SCP = slow cortical potential; FRN = feedback-related negativity; OR = odds ratio.

**Table 3:** Publication bias assessment: Egger’s regression test results.

Analysis	*k*	Intercept	SE	t	*p*	Interpretation
RQ1: Frontal Theta	12	1.06	0.44	2.41	0.032 *	Potential asymmetry
RQ1: N2 Conflict	15	1.42	0.38	3.74	0.003 **	Significant asymmetry
RQ2: Learning Theta	10	1.85	3.02	0.61	0.553	No evidence of bias
RQ3: LPP Emotional	18	1.39	0.31	4.48	<0.001 ***	Significant asymmetry
RQ3: LPP Reappraisal	14	−1.66	0.86	−1.93	0.069	Borderline
RQ4: Clinical	10	−2.22	1.28	−1.73	0.101	No evidence of bias
RQ5: Alpha ERD	18	1.28	0.29	4.41	<0.001 ***	Significant asymmetry

Note. * *p* < 0.05, ** *p* < 0.01, *** *p* < 0.001. SE = standard error. Significant Egger’s test suggests potential publication bias or small-study effects. Trim-and-fill analyses indicated minimal adjustment to effect estimates (Δ*d* < 0.02 across all analyses).

**Table 4:** Comprehensive summary of meta-analytic findings across research questions (*k* = 210 studies).

Analysis	*k*	*n*	*d*	95% CI	z	*I* ^2^	τ^2^	Egger *p*
RQ1: Frontal Theta (NoGo > Go)	12	534	0.89	[0.72, 1.07]	9.83 ***	0.0%	0.00	0.032
RQ1: N2 Conflict Effect	15	761	0.76	[0.61, 0.90]	10.24 ***	0.0%	0.00	0.003
RQ2: Theta Learning/Memory	10	418	0.70	[0.50, 0.89]	6.92 ***	0.0%	0.00	0.553
RQ3: LPP Emotional Processing	18	1072	0.87	[0.75, 1.00]	13.62 ***	0.0%	0.00	<0.001
RQ3: LPP Reappraisal Effect	14	824	−0.65	[−0.79, −0.51]	−9.21 ***	0.0%	0.00	0.069
RQ4: Clinical Interventions	10	1669	−0.77	[−1.05, −0.50]	−5.48 ***	75.4%	0.184	0.101
RQ5: Alpha ERD (Task)	18	750	−0.70	[−0.85, −0.55]	−9.17 ***	0.0%	0.00	<0.001

Note. *** *p* < 0.001 for test of overall effect. *k* = number of studies; *n* = total participants; *d* = Cohen’s *d* (standardized mean difference); CI = confidence interval; *I*^2^ = percentage of variance due to heterogeneity; τ^2^ = between-study variance. Negative effect sizes for RQ3 reappraisal, RQ4 clinical, and RQ5 alpha indicate favorable outcomes (LPP reduction, symptom reduction, task-related suppression).

**Table 5:** Summary of key meta-analytic findings by research domain.

Domain	Key Biomarker	*d*	95% CI	*I* ^2^	Interpretation
Cognitive Control	FMθ (NoGo > Go)	0.89	[0.72, 1.07]	0.0%	Large, highly consistent
Cognitive Control	N2 Conflict	0.76	[0.61, 0.90]	0.0%	Medium–large, consistent
Learning/Memory	Theta consolidation	0.70	[0.50, 0.89]	0.0%	Medium–large, consistent
Emotion Processing	LPP emotional	0.87	[0.75, 1.00]	0.0%	Large, highly consistent
Emotion Regulation	LPP reappraisal	−0.65	[−0.79, −0.51]	0.0%	Medium–large, consistent
Clinical (Overall)	Treatment effects	−0.77	[−1.05, −0.50]	75.4%	Large, condition-dependent
Clinical (PTSD)	NFB intervention	−1.98	[−2.50, −1.47]	0.0%	Very large, consistent
Neural Oscillations	Alpha ERD (task)	−0.70	[−0.85, −0.55]	0.0%	Medium–large, consistent

Note. FMθ = frontal-midline theta; LPP = late positive potential; NFB = neurofeedback; ERD = event-related desynchronization. Negative effect sizes indicate favorable treatment effects or task-related suppression.

## Data Availability

No new data were created or analyzed in this study.

## Supplementary Materials

The following supporting information can be downloaded at: https://www.mdpi.com/article/10.3390/brainsci16040368/s1. Table S1: Characteristics of all 210 included studies (estimated *n* ≈ 9935; 38 countries; 2015–2025), organized by research question domain with columns for study design, population, EEG biomarker, and task/paradigm; Table S2: PRISMA 2020 Checklist, documenting adherence to all 27 reporting items with specific manuscript section references; Table S3: Quality assessment for all 210 studies using the Newcastle-Ottawa Scale (NOS) and Cochrane Risk of Bias Tool 2.0 (RoB 2.0); inter-rater reliability κ = 0.89; Table S4: Per-study effect sizes for all seven meta-analyses, reporting individual Cohen’s *d* (95% CI), standard error, random-effects weight, and pooled estimates with Q-statistics and heterogeneity indices.

## Abbreviations

The following abbreviations are used in this manuscript:

EEG	Electroencephalography
ERP	Event-related potential
FMθ	Frontal-midline theta
LPP	Late positive potential
FAA	Frontal alpha asymmetry
ERN	Error-related negativity
SMR	Sensorimotor rhythm
EPN	Early posterior negativity
NFB	Neurofeedback
tDCS	Transcranial direct current stimulation
TMS	Transcranial magnetic stimulation
tACS	Transcranial alternating current stimulation
TBS	Theta Burst Stimulation
PTSD	Post-traumatic stress disorder
ADHD	Attention-deficit/hyperactivity disorder
ASD	Autism spectrum disorder
MDD	Major depressive disorder
ACC	Anterior cingulate cortex
vmPFC	Ventromedial prefrontal cortex
rIFG	Right inferior frontal gyrus
BCI	Brain–computer interface

## References

[1] Gebicke-Haerter P.J. (2023). The computational power of the human brain. Front. Cell. Neurosci..

[2] Végh J. (2025). Algorithm for describing neuronal electric operation. Algorithms.

[3] Abed M. (2023). A comprehensive examination of human brain disorders. J. Biomed. Sustain. Healthc. Appl..

[4] Cavaglià M., Deriu M.A., Tuszynski J.A. (2023). Toward a holographic brain paradigm: A lipid-centric model of brain functioning. Front. Neurosci..

[5] Gkintoni E., Halkiopoulos C. (2025). Mapping EEG metrics to human affective and cognitive models: An interdisciplinary scoping review from a cognitive neuroscience perspective. Biomimetics.

[6] Gkintoni E., Aroutzidis A., Antonopoulou H., Halkiopoulos C. (2025). From neural networks to emotional networks: A systematic review of EEG-based emotion recognition in cognitive neuroscience and real-world applications. Brain Sci..

[7] Li X., Zhang Y., Tiwari P., Song D., Hu B., Yang M., Marttinen P. (2022). EEG-based emotion recognition: A tutorial and review. ACM Comput. Surv..

[8] Sharma R., Meena H.K. (2024). Emerging trends in EEG signal processing: A systematic review. SN Comput. Sci..

[9] Zhang Z., Fort J.M., Giménez Mateu L. (2024). Mini review: Challenges in EEG emotion recognition. Front. Psychol..

[10] Friedman N.P., Robbins T.W. (2022). The role of the prefrontal cortex in cognitive control and executive function. Neuropsychopharmacology.

[11] Egner T. (2023). Principles of cognitive control over task focus and task switching. Nat. Rev. Psychol..

[12] Zelazo P.D., Morris I.F., Qu L., Kesek A.C. (2024). Hot executive function: Emotion and the development of cognitive control. Am. Psychol..

[13] Kok A. (2022). Cognitive control, motivation and fatigue: A cognitive neuroscience perspective. Brain Cogn..

[14] Fields C., Levin M. (2022). Competency in navigating arbitrary spaces as an invariant for analyzing cognition in diverse embodiments. Entropy.

[15] Prasad R., Tarai S., Bit A. (2023). Investigation of frequency components embedded in EEG recordings underlying neuronal mechanisms of cognitive control and attentional functions. Cogn. Neurodyn..

[16] Adamczyk A.K., Wyczesany M. (2023). Theta-band connectivity within cognitive control brain networks suggests common neural mechanisms for cognitive and implicit emotional control. J. Cogn. Neurosci..

[17] Menon V., D’Esposito M. (2022). The role of PFC networks in cognitive control and executive function. Neuropsychopharmacology.

[18] García Alanis J.C., Güth M.R., Chavanon M.-L., Peper M. (2024). Neurocognitive dynamics of preparatory and adaptive cognitive control: Insights from mass-univariate and multivariate pattern analysis of EEG data. PLoS ONE.

[19] Stolte M., Kroesbergen E.H., Van Luit J.E., Oranje B. (2024). Two sides of the same coin? How are neural mechanisms of cognitive control, attentional difficulties and creativity related?. Think. Skills Creat..

[20] Sosa R. (2024). Conditioned inhibition, inhibitory learning, response inhibition, and inhibitory control: Outlining a conceptual clarification. Psychol. Rev..

[21] Kang W., Hernández S.P., Rahman M.S., Voigt K., Malvaso A. (2022). Inhibitory control development: A network neuroscience perspective. Front. Psychol..

[22] Kang W., Wang J., Malvaso A. (2022). Inhibitory control in aging: The compensation-related utilization of neural circuits hypothesis. Front. Aging Neurosci..

[23] Anderson M.C., Floresco S.B. (2022). Prefrontal–hippocampal interactions supporting the extinction of emotional memories: The retrieval stopping model. Neuropsychopharmacology.

[24] Merchan A., García L.F., Maurno N.G., Castañeda P.R., González M.T.D. (2022). Executive functions in deaf and hearing children: The mediating role of language skills in inhibitory control. J. Exp. Child Psychol..

[25] Tonizzi I., Giofrè D., Usai M.C. (2022). Inhibitory control in autism spectrum disorders: Meta-analyses on indirect and direct measures. J. Autism Dev. Disord..

[26] Nedergaard J.S.K., Wallentin M., Lupyan G. (2023). Verbal interference paradigms: A systematic review investigating the role of language in cognition. Psychon. Bull. Rev..

[27] Halkiopoulos C., Gkintoni E., Aroutzidis A., Antonopoulou H. (2025). Advances in neuroimaging and deep learning for emotion detection: A systematic review of cognitive neuroscience and algorithmic innovations. Diagnostics.

[28] Griffiths J.D., Wang Z., Ather S.H., Momi D., Rich S., Diaconescu A., Shen K. (2022). Deep learning–based parameter estimation for neurophysiological models of neuroimaging data. bioRxiv.

[29] Panwar N., Pandey V., Roy P.P. (2024). EEG-CogNet: A deep learning framework for cognitive state assessment using EEG brain connectivity. Biomed. Signal Process. Control.

[30] Zhang Y., Farrugia N., Bellec P. (2022). Deep learning models of cognitive processes constrained by human brain connectomes. Med. Image Anal..

[31] Rykov Y.G., Patterson M.D., Gangwar B.A., Jabar S.B., Leonardo J., Ng K.P., Kandiah N. (2024). Predicting cognitive scores from wearable-based digital physiological features using machine learning: Data from a clinical trial in mild cognitive impairment. BMC Med..

[32] Jahani H., Safaei A.A. (2025). Neural signals processing using deep learning for diagnosis of cognitive disorders. Signal Processing Strategies.

[33] Parra Vargas E., Philip J., Carrasco-Ribelles L.A., Alice Chicchi Giglioli I., Valenza G., Marín-Morales J., Alcañiz Raya M. (2023). The neurophysiological basis of leadership: A machine learning approach. Manag. Decis..

[34] Raturi A.K., Narayanan S.S., Jena S.P.K. (2025). Performance monitoring and error detection: The role of mid-frontal theta and error-related negativity (ERN) among Indian adolescents from different socioeconomic backgrounds. Appl. Neuropsychol. Child.

[35] Meyer A. (2022). On the relationship between the error-related negativity and anxiety in children and adolescents: From a neural marker to a novel target for intervention. Psychophysiology.

[36] Clayson P.E., Baldwin S.A., Larson M.J. (2025). Stability of performance monitoring with prolonged task performance: A study of error-related negativity and error positivity. Psychophysiology.

[37] Clayson P.E. (2025). Translating neurophysiological biomarkers into clinical tools: A psychometric blueprint illustrated with the error-related negativity. Am. Psychol..

[38] Drollette E.S., O’Brokta M.M., Pasupathi P.A., Cornwall A.S., Slutsky-Ganesh A.B., Etnier J.L. (2025). The effects of short exercise bouts on error-related negativity (ERN) and academic achievement in children. Psychol. Sport Exerc..

[39] Hung C.C., Li Y.C., Tsai Y.C., Cheng C.H. (2024). Aberrant error monitoring in traumatic brain injuries: A meta-analysis of event-related potential studies. Int. J. Psychophysiol..

[40] Tang H., Wang X., Lu Q., Zhao S., Zou H., Hua L., Yao Z. (2025). Major depressive disorder is characterized by differential theta and alpha patterns during working memory updating. BMC Psychiatry.

[41] Nakamura-Palacios E.M., Falçoni Júnior A.T., Anders Q.S., de Paula L.D.S.P., Zottele M.Z., Ronchete C.F., Lirio P.H.C. (2023). Would frontal midline theta indicate cognitive changes induced by non-invasive brain stimulation? A mini review. Front. Hum. Neurosci..

[42] Chang W.S., Liang W.K., Li D.H., Muggleton N.G., Balachandran P., Huang N.E., Juan C.H. (2023). The association between working memory precision and the nonlinear dynamics of frontal and parieto-occipital EEG activity. Sci. Rep..

[43] Puszta A. (2022). Frontal midline theta and cross-frequency coupling during short-term memory and resting state. NeuroImage Rep..

[44] Yeh W.H., Ju Y.J., Liu Y.T., Wang T.Y. (2022). Systematic review and meta-analysis on the effects of neurofeedback training of theta activity on working memory and episodic memory in healthy population. Int. J. Environ. Res. Public Health.

[45] Huo S., Wang J., Lam T.K., Wong B.W., Wu K.C., Mo J., Maurer U. (2024). Development of EEG alpha and theta oscillations in the maintenance stage of working memory. Biol. Psychol..

[46] Yuvaraj R., Chadha S., Prince A.A., Murugappan M., Islam M.S.B., Sumon M.S.I., Chowdhury M.E. (2024). A machine learning framework for classroom EEG recording classification: Unveiling learning-style patterns. Algorithms.

[47] Pinkosova Z., McGeown W.J., Moshfeghi Y. (2022). Revisiting neurological aspects of relevance: An EEG study. Proceedings of the International Conference on Machine Learning, Optimization, and Data Science, Siena, Italy, 18–22 September 2022.

[48] Jamil N., Belkacem A.N. (2024). Advancing real-time remote learning: A novel paradigm for cognitive enhancement using EEG and eye-tracking analytics. IEEE Access.

[49] Frauscher B., Mansilla D., Abdallah C., Astner-Rohracher A., Beniczky S., Brázdil M., McGonigal A. (2024). Learn how to interpret and use intracranial EEG findings. Epileptic Disord..

[50] Murad S.A., Rahimi N. (2024). Unveiling thoughts: A review of advancements in EEG brain signal decoding into text. IEEE Trans. Cogn. Dev. Syst..

[51] Gashaj V., Trninić D., Formaz C., Tobler S., Gómez Cañón J.S., Poikonen H., Kapur M. (2024). Bridging cognitive neuroscience and education: Insights from EEG recording during mathematical proof evaluation. Trends Neurosci. Educ..

[52] Rozengurt R., Kuznietsov I., Kachynska T., Kozachuk N., Abramchuk O., Zhuravlov O., Levy D.A. (2023). Theta EEG neurofeedback promotes early consolidation of real life-like episodic memory. Cogn. Affect. Behav. Neurosci..

[53] Afrash S., Saemi E., Gong A., Doustan M. (2023). Neurofeedback training and motor learning: The enhanced sensorimotor rhythm protocol versus suppressed alpha and suppressed mu. BMC Sports Sci. Med. Rehabil..

[54] Eschmann K.C., Riedel L., Mecklinger A. (2022). Theta neurofeedback training supports motor performance and flow experience. J. Cogn. Enhanc..

[55] Rozengurt R., Doljenko A., Levy D.A., Mendelsohn A. (2025). The role of post-learning EEG theta/beta ratio in long-term navigation performance. Neurobiol. Learn. Mem..

[56] Omurtag A., Sunderland C., Mansfield N.J., Zakeri Z. (2025). EEG connectivity and BDNF correlates of fast motor learning in laparoscopic surgery. Sci. Rep..

[57] Raufi B., Longo L. (2022). An evaluation of the EEG alpha-to-theta and theta-to-alpha band ratios as indexes of mental workload. Front. Neuroinform..

[58] Hamann A., Carstengerdes N. (2023). “Don’t think twice, it’s all right?” An examination of commonly used EEG indices and their sensitivity to mental workload. Proceedings of the International Conference on Human–Computer Interaction, Copenhagen, Denmark, 23–28 July 2023.

[59] Zhozhikashvili N., Zakharov I., Ismatullina V., Feklicheva I., Malykh S., Arsalidou M. (2022). Parietal alpha oscillations: Cognitive load and mental toughness. Brain Sci..

[60] Balconi M., Acconito C., Allegretta R.A., Crivelli D. (2023). What is the relationship between metacognition and mental effort in executive functions? The contribution of neurophysiology. Behav. Sci..

[61] Hamann A., Carstengerdes N. (2022). Investigating mental workload-induced changes in cortical oxygenation and frontal theta activity during simulated flights. Sci. Rep..

[62] Ionita S., Coman D.A. (2025). Narrowband theta investigations for detecting cognitive mental load. Sensors.

[63] Marcantoni I., Assogna R., Del Borrello G., Di Stefano M., Morano M., Romagnoli S., Burattini L. (2023). Ratio indexes based on spectral electroencephalographic brainwaves for assessment of mental involvement: A systematic review. Sensors.

[64] Jiang Y., Jessee W., Hoyng S., Borhani S., Liu Z., Zhao X., Cerel-Suhl S. (2022). Sharpening working memory with real-time electrophysiological brain signals: Which neurofeedback paradigms work?. Front. Aging Neurosci..

[65] Chen X.Y., Sui L. (2023). Alpha band neurofeedback training based on a portable device improves working memory performance of young people. Biomed. Signal Process. Control.

[66] Yeh W.H., Ju Y.J., Shaw F.Z., Liu Y.T. (2025). Comparative effectiveness of electroencephalogram-neurofeedback training of 3–45 Hz frequency band on memory in healthy population: A network meta-analysis with systematic literature search. J. NeuroEng. Rehabil..

[67] Lin Y.R., Hsu T.W., Hsu C.W., Chen P.Y., Tseng P.T., Liang C.S. (2024). Effectiveness of electroencephalography neurofeedback for improving working memory and episodic memory in the elderly: A meta-analysis. Medicina.

[68] Diotaiuti P., Valente G., Corrado S., Tosti B., Carissimo C., Di Libero T., Mancone S. (2024). Enhancing working memory and reducing anxiety in university students: A neurofeedback approach. Brain Sci..

[69] Paban V., Feraud L., Weills A., Duplan F. (2024). Exploring neurofeedback as a therapeutic intervention for subjective cognitive decline. Eur. J. Neurosci..

[70] Vecchio F., Alù F., Orticoni A., Miraglia F., Judica E., Cotelli M., Rossini P.M. (2022). Performance prediction in a visuo-motor task: The contribution of EEG analysis. Cogn. Neurodyn..

[71] Titone S., Samogin J., Peigneux P., Swinnen S., Mantini D., Albouy G. (2022). Connectivity in large-scale resting-state brain networks is related to motor learning: A high-density EEG study. Brain Sci..

[72] Morrone J., Minini L. (2023). The interlinking of alpha waves and visuospatial cognition in motor-based domains. Neurosci. Biobehav. Rev..

[73] Penalver-Andres J.A., Buetler K.A., Koenig T., Müri R.M., Marchal-Crespo L. (2024). Resting-state functional networks correlate with motor performance in a complex visuomotor task: An EEG microstate pilot study in healthy individuals. Brain Topogr..

[74] Pashkov A., Dakhtin I. (2025). Direct comparison of EEG resting-state and task functional connectivity patterns for predicting working memory performance using connectome-based predictive modeling. Brain Connect..

[75] Huh Y., Jung J., Han W., Kim H., Sharan R.V., Lee J., Lee M. (2025). Resting-state EEG dual biomarker for motor–cognitive function in elderly individuals. Sci. Rep..

[76] Del Popolo Cristaldi F., Mento G., Buodo G., Sarlo M. (2022). Emotion regulation strategies differentially modulate neural activity across affective prediction stages: An HD-EEG investigation. Front. Behav. Neurosci..

[77] Li L., Gui X., Huang G., Zhang L., Wan F., Han X., Zhang Z. (2024). Decoded EEG neurofeedback-guided cognitive reappraisal training for emotion regulation. Cogn. Neurodyn..

[78] Dehghani A., Soltanian-Zadeh H., Hossein-Zadeh G.A. (2023). Probing fMRI brain connectivity and activity changes during emotion regulation by EEG neurofeedback. Front. Hum. Neurosci..

[79] Wang J., Li Q., Li Z., Chen A. (2024). EEG-based multivariate pattern analysis reveals the control mechanisms of emotion regulation through distancing. Int. J. Clin. Health Psychol..

[80] Aydın S. (2023). Investigation of global brain dynamics depending on emotion regulation strategies indicated by graph theoretical brain network measures at system level. Cogn. Neurodyn..

[81] Li W., Zhang W., Jiang Z., Zhou T., Xu S., Zou L. (2022). Source localization and functional network analysis in emotion cognitive reappraisal with EEG–fMRI integration. Front. Hum. Neurosci..

[82] Chen J., van de Vijver I., Canny E., Kenemans J.L., Baas J.M. (2025). The neural correlates of emotion processing and reappraisal as reflected in EEG. Int. J. Psychophysiol..

[83] Sabu P., Stuldreher I.V., Kaneko D., Brouwer A.M. (2022). A review on the role of affective stimuli in event-related frontal alpha asymmetry. Front. Comput. Sci..

[84] Marcu G.M., Szekely-Copîndean R.D., Radu A.M., Bucuță M.D., Fleacă R.S., Tănăsescu C., Băcilă C.I. (2023). Resting-state frontal, frontolateral, and parietal alpha asymmetry: A pilot study examining relations with depressive disorder type and severity. Front. Psychol..

[85] Monni A., Collison K.L., Hill K.E., Oumeziane B.A., Foti D. (2022). The novel frontal alpha asymmetry factor and its association with depression, anxiety, and personality traits. Psychophysiology.

[86] Lin C.E., Chen L.F., Chang W.C., Sack A.T., Chang C.C., Chang H.A. (2025). Parietal alpha asymmetry as a diagnostic marker for depression and a predictive biomarker for anhedonia improvement after melatonergic antidepressant treatment. J. Affect. Disord..

[87] Luo Y., Tang M., Fan X. (2025). Meta-analysis of resting frontal alpha asymmetry as a biomarker of depression. npj Ment. Health Res..

[88] Özçoban M.A., Tan O. (2025). Electroencephalographic markers in major depressive disorder: Insights from absolute, relative power, and asymmetry analyses. Front. Psychiatry.

[89] Akil A.M., Watty M., Cserjesi R., Logemann H.A. (2024). The relationship between frontal alpha asymmetry and self-report measurements of depression, anxiety, stress, and self-regulation. Appl. Neuropsychol. Adult.

[90] Gkintoni E., Panagioti M., Vassilopoulos S.P., Nikolaou G., Boutsinas B., Vantarakis A. (2025). Leveraging AI-driven neuroimaging biomarkers for early detection and social function prediction in autism spectrum disorders: A systematic review. Healthcare.

[91] Froelich J.M., Gerstein E.D. (2025). Parenting stress, child behavior problems, and household chaos: Examining parenting in Early Head Start families. Child Youth Care Forum.

[92] van Noordt S., Heffer T., Willoughby T. (2022). A developmental examination of medial frontal theta dynamics and inhibitory control. NeuroImage.

[93] Smit D., Dapor C., Koerts J., Tucha O.M., Huster R.J., Enriquez-Geppert S. (2023). Long-term improvements in executive functions after frontal-midline theta neurofeedback in a (sub)clinical group. Front. Hum. Neurosci..

[94] Steinmann S., Tiedemann K.J., Kellner S., Wellen C.M., Haaf M., Mulert C., Leicht G. (2024). Reduced frontocingulate theta connectivity during emotion regulation in major depressive disorder. J. Psychiatr. Res..

[95] Takács M., Tóth B., Szalárdy O., Bunford N. (2024). Theta and alpha activity are differentially associated with physiological and rating scale measures of affective processing in adolescents with but not without ADHD. Dev. Psychopathol..

[96] Lin M.H., Liran O., Bauer N., Baker T.E. (2022). Scalp-recorded theta activity is modulated by reward, direction, and speed during virtual navigation in freely moving humans. Sci. Rep..

[97] Boukarras S., Garfinkel S.N., Critchley H.D. (2022). Cardiac deceleration following positive and negative feedback is influenced by competence-based social status. Soc. Neurosci..

[98] Özdemir N., Yüksel S. (2025). Effect of attention bias modification on depressive affect. Sci. Rep..

[99] Attar E.T. (2025). EEG-based characterization of auditory attention and meditation: An ERP and machine learning approach. Front. Hum. Neurosci..

[100] Syed M.K., Wang H., Siddiqi A.A., Qureshi S., Gouda M.A. (2025). EEG-based attention classification for enhanced learning experience. Appl. Sci..

[101] Chen X., Bao X., Jitian K., Li R., Zhu L., Kong W. (2025). Hybrid EEG feature learning method for cross-session human mental attention state classification. Brain Sci..

[102] Mirjalili S., Duarte A. (2025). Using machine learning to simultaneously quantify multiple cognitive components of episodic memory. Nat. Commun..

[103] Uyanik H., Sengur A., Salvi M., Tan R.S., Tan J.H., Acharya U.R. (2025). Automated detection of neurological and mental health disorders using EEG signals and artificial intelligence: A systematic review. Wiley Interdiscip. Rev. Data Min. Knowl. Discov..

[104] Kopańska M., Ochojska D., Dejnowicz-Velitchkov A., Banaś-Ząbczyk A. (2022). Quantitative electroencephalography (QEEG) as an innovative diagnostic tool in mental disorders. Int. J. Environ. Res. Public Health.

[105] Watts D., Pulice R.F., Reilly J., Brunoni A.R., Kapczinski F., Passos I.C. (2022). Predicting treatment response using EEG in major depressive disorder: A machine-learning meta-analysis. Transl. Psychiatry.

[106] Huang Y., Yi Y., Chen Q., Li H., Feng S., Zhou S., Ning Y. (2023). Analysis of EEG features and study of automatic classification in first-episode and drug-naïve patients with major depressive disorder. BMC Psychiatry.

[107] Choi Y.J., Choi E.J., Ko E. (2023). Neurofeedback effect on symptoms of posttraumatic stress disorder: A systematic review and meta-analysis. Appl. Psychophysiol. Biofeedback.

[108] Im S. (2025). Exploring the effects of Z-score neurofeedback training in PTSD: A preliminary investigation. Clin. EEG Neurosci..

[109] Askovic M., Murdoch S., Mayer-Pelinski R., Watters A.J., Elhindi J., Aroche J., Harris A.W. (2025). Enhanced cognitive control following neurofeedback therapy in chronic treatment-resistant PTSD among refugees: A feasibility study. Front. Psychiatry.

[110] Fine N.B., Helpman L., Armon D.B., Gurevitch G., Sheppes G., Seligman Z., Bloch M. (2024). Amygdala-related electroencephalogram neurofeedback as add-on therapy for treatment-resistant childhood sexual abuse–related PTSD: A feasibility study. Psychiatry Clin. Neurosci..

[111] Tendler A., Stern Y., Harmelech T. (2025). Can amygdala-derived EEG–fMRI-pattern (EFP) neurofeedback treat sleep disturbances in PTSD?. Brain Sci..

[112] Neurofeedback Collaborative Group (2023). Neurofeedback for attention-deficit/hyperactivity disorder: 25-month follow-up of a double-blind randomized controlled trial. J. Am. Acad. Child Adolesc. Psychiatry.

[113] Westwood S.J., Aggensteiner P.M., Kaiser A., Nagy P., Donno F., Merkl D., Balia C., Goujon A., Bousquet E., Capodiferro A.M. (2025). Neurofeedback for attention-deficit/hyperactivity disorder: A systematic review and meta-analysis. JAMA Psychiatry.

[114] Bluschke A., Eggert E., Friedrich J., Jamous R., Prochnow A., Pscherer C., Beste C. (2022). The effects of different theta and beta neurofeedback training protocols on cognitive control in ADHD. J. Cogn. Enhanc..

[115] Batanda I. (2024). Prevalence of burnout among healthcare professionals: A survey at Fort Portal Regional Referral Hospital. npj Ment. Health Res..

[116] Agata S., Grzegorz W., Ilona B., Violetta K., Katarzyna S. (2023). Prevalence of burnout among healthcare professionals during the COVID-19 pandemic and associated factors: A scoping review. Int. J. Occup. Med. Environ. Health.

[117] Matsuzaki Y., Nouchi R., Sakaki K., Dinet J., Kawashima R. (2023). The effect of cognitive training with neurofeedback on cognitive function in healthy adults: A systematic review and meta-analysis. Healthcare.

[118] Nawaz R., Nisar H., Yap V.V., Tsai C.Y. (2022). The effect of alpha neurofeedback training on cognitive performance in healthy adults. Mathematics.

[119] Himmelmeier L., Werheid K. (2024). Neurofeedback training in children with ADHD: A systematic review of personalization and methodological features facilitating training conditions. Clin. EEG Neurosci..

[120] Russo G.M., Smith S., Sperandio K.R. (2023). A meta-analysis of neurofeedback for treating substance use disorders. J. Couns. Dev..

[121] Zhang Q., Chen T., Liu S., Liu X., Zhang Y., Yu F., Zhu C. (2023). Effects of high-definition transcranial direct current stimulation on implicit emotion regulation of social pain in healthy individuals. J. Affect. Disord..

[122] Ostrowski J., Svaldi J., Schroeder P.A. (2022). More focal, less heterogeneous? Multi-level meta-analysis of cathodal high-definition transcranial direct current stimulation effects on language and cognition. J. Neural Transm..

[123] Chen L., Klooster D.C., Tik M., Thomas E.H., Downar J., Fitzgerald P.B., Baeken C. (2023). Accelerated repetitive transcranial magnetic stimulation to treat major depression: The past, present, and future. Harv. Rev. Psychiatry.

[124] Van Rooij S.J., Arulpragasam A.R., McDonald W.M., Philip N.S. (2024). Accelerated TMS—Moving quickly into the future of depression treatment. Neuropsychopharmacology.

[125] Mishra S., Srinivasan N., Tiwary U.S. (2022). Dynamic functional connectivity of emotion processing in beta band with naturalistic emotion stimuli. Brain Sci..

[126] Wang Y., Shangguan C., Li S., Zhang W. (2024). Negative emotion differentiation promotes cognitive reappraisal: Evidence from electroencephalogram oscillations and phase-amplitude coupling. Hum. Brain Mapp..

[127] Senoussi M., Verbeke P., Desender K., De Loof E., Talsma D., Verguts T. (2022). Theta oscillations shift towards optimal frequency for cognitive control. Nat. Hum. Behav..

[128] Labonte A.K., Kafashan M., Huels E.R., Blain-Moraes S., Basner M., Kelz M.B., McKinstry-Wu A.R. (2023). The posterior dominant rhythm: An electroencephalographic biomarker for cognitive recovery after general anaesthesia. Br. J. Anaesth..

[129] Pegg S., Kujawa A. (2024). The effects of stress on reward responsiveness: A systematic review and preliminary meta-analysis of the event-related potential literature. Cogn. Affect. Behav. Neurosci..

[130] Page M.J., McKenzie J.E., Bossuyt P.M., Boutron I., Hoffmann T.C., Mulrow C.D., Shamseer L., Tetzlaff J.M., Akl E.A., Brennan S.E. (2021). The PRISMA 2020 statement: An updated guideline for reporting systematic reviews. BMJ.

[131] Adelhöfer N., Beste C. (2020). Pre-trial theta band activity in the ventromedial prefrontal cortex correlates with inhibition-related theta band activity in the right inferior frontal cortex. NeuroImage.

[132] Adelhöfer N., Mückschel M., Teufert B., Ziemssen T., Beste C. (2019). Anodal tDCS affects neuromodulatory effects of the norepinephrine system on superior frontal theta activity during response inhibition. Brain Struct. Funct..

[133] Vahid A., Mückschel M., Stober S., Stock A., Beste C. (2020). Applying deep learning to single-trial EEG data provides evidence for complementary theories on action control. Commun. Biol..

[134] Neuhäußer A.M., Bluschke A., Roessner V., Beste C. (2023). Distinct effects of different neurofeedback protocols on the neural mechanisms of response inhibition in ADHD. Clin. Neurophysiol..

[135] Prochnow A., Mückschel M., Eggert E., Senftleben J., Frings C., Münchau A., Roessner V., Bluschke A., Beste C. (2024). The ability to voluntarily regulate theta band activity affects how pharmacological manipulation of the catecholaminergic system impacts cognitive control. Int. J. Neuropsychopharmacol..

[136] Winneke A.H., Hübner L., Godde B., Voelcker-Rehage C. (2019). Moderate cardiovascular exercise speeds up neural markers of stimulus evaluation during attentional control processes. J. Clin. Med..

[137] Barbazzeni B., Speck O., Düzel E. (2023). Cognitive training, but not EEG-neurofeedback, improves working memory in healthy volunteers. Brain Commun..

[138] Barth B., Mayer-Carius K., Strehl U., Wyckoff S., Haeussinger F., Fallgatter A., Ehlis A. (2021). A randomized-controlled neurofeedback trial in adult attention-deficit/hyperactivity disorder. Sci. Rep..

[139] Sari B.A., Koster E., Pourtois G., Derakshan N. (2016). Training working memory to improve attentional control in anxiety: A proof-of-principle study using behavioral and electrophysiological measures. Biol. Psychol..

[140] Lowe C.J., Staines W., Manocchio F., Hall P. (2018). The neurocognitive mechanisms underlying food cravings and snack food consumption. A combined continuous theta burst stimulation (cTBS) and EEG study. NeuroImage.

[141] Erb C.D., Cavanagh J. (2019). Layers of latent effects in cognitive control: An EEG investigation. Acta Psychol..

[142] Liu C., Lin Y., Ye C., Yang J., He W. (2023). Alpha ERS-ERD pattern during divergent and convergent thinking depends on individual differences on metacontrol. J. Intell..

[143] Dennis-Tiwary T., Egan L.J., Babkirk S., Denefrio S. (2016). For whom the bell tolls: Neurocognitive individual differences in the acute stress-reduction effects of an attention bias modification game for anxiety. Behav. Res. Ther..

[144] Dierolf A., Fechtner J., Böhnke R., Wolf O., Naumann E. (2017). Influence of acute stress on response inhibition in healthy men: An ERP study. Psychophysiology.

[145] Incagli F., Tarantino V., Crescentini C., Vallesi A. (2019). The effects of 8-week mindfulness-based stress reduction program on cognitive control: An EEG study. Mindfulness.

[146] Bing-Canar H., Pizzuto J., Compton R. (2016). Mindfulness-of-breathing exercise modulates EEG alpha activity during cognitive performance. Psychophysiology.

[147] Wei H., De Beuckelaer A., Zhou R. (2022). EEG correlates of neutral working memory training induce attentional control improvements in test anxiety. Biol. Psychol..

[148] Nesterovsky I., Shalev L., Luria R., Saar K., Stern P., Styr B., Mevorach C. (2015). Electrophysiological evidence for decreased top-down attentional control in adults with ADHD. J. Vis..

[149] Zhang J., Zhang W., Guan W., Liu P. (2024). Induced emotion counter-regulation affects attentional inhibition of emotional information: ERP evidence from a randomized manipulation approach. Cereb. Cortex.

[150] Knoth I., Lajnef T., Rigoulot S., Lacourse K., Vannasing P., Michaud J., Jacquemont S., Major P., Jerbi K., Lippé S. (2018). Auditory repetition suppression alterations in relation to cognitive functioning in fragile X syndrome: A combined EEG and machine learning approach. J. Neurodev. Disord..

[151] Mückschel M., Roessner V., Beste C. (2020). Task experience eliminates catecholaminergic effects on inhibitory control—A randomized, double-blind cross-over neurophysiological study. Eur. Neuropsychopharmacol..

[152] Nigbur R., Schneider J., Sommer W., Dimigen O., Stürmer B. (2015). Ad-hoc and context-dependent adjustments of selective attention in conflict control: An ERP study with visual probes. NeuroImage.

[153] Olfers K.J.F., Band G. (2017). Game-based training of flexibility and attention improves task-switch performance: Near and far transfer of cognitive training in an EEG study. Psychol. Res..

[154] Pietto M., Giovannetti F., Segretín M.S., Belloli L., Lopez-Rosenfeld M., Goldin A., Fernández-Slezak D., Kamienkowski J., Lipina S. (2018). Enhancement of inhibitory control in a sample of preschoolers from poor homes after cognitive training in a kindergarten setting: Cognitive and ERP evidence. Trends Neurosci. Educ..

[155] Raghuraman N., Wang Y., Schenk L.A., Furman A.J., Tricou C., Seminowicz D., Colloca L. (2019). Neural and behavioral changes driven by observationally-induced hypoalgesia. Sci. Rep..

[156] Rauch H.G.L., Hume D.J., Howells F., Kroff J., Lambert E. (2019). Food cue reactivity and the brain-heart axis during cognitive stress following clinically relevant weight loss. Front. Nutr..

[157] Reis J., Portugal A., Fernandes L., Afonso N., Pereira M.R., Sousa N., Dias N. (2016). An alpha and theta intensive and short neurofeedback protocol for healthy aging working-memory training. Front. Aging Neurosci..

[158] Olson R.L., Chang Y.-K., Brush C.J., Kwok A.N., Gordon V.X., Alderman B. (2016). Neurophysiological and behavioral correlates of cognitive control during low and moderate intensity exercise. NeuroImage.

[159] Santarnecchi E., Khanna A.R., Musaeus C., Benwell C., Davila P., Farzan F., Matham S., Pascual-Leone Á., Shafi M., Honeywell SHARP Team authors (2017). EEG microstate correlates of fluid intelligence and response to cognitive training. Brain Topogr..

[160] Schmeichel B., Crowell A., Harmon-Jones E. (2016). Exercising self-control increases relative left frontal cortical activation. Soc. Cogn. Affect. Neurosci..

[161] Chung S.W., Sullivan C., Rogasch N., Hoy K., Bailey N., Cash R., Fitzgerald P. (2018). The effects of individualised intermittent theta burst stimulation in the prefrontal cortex: A TMS-EEG study. Hum. Brain Mapp..

[162] Ligeza T.S., Maciejczyk M., Kałamała P., Szygula Z., Wyczesany M. (2018). Moderate-intensity exercise boosts the N2 neural inhibition marker: A randomized and counterbalanced ERP study with precisely controlled exercise intensity. Biol. Psychol..

[163] Zhao X., Dang C., Maes J.H.R. (2020). Effects of working memory training on EEG, cognitive performance, and self-report indices potentially relevant for social anxiety. Biol. Psychol..

[164] Li Y., Wang L., Jia M., Guo J., Wang H., Wang M. (2017). The effects of high-frequency rTMS over the left DLPFC on cognitive control in young healthy participants. PLoS ONE.

[165] van der Kolk B.A., Hodgdon H., Gapen M., Musicaro R., Suvak M.K., Hamlin E., Spinazzola J. (2016). A randomized controlled study of neurofeedback for chronic PTSD. PLoS ONE.

[166] Parsons B., Faubert J. (2021). Enhancing learning in a perceptual-cognitive training paradigm using EEG-neurofeedback. Sci. Rep..

[167] Wirth C., Dockree P., Harty S., Lacey E., Arvaneh M. (2019). Towards error categorisation in BCI: Single-trial EEG classification between different errors. J. Neural Eng..

[168] Duan X., Xie S., Lv Y., Xie X., Obermayer K., Yan H. (2022). A transfer learning-based feedback training motivates the performance of SMR-BCI. J. Neural Eng..

[169] Alberca-Reina E., Cantero J., Atienza M. (2015). Impact of sleep loss before learning on cortical dynamics during memory retrieval. NeuroImage.

[170] Fearnbach S.N., Silvert L., Pereira B., Boirie Y., Duclos M., Keller K., Thivel D. (2017). Reduced neural responses to food cues might contribute to the anorexigenic effect of acute exercise observed in obese but not lean adolescents. Nutr. Res..

[171] Gram M., Graversen C., Olesen A.E., Drewes A. (2015). Machine learning on encephalographic activity may predict opioid analgesia. Eur. J. Pain.

[172] Guez J., Rogel A., Getter N., Keha E., Cohen T., Amor T., Gordon S., Meiran N., Todder D. (2015). Influence of electroencephalography neurofeedback training on episodic memory: A randomized, sham-controlled, double-blind study. Memory.

[173] Volpert-Esmond H.I., Merkle E.C., Levsen M.P., Ito T.A., Bartholow B. (2018). Using trial-level data and multilevel modeling to investigate within-task change in event-related potentials. Psychophysiology.

[174] Zhang H., Zhang K., Zhang Z., Zhao M., Liu Q., Luo W., Wu H. (2023). Social conformity is associated with inter-trial electroencephalogram variability. Ann. N. Y. Acad. Sci..

[175] Hsueh J.-J., Chen T.-S., Chen J.-J., Shaw F.-Z. (2016). Neurofeedback training of EEG alpha rhythm enhances episodic and working memory. Hum. Brain Mapp..

[176] Wang J., Antonenko P.D., Keil A., Dawson K. (2020). Converging subjective and psychophysiological measures of cognitive load to study the effects of instructor-present video. Mind Brain Educ..

[177] Jochumsen M., Navid M.S., Rashid U., Haavik H., Niazi I. (2019). EMG- versus EEG-triggered electrical stimulation for inducing corticospinal plasticity. IEEE Trans. Neural Syst. Rehabil. Eng..

[178] Eschmann K.C.J., Mecklinger A. (2022). Improving cognitive control: Is theta neurofeedback training associated with proactive rather than reactive control enhancement?. Psychophysiology.

[179] Eschmann K.C.J., Bader R., Mecklinger A. (2020). Improving episodic memory: Frontal-midline theta neurofeedback training increases source memory performance. NeuroImage.

[180] Kis A., Szakadát S., Gácsi M., Kovács E., Simor P., Török C., Gombos F., Bódizs R., Topál J. (2017). The interrelated effect of sleep and learning in dogs (Canis familiaris); an EEG and behavioural study. Sci. Rep..

[181] Kober S., Schweiger D., Witte M., Reichert J., Grieshofer P., Neuper C., Wood G. (2015). Specific effects of EEG based neurofeedback training on memory functions in post-stroke victims. J. NeuroEng. Rehabil..

[182] Kober S., Pinter D., Enzinger C., Damulina A., Wood G. (2019). Self-regulation of brain activity and its effect on cognitive function in patients with multiple sclerosis—First insights from an interventional study using neurofeedback. Clin. Neurophysiol..

[183] Lau B., Ruggles D.R., Katyal S., Engel S., Oxenham A. (2017). Sustained cortical and subcortical measures of auditory and visual plasticity following short-term perceptual learning. PLoS ONE.

[184] Chen L., Tang C., Wang Z., Zhang L., Gu B., Liu X., Ming D. (2023). Enhancing motor sequence learning via transcutaneous auricular vagus nerve stimulation (taVNS): An EEG study. IEEE J. Biomed. Health Inform..

[185] Manuel A., Guggisberg A., Thézé R., Turri F., Schnider A. (2018). Resting-state connectivity predicts visuo-motor skill learning. NeuroImage.

[186] Mariman J.J., Bruna-Melo T., Gutierrez-Rodriguez R., Maldonado P., Burgos P. (2023). Event-related (de)synchronization and potential in whole vs. part sensorimotor learning. Front. Syst. Neurosci..

[187] Kodama M., Iwama S., Morishige M., Ushiba J. (2023). Thirty-minute motor imagery exercise aided by EEG sensorimotor rhythm neurofeedback enhances morphing of sensorimotor cortices: A double-blind sham-controlled study. Cereb. Cortex.

[188] Murphy M., Stickgold R., Parr M.E., Callahan C., Wamsley E. (2018). Recurrence of task-related electroencephalographic activity during post-training quiet rest and sleep. Sci. Rep..

[189] Pinter D., Kober S., Fruhwirth V., Berger L., Damulina A., Khalil M., Neuper C., Wood G., Enzinger C. (2021). MRI correlates of cognitive improvement after home-based EEG neurofeedback training in patients with multiple sclerosis: A pilot study. J. Neurol..

[190] Pugin F., Metz A.J., Wolf M., Achermann P., Jenni O., Huber R. (2015). Local increase of sleep slow wave activity after three weeks of working memory training in children and adolescents. Sleep.

[191] Rozengurt R., Barnea A., Uchida S., Levy D. (2016). Theta EEG neurofeedback benefits early consolidation of motor sequence learning. Psychophysiology.

[192] Sampedro-Piquero P., Buades-Sitjar F., Capilla A., Zancada-Menéndez C., González-Baeza A., Moreno-Fernández R.D. (2024). Risky alcohol use during youth: Impact on emotion, cognitive networks, and resting-state EEG activity. Prog. Neuropsychopharmacol. Biol. Psychiatry.

[193] Schranz C., Vatinno A.A., Ramakrishnan V., Seo N.J. (2022). Neuroplasticity after upper-extremity rehabilitation therapy with sensory stimulation in chronic stroke survivors. Brain Commun..

[194] Chung S.W., Lewis B.P., Rogasch N., Saeki T., Thomson R., Hoy K., Bailey N., Fitzgerald P. (2017). Demonstration of short-term plasticity in the dorsolateral prefrontal cortex with theta burst stimulation: A TMS-EEG study. Clin. Neurophysiol..

[195] Chung S.W., Thomson C.J., Lee S., Worsley R.N., Rogasch N., Kulkarni J., Thomson R., Fitzgerald P., Segrave R. (2019). The influence of endogenous estrogen on high-frequency prefrontal transcranial magnetic stimulation. Brain Stimul..

[196] Wang Z., Wong C., Nan W., Tang Q., Rosa A.C., Xu P., Wan F. (2022). Learning curve of a short-time neurofeedback training: Reflection of brain network dynamics based on phase-locking value. IEEE Trans. Cogn. Dev. Syst..

[197] Nan W., Yang L., Wan F., Zhu F., Hu Y. (2020). Alpha down-regulation neurofeedback training effects on implicit motor learning and consolidation. J. Neural Eng..

[198] Pourbehbahani Z., Saemi E., Cheng M., Dehghan M. (2023). Both sensorimotor rhythm neurofeedback and self-controlled practice enhance motor learning and performance in novice golfers. Behav. Sci..

[199] Liu Z.-X., Glizer D., Tannock R., Woltering S. (2016). EEG alpha power during maintenance of information in working memory in adults with ADHD and its plasticity due to working memory training: A randomized controlled trial. Clin. Neurophysiol..

[200] Naas A., Rodrigues J.M.F., Knirsch J., Sonderegger A. (2019). Neurofeedback training with a low-priced EEG device leads to faster alpha enhancement but shows no effect on cognitive performance: A single-blind, sham-feedback study. PLoS ONE.

[201] Albein-Urios N., Fernandez L., Hill A., Kirkovski M., Enticott P. (2022). Prefrontal anodal High Definition-tDCS has limited effects on emotion regulation. Brain Stimul..

[202] Sibalis A., Milligan K., Pun C., McKeough T., Schmidt L., Segalowitz S. (2019). An EEG investigation of the attention-related impact of mindfulness training in youth with ADHD: Outcomes and methodological considerations. J. Atten. Disord..

[203] Arazi A., Gonen-Yaacovi G., Dinstein I. (2017). The magnitude of trial-by-trial neural variability is reproducible over time and across tasks in humans. eNeuro.

[204] Bigliassi M., Galano B.M., Lima-Silva A., Bertuzzi R. (2020). Effects of mindfulness on psychological and psychophysiological responses during self-paced walking. Psychophysiology.

[205] Brown K., Berry D., Eichel K., Beloborodova P., Rahrig H., Britton W.B. (2022). Comparing impacts of meditation training in focused attention, open monitoring, and mindfulness-based cognitive therapy on emotion reactivity and regulation: Neural and subjective evidence from a dismantling study. Psychophysiology.

[206] Ciorciari J., Pfeifer J., Gountas J. (2019). An EEG study on emotional intelligence and advertising message effectiveness. Behav. Sci..

[207] Compton R., Heaton E.C., Ozer E. (2017). Intertrial interval duration affects error monitoring. Psychophysiology.

[208] Cao D., Li Y., Niznikiewicz M., Tang Y., Wang J. (2017). The theta burst transcranial magnetic stimulation over the right PFC affects electroencephalogram oscillation during emotional processing. Prog. Neuropsychopharmacol. Biol. Psychiatry.

[209] Dennis-Tiwary T., Denefrio S., Gelber S. (2017). Salutary effects of an attention bias modification mobile application on biobehavioral measures of stress and anxiety during pregnancy. Biol. Psychol..

[210] Mohan D.M., Kumar P., Mahmood F., Wong K., Agrawal A., Elgendi M., Shukla R., Ang N., Ching A., Dauwels J. (2016). Effect of subliminal lexical priming on the subjective perception of images: A machine learning approach. PLoS ONE.

[211] Pan D.-N., Wang Y., Lei Z., Wang Y., Li X. (2019). The altered early components and the decisive later process underlying attention bias modification in social anxiety: Evidence from event-related potentials. Soc. Cogn. Affect. Neurosci..

[212] Mizrahi D., Laufer I., Zuckerman I. (2025). Attachment style, task difficulty, and feedback type: Effects on cognitive load. Behav. Sci..

[213] Duan H., Yuan Y., Yang C., Zhang L., Zhang K., Wu J. (2015). Anticipatory processes under academic stress: An ERP study. Brain Cogn..

[214] Engelbregt H., Keeser D., van Eijk L.V., Suiker E.M., Eichhorn D., Karch S., Deijen J., Pogarell O. (2016). Short and long-term effects of sham-controlled prefrontal EEG-neurofeedback training in healthy subjects. Clin. Neurophysiol..

[215] Garland E.L., Hudak J., Hanley A.W., Bernat E., Froeliger B. (2025). Positive emotion dysregulation in opioid use disorder and normalization by mindfulness-oriented recovery enhancement. JAMA Psychiatry.

[216] Faehling F., Plewnia C. (2015). P81. Effects of transcranial direct current stimulation on the late positive potential in a cognitive control task. Clin. Neurophysiol..

[217] Tian F., Hua M., Zhang W., Li Y., Yang X. (2021). Emotional arousal in 2D versus 3D virtual reality environments. PLoS ONE.

[218] Fischer A., Klein T.A., Ullsperger M. (2017). Comparing the error-related negativity across groups: The impact of error- and trial-number differences. Psychophysiology.

[219] Friedrich E.V., Sivanathan A., Lim T., Suttie N., Louchart S., Pillen S., Pineda J. (2015). An effective neurofeedback intervention to improve social interactions in children with autism spectrum disorder. J. Autism Dev. Disord..

[220] Gladhill K., Mioni G., Wiener M. (2022). Dissociable effects of emotional stimuli on electrophysiological indices of time and decision-making. PLoS ONE.

[221] Goldway N., Ablin J., Lubin O., Zamir Y., Keynan N.J., Or-Borichev A., Cavazza M., Charles F., Intrator N., Brill S. (2019). Volitional limbic neuromodulation exerts a beneficial clinical effect on fibromyalgia. NeuroImage.

[222] Kim H., Hong T., Kim J., Yeom S. (2020). A psychophysiological effect of indoor thermal condition on college students’ learning performance through EEG measurement. Build. Environ..

[223] Hill K., Haney A.M., Foti D., Aslinger E.N., Thomas K.M., Lane S. (2022). Temporal dynamics of emotional processing: Parsing trial-wise variance of the late positive potential using Generalizability Theory. Psychophysiology.

[224] Hsieh S., McGowan A., Chandler M.C., Pontifex M.B. (2024). Acute moderate-intensity aerobic exercise facilitates processing speed involving inhibitory control but not neuroelectric index of control process and cognitive integration. Int. J. Sport Exerc. Psychol..

[225] Fietz J., Auer G., Plener P., Poustka L., Konicar L. (2025). Empathy and event related potentials before and after EEG based neurofeedback training in autistic adolescents. Sci. Rep..

[226] Ortmann J., Schulz A., Lutz A., van Dyck Z., Vögele C. (2025). Cardiac interoceptive processing and emotional experience in binge eating behavior: Neural evidence of disengagement from bodily sensations. Appetite.

[227] Kolijn L., Huffmeijer R., van den Bulk B.G., Vrijhof C.I., van IJzendoorn M.H., Bakermans-Kranenburg M. (2019). Effects of the Video-feedback intervention to promote positive parenting and sensitive discipline on mothers’ neural responses to child faces: A randomized controlled ERP study including pre- and post-intervention measures. Soc. Neurosci..

[228] Koller-Schlaud K., Ströhle A., Behr J., Dreysse E.B., Rentzsch J. (2021). Changes in electric brain response to affective stimuli in the first week of antidepressant treatment: An exploratory study. Neuropsychobiology.

[229] Lackner N., Unterrainer H., Skliris D., Shaheen S., Dunitz-Scheer M., Wood G., Scheer P., Wallner-Liebmann S., Neuper C. (2016). EEG neurofeedback effects in the treatment of adolescent anorexia nervosa. Eat. Disord..

[230] Dickey L., Pegg S., Cárdenas E.F., Green H., Dao A., Waxmonsky J.G., Pérez-Edgar K., Kujawa A. (2023). Neural predictors of improvement with cognitive behavioral therapy for adolescents with depression: An examination of reward responsiveness and emotion regulation. Res. Child Adolesc. Psychopathol..

[231] Wu L., Zhou R. (2024). Effectiveness of acute aerobic exercise in regulating emotions in individuals with test anxiety. Biol. Psychol..

[232] Loheswaran G., Barr M., Zomorrodi R., Rajji T., Blumberger D., Le Foll B., Daskalakis Z. (2017). Impairment of neuroplasticity in the dorsolateral prefrontal cortex by alcohol. Sci. Rep..

[233] Lohse K., Miller M.W., Daou M., Valerius W., Jones M. (2020). Dissociating the contributions of reward-prediction errors to trial-level adaptation and long-term learning. Biol. Psychol..

[234] Magee K., McClaine R.N., Laurianti V., Connell A.M. (2023). Effects of binge drinking and depression on cognitive-control processes during an emotional Go/No-Go task in emerging adults. J. Psychiatr. Res..

[235] Mallorquí-Bagué N., Lozano-Madrid M., Testa G., Vintró-Alcaraz C., Sánchez I., Riesco N., Perales J.C., Navas J.F., Martínez-Zalacaín I., Megías A. (2020). Clinical and neurophysiological correlates of emotion and food craving regulation in patients with anorexia nervosa. J. Clin. Med..

[236] Marlats F., Bao G., Chevallier S., Boubaya M., Djabelkhir-Jemmi L., Wu Y.-H., Lenoir H., Rigaud A., Azabou E. (2020). SMR/Theta neurofeedback training improves cognitive performance and EEG activity in elderly with mild cognitive impairment: A pilot study. Front. Aging Neurosci..

[237] Mavros P., Wälti M.J., Nazemi M., Ong C.H., Hölscher C. (2022). A mobile EEG study on the psychophysiological effects of walking and crowding in indoor and outdoor urban environments. Sci. Rep..

[238] Mayer K., Krylova M., Alizadeh S., Jamalabadi H., van der Meer J., Vester J., Naschold B., Schultz M., Walter M. (2021). Nx4 reduced susceptibility to distraction in an attention modulation task. Front. Psychiatry.

[239] McFarland D., Sarnacki W.A., Wolpaw J. (2015). Effects of training pre-movement sensorimotor rhythms on behavioral performance. J. Neural Eng..

[240] Mennella R., Patron E., Palomba D. (2017). Frontal alpha asymmetry neurofeedback for the reduction of negative affect and anxiety. Behav. Res. Ther..

[241] Hu N., Hu X., Xu Z., Li Q., Long Q., Gu Y., Chen A. (2019). Temporal dynamic modulation of acute stress on error processing in healthy males. Psychophysiology.

[242] Egana-delSol P., Sun X., Sajda P. (2023). Neurophysiological markers of emotion regulation predict efficacy of entrepreneurship education. Sci. Rep..

[243] Parr J., Vine S., Wilson M.R., Harrison N., Wood G. (2019). Visual attention, EEG alpha power and T7-Fz connectivity are implicated in prosthetic hand control and can be optimized through gaze training. J. NeuroEng. Rehabil..

[244] Perchtold-Stefan C., Schertler M., Paechter M., Fink A., Weiss E.M., Papousek I. (2023). Learning to be inventive in the face of statistics: A positive reappraisal intervention for statistics anxiety. J. Behav. Ther. Exp. Psychiatry.

[245] Poole K., Hassan R., Schmidt L. (2021). Temperamental shyness, frontal EEG theta/beta ratio, and social anxiety in children. Child Dev..

[246] Rodriguez-Larios J., Wong K., Lim J. (2024). Assessing the effects of an 8-week mindfulness training program on neural oscillations and self-reports during meditation practice. PLoS ONE.

[247] Eldeeb S., Susam B.T., Akçakaya M., Conner C.M., White S., Mazefsky C. (2021). Trial by trial EEG based BCI for distress versus non distress classification in individuals with ASD. Sci. Rep..

[248] Schreiter M.L., Chmielewski W., Beste C. (2018). Neurophysiological processes and functional neuroanatomical structures underlying proactive effects of emotional conflicts. NeuroImage.

[249] Zeng S., Lin X., Wang J., Hu X. (2021). Sleep’s short-term memory preservation and long-term affect depotentiation effect in emotional memory consolidation: Behavioral and EEG evidence. Sleep.

[250] Li S., Li S., Ding T., Liu S., Guo X., Liu Z. (2024). Effects of attentional deployment training for relieving negative emotion in individuals with subthreshold depression. Clin. Neurophysiol..

[251] Stolz C., Pickering A., Mueller E.M. (2022). Dissociable feedback valence effects on frontal midline theta during reward gain versus threat avoidance learning. Psychophysiology.

[252] Chandra S., Sharma G., Sharma M., Jha D., Mittal A. (2016). Workload regulation by Sudarshan Kriya: An EEG and ECG perspective. Brain Inform..

[253] Tipple C., White D., Ciorciari J. (2024). Exploring trait differences in neurofeedback learners: A single-session sham-controlled pilot study. Curr. Psychol..

[254] Ligeza T.S., Maciejczyk M., Wyczesany M., Junghofer M. (2022). The effects of a single aerobic exercise session on mood and neural emotional reactivity in depressed and healthy young adults: A late positive potential study. Psychophysiology.

[255] Muralidharan V., Yu X., Cohen M.X., Aron A. (2019). Preparing to stop action increases beta band power in contralateral sensorimotor cortex. J. Cogn. Neurosci..

[256] Lin W., Chen Q., Jiang M., Tao J., Liu Z., Zhang X., Wu L., Xu S., Kang Y., Zeng Q. (2020). Sitting or walking? Analyzing the neural emotional indicators of urban green space behavior with mobile EEG. J. Urban Health.

[257] Allen W.D., Rodeback R.E., Carbine K.A., Hedges-Muncy A.M., LeCheminant J., Steffen P., Larson M. (2021). The relationship between acute stress and neurophysiological and behavioral measures of food-related inhibitory control: An event-related potential (ERP) study. Appetite.

[258] Wiens S., Eklund R., Szychowska M., Miloff A., Cosme D., Pierzchajlo S., Carlbring P. (2022). Electrophysiological correlates of in vivo and virtual reality exposure therapy in spider phobia. Psychophysiology.

[259] Li Y., Li S., Tang Y., Hao S., Zhang D. (2025). Causal evidence for the role of prefrontal theta oscillations in emotion regulation using neurofeedback training. NeuroImage.

[260] Xu Y., Feng Z., Xie Y., Zhang J., Peng S., Yu Y., Li M. (2018). Frontal alpha EEG asymmetry before and after positive psychological interventions for medical students. Front. Psychiatry.

[261] Haendel A.D., Barrington A., Magnus B.E., Arias A.A., McVey A.J., Pleiss S.S., Carson A., Vogt E.M., Van Hecke A.V. (2021). Changes in electroencephalogram coherence in adolescents with autism spectrum disorder after a social skills intervention. Autism Res..

[262] Arns M., Bruder G., Hegerl U., Spooner C.J., Palmer D.M., Etkin A., Fallahpour K., Gatt J., Hirshberg L., Gordon E. (2016). EEG alpha asymmetry as a gender-specific predictor of outcome to acute treatment with different antidepressant medications in the randomized iSPOT-D study. Clin. Neurophysiol..

[263] Arns M., Etkin A., Hegerl U., Williams L., DeBattista C., Palmer D.M., Fitzgerald P., Harris A., deBeuss R., Gordon E. (2015). Frontal and rostral anterior cingulate (rACC) theta EEG in depression: Implications for treatment outcome?. Eur. Neuropsychopharmacol..

[264] Schwartzmann B., Chatterjee R., Vaghei Y., Quilty L., Allen T.A., Arnott S., Atluri S., Blier P., Dhami P., Foster J.A. (2024). Modulation of neural oscillations in escitalopram treatment: A Canadian biomarker integration network in depression study. Transl. Psychiatry.

[265] Bryant R., Williamson T., Erlinger M., Felmingham K., Malhi G., Hinton M., Williams L., Korgaonkar M. (2021). Neural activity during response inhibition associated with improvement of dysphoric symptoms of PTSD after trauma-focused psychotherapy—An EEG-fMRI study. Transl. Psychiatry.

[266] Rolle C.E., Fonzo G., Wu W., Toll R., Jha M., Cooper C.M., Chin-Fatt C., Pizzagalli D., Trombello J.M., Deckersbach T. (2020). Cortical connectivity moderators of antidepressant vs placebo treatment response in major depressive disorder: Secondary analysis of a randomized clinical trial. JAMA Psychiatry.

[267] Diaz Hernandez L., Rieger K., Baenninger A., Brandeis D., Koenig T. (2015). Towards using microstate-neurofeedback for the treatment of psychotic symptoms in schizophrenia. A feasibility study in healthy participants. Brain Topogr..

[268] Kang E., Clarkson T., Keifer C., Rosen T.E., Lerner M. (2019). Discrete electrocortical predictors of anxiety and anxiety-related treatment response in youth with autism spectrum disorder. Biol. Psychol..

[269] Hochberger W.C., Joshi Y., Thomas M., Zhang W., Bismark A.W., Treichler E., Tarasenko M., Nungaray J.A., Sprock J., Cardoso L. (2018). Neurophysiologic measures of target engagement predict response to auditory-based cognitive training in treatment refractory schizophrenia. Neuropsychopharmacology.

[270] Iosifescu D. (2020). Are electroencephalogram-derived predictors of antidepressant efficacy closer to clinical usefulness?. JAMA Netw. Open.

[271] Kratzke I., Campbell A.M., Yefimov M., Mosaly P., Adapa K., Meltzer-Brody S., Farrell T., Mazur L. (2020). Pilot study using neurofeedback as a tool to reduce surgical resident burnout. J. Am. Coll. Surg..

[272] Blume M., Schmidt R., Schmidt J., Martin A., Hilbert A. (2021). EEG neurofeedback in the treatment of adults with binge-eating disorder: A randomized controlled pilot study. Neurotherapeutics.

[273] Murias M., Major S., Compton S., Buttinger J., Sun J.M., Kurtzberg J., Dawson G. (2018). Electrophysiological biomarkers predict clinical improvement in an open-label trial assessing efficacy of autologous umbilical cord blood for treatment of autism. Stem Cells Transl. Med..

[274] Parmar D., Enticott P., Albein-Urios N. (2021). Anodal HD-tDCS for cognitive inflexibility in autism spectrum disorder: A pilot study. Brain Stimul..

[275] Wang S.-Y., Lin I., Fan S., Tsai Y.-C., Yen C., Yeh Y., Huang M.-F., Lee Y., Chiu N., Hung C. (2019). The effects of alpha asymmetry and high-beta down-training neurofeedback for patients with the major depressive disorder and anxiety symptoms. J. Affect. Disord..

[276] Santopetro N., Kallen A.M., Threadgill A., Hajcak G. (2020). Reduced flanker P300 prospectively predicts increases in depression in female adolescents. Biol. Psychol..

[277] Tan P., Rozenman M., Chang S.W., Jurgiel J., Truong H., Piacentini J., Loo S. (2021). The ERN as a neural index of changes in performance monitoring following attention training in pediatric obsessive-compulsive disorder. Biol. Psychol..

[278] Chen T.-C., Lin I. (2020). The learning effects and curves during high beta down-training neurofeedback for patients with major depressive disorder. J. Affect. Disord..

[279] Yuan E.J., Chang C.H., Chen H.H., Huang S.-S. (2024). The effects of electroencephalography functional connectivity during emotional recognition among patients with major depressive disorder and healthy controls. J. Psychiatr. Res..

[280] Al-kaysi A.M., Al-Ani A., Loo C., Powell T.Y., Martin D., Breakspear M., Boonstra T. (2017). Predicting tDCS treatment outcomes of patients with major depressive disorder using automated EEG classification. J. Affect. Disord..

[281] John A., Schöllhorn W. (2018). Acute effects of instructed and self-created variable rope skipping on EEG brain activity and heart rate variability. Front. Behav. Neurosci..

[282] Ammar A., Boujelbane M., Simak M., Fraile-Fuente I., Rizzi N., Washif J., Żmijewski P., Jahrami H.A., Schöllhorn W. (2023). Unveiling the acute neurophysiological responses to strength training: An exploratory study on novices performing weightlifting bouts with different motor learning models. Biol. Sport.

[283] Anil K., Demain S., Burridge J., Simpson D., Taylor J., Cotter I., Vučković A. (2022). The importance of self-efficacy and negative affect for neurofeedback success for central neuropathic pain after a spinal cord injury. Sci. Rep..

[284] Baskaran A., Farzan F., Milev R., Brenner C.A., Alturi S., McAndrews M.P., Blier P., Evans K., Foster J., Frey B. (2018). The comparative effectiveness of electroencephalographic indices in predicting response to escitalopram therapy in depression: A pilot study. J. Affect. Disord..

[285] Azarpaikan A., Torbati H.T., Sohrabi M., Boostani R., Ghoshuni M. (2019). Power spectral parameter variations after transcranial direct current stimulation in a bimanual coordination task. Adapt. Behav..

[286] Bailey N., Hoy K., Rogasch N., Thomson R., McQueen S., Elliot D., Sullivan C., Fulcher B.D., Daskalakis Z., Fitzgerald P. (2018). Responders to rTMS for depression show increased fronto-midline theta and theta connectivity compared to non-responders. Brain Stimul..

[287] Barth B., Rohe T., Deppermann S., Fallgatter A., Ehlis A. (2021). Neural oscillatory responses to performance monitoring differ between high- and low-impulsive individuals, but are unaffected by TMS. Hum. Brain Mapp..

[288] Donaldson P., Kirkovski M., Rinehart N., Enticott P. (2019). A double-blind HD-tDCS/EEG study examining right temporoparietal junction involvement in facial emotion processing. Soc. Neurosci..

[289] Duma G.M., Mento G., Manari T., Martinelli M., Tressoldi P.E. (2017). Driving with intuition: A preregistered study about the EEG anticipation of simulated random car accidents. PLoS ONE.

[290] Gilbreath D., Hagood D., Alatorre-Cruz G.C., Andres A., Downs H., Larson-Prior L. (2023). Effects of early nutrition factors on baseline neurodevelopment during the first 6 months of life: An EEG study. Nutrients.

[291] Evans D., Sutton S., Oliver J.A., Drobes D. (2015). Cortical activity differs during nicotine deprivation versus satiation in heavy smokers. Psychopharmacology.

[292] Grosselin F., Breton A., Yahia-Cherif L., Wang X., Spinelli G., Hugueville L., Fossati P., Attal Y., Navarro-Sune X., Chavez M. (2021). Alpha activity neuromodulation induced by individual alpha-based neurofeedback learning in ecological context: A double-blind randomized study. Sci. Rep..

[293] Gangemi A., De Luca R., Fabio R., Lauria P., Rifici C., Pollicino P., Marra A., Olivo A., Quartarone A., Calabrò R.S. (2023). Effects of virtual reality cognitive training on neuroplasticity: A quasi-randomized clinical trial in patients with stroke. Biomedicines.

[294] Leodori G., Fabbrini A., Bartolo M.I., Costanzo M., Asci F., Palma V., Belvisi D., Conte A., Berardelli A. (2021). Cortical mechanisms underlying variability in intermittent theta-burst stimulation-induced plasticity: A TMS-EEG study. Clin. Neurophysiol..

[295] Li G.-S., Li H., Pu J., Wan F., Hu Y. (2020). Effect of brain alpha oscillation on the performance in laparoscopic skills simulator training. Surg. Endosc..

[296] Hasan M., Vučković A., Qazi S.A., Yousuf Z., Shahab S., Fraser M. (2021). Immediate effect of neurofeedback training on the pain matrix and cortical areas involved in processing neuropsychological functions. Neurol. Sci..

[297] Hill A., Rogasch N., Fitzgerald P., Hoy K. (2017). Effects of prefrontal bipolar and high-definition transcranial direct current stimulation on cortical reactivity and working memory in healthy adults. NeuroImage.

[298] Hill A., Rogasch N., Fitzgerald P., Hoy K. (2018). Effects of single versus dual-site High-Definition transcranial direct current stimulation (HD-tDCS) on cortical reactivity and working memory performance in healthy subjects. Brain Stimul..

[299] Wang J., Wu D., Shen Y., Zhang Y., Xu Y., Tang X., Wang R. (2015). Cognitive behavioral therapy eases orthodontic pain: EEG states and functional connectivity analysis. Oral Dis..

[300] Juras L., Hromatko I., Vranić A. (2025). Parietal alpha and theta power predict cognitive training gains in middle-aged adults. Front. Aging Neurosci..

[301] Yu K., Prasad I., Mir H., Thakor N., Al-Nashash H. (2015). Cognitive workload modulation through degraded visual stimuli: A single-trial EEG study. J. Neural Eng..

[302] Jones K.T., Johnson E., Berryhill M. (2020). Frontoparietal theta-gamma interactions track working memory enhancement with training and tDCS. NeuroImage.

[303] Kober S., Witte M., Grinschgl S., Neuper C., Wood G. (2018). Placebo hampers ability to self-regulate brain activity: A double-blind sham-controlled neurofeedback study. NeuroImage.

[304] Küssner M., de Groot A.M.B., Hofman W., Hillen M. (2016). EEG beta power but not background music predicts the recall scores in a foreign-vocabulary learning task. PLoS ONE.

[305] Lo L.-C., Hatfield B.D., Janjigian K., Wang Y.-S., Fong D., Hung T.-M. (2024). The effect of left temporal EEG neurofeedback training on cerebral cortical activity and precision cognitive-motor performance. Res. Q. Exerc. Sport.

[306] Ciria L.F., Luque-Casado A., Sanabria D., Holgado D., Ivanov P., Perakakis P. (2019). Oscillatory brain activity during acute exercise: Tonic and transient neural response to an oddball task. Psychophysiology.

[307] Bachman M.D., Watts A.T.M., Collins P., Bernat E. (2021). Sequential gains and losses during gambling feedback: Differential effects in time-frequency delta and theta measures. Psychophysiology.

[308] Lin M.-H., Baker T. (2022). A novel application of an adaptive filter to dissociate the effects of TMS on neural excitability and trial-to-trial latency jitter in event-related potentials. Brain Stimul..

[309] Best M.W., Gale D., Tran T.B., Haque M.K., Bowie C. (2017). Brief executive function training for individuals with severe mental illness: Effects on EEG synchronization and executive functioning. Schizophr. Res..

[310] Nagy B., Protzner A., van der Wijk G., Wang H., Cortese F., Czigler I., Gaál Z. (2022). The modulatory effect of adaptive task-switching training on resting-state neural network dynamics in younger and older adults. Sci. Rep..

[311] Nelson A., Ricci S., Tatti E., Panday P., Girau E., Lin J., Thomson B.O., Chen H., Marshall W., Tononi G. (2020). Neural fatigue due to intensive learning is reversed by a nap but not by quiet waking. Sleep.

[312] Nikolin S., Martin D., Loo C., Boonstra T. (2022). Transcranial direct current stimulation modulates working memory maintenance processes in healthy individuals. J. Cogn. Neurosci..

[313] De Pascalis V., Vecchio A., Cirillo G. (2020). Resting anxiety increases EEG delta–beta correlation: Relationships with the Reinforcement Sensitivity Theory personality traits. Pers. Individ. Differ..

[314] Paul M., Bellebaum C., Ghio M., Suchan B., Wolf O. (2020). Stress effects on learning and feedback-related neural activity depend on feedback delay. Psychophysiology.

[315] Nawaz R., Nisar H., Voon Y.V. (2020). Changes in spectral power and functional connectivity of response-conflict task after neurofeedback training. IEEE Access.

[316] Hack R.L., Aigner M., Musalek M., Crevenna R., Konicar L. (2024). Brain regulation training improves emotional competences in patients with alcohol use disorder. Soc. Cogn. Affect. Neurosci..

[317] Reteig L., van den Brink R.L., Prinssen S., Cohen M.X., Slagter H. (2019). Sustaining attention for a prolonged period of time increases temporal variability in cortical responses. Cortex.

[318] Robertson C., Marino F. (2015). Prefrontal and motor cortex EEG responses and their relationship to ventilatory thresholds during exhaustive incremental exercise. Eur. J. Appl. Physiol..

[319] Robertson C., Skein M., Wingfield G., Hunter J.R., Miller T., Hartmann T. (2023). Acute electroencephalography responses during incremental exercise in those with mental illness. Front. Psychiatry.

[320] Luijcks R., Vossen C.J., Hermens H., van Os J., Lousberg R. (2015). The influence of perceived stress on cortical reactivity: A proof-of-principle study. PLoS ONE.

[321] Wriessnegger S.C., Leitner M., Kostoglou K. (2024). The brain under pressure: Exploring neurophysiological responses to cognitive stress. Brain Cogn..

[322] Kim S., Yang C., Dong S.-Y., Lee S.-H. (2022). Predictions of tDCS treatment response in PTSD patients using EEG based classification. Front. Psychiatry.

[323] Jaiswal S., Tsai S.-Y., Juan C., Muggleton N., Liang W.-K. (2019). Low delta and high alpha power are associated with better conflict control and working memory in high mindfulness, low anxiety individuals. Soc. Cogn. Affect. Neurosci..

[324] Bhakta S.G., Cavanagh J., Talledo J., Kotz J.E., Benster L., Roberts B.Z., Nungaray J.A., Brigman J., Light G., Swerdlow N. (2022). EEG reveals that dextroamphetamine improves cognitive control through multiple processes in healthy participants. Neuropsychopharmacology.

[325] Sehatpour P., Dondé C., Hoptman M., Kreither J., Adair D., Dias E., Vail B., Rohrig S., Silipo G., Lopez-Calderon J. (2020). Network-level mechanisms underlying effects of transcranial direct current stimulation (tDCS) on visuomotor learning. NeuroImage.

[326] Liu S., Shi C., Meng H., Meng Y., Gong X., Chen X.-P., Tao L. (2023). Cognitive control subprocess deficits and compensatory modulation mechanisms in patients with frontal lobe injury revealed by EEG markers: A basic study to guide brain stimulation. Gen. Psychiatry.

[327] Strüber L., Baumont M., Barraud P., Nougier V., Cignetti F. (2021). Brain oscillatory correlates of visuomotor adaptive learning. NeuroImage.

[328] Chung S.W., Rogasch N., Hoy K., Sullivan C., Cash R., Fitzgerald P. (2018). Impact of different intensities of intermittent theta burst stimulation on the cortical properties during TMS-EEG and working memory performance. Hum. Brain Mapp..

[329] Xu T., Huang J., Pei Z., Chen J., Li J., Bezerianos A., Thakor N.V., Wang H. (2022). The effect of multiple factors on working memory capacities: Aging, task difficulty, and training. IEEE Trans. Biomed. Eng..

[330] Tatti E., Golemme M., Chrisostomou L.D., Panozzo G., Grande G., Bernardi C., Cappelletti M. (2017). P255 Electrophysiological and behavioral monitoring of learning: An EEG and tRNS combined study. Clin. Neurophysiol..

[331] Aktürk T., de Graaf T., Güntekin B., Hanoglu L., Sack A. (2022). Enhancing memory capacity by experimentally slowing theta frequency oscillations using combined EEG-tACS. Sci. Rep..

[332] Ulam F., Shelton C., Richards L., Davis L., Hunter B., Fregni F., Higgins K. (2015). Cumulative effects of transcranial direct current stimulation on EEG oscillations and attention/working memory during subacute neurorehabilitation of traumatic brain injury. Clin. Neurophysiol..

[333] da Paz V.K.C., Garcia A., da Paz Neto A.C., Tomaz C. (2018). SMR neurofeedback training facilitates working memory performance in healthy older adults: A behavioral and EEG study. Front. Behav. Neurosci..

[334] Hsu W.-Y., Zanto T.P., van Schouwenburg M.V., Gazzaley A. (2017). Enhancement of multitasking performance and neural oscillations by transcranial alternating current stimulation. PLoS ONE.

[335] Wischnewski M., Zerr P., Schutter D. (2016). Effects of theta transcranial alternating current stimulation over the frontal cortex on reversal learning. Brain Stimul..

[336] Kim W.-J., Lee Y.-S., Hong K.H., Choi H., Song J.-J., Hwang H.-J. (2025). Effect of transcutaneous auricular vagus nerve stimulation on stress regulation: An EEG and questionnaire study. Front. Digit. Health.

[337] Li Y., Tian C., Xu L., Pei L., Huang X., Wang X. (2025). EEG-Guided adaptive learning: A new neuroeducational approach to the facilitation of cognitive control in ADHD children. Child Care Health Dev..

[338] Sun Y., Giacobbe P., Tang C.W., Barr M., Rajji T., Kennedy S., Fitzgerald P., Lozano A., Wong W., Daskalakis Z. (2015). Deep brain stimulation modulates gamma oscillations and theta–gamma coupling in treatment resistant depression. Brain Stimul..

[339] Ke Y., Liu S., Chen L., Wang X., Ming D. (2023). Lasting enhancements in neural efficiency by multi-session transcranial direct current stimulation during working memory training. npj Sci. Learn..

[340] Zhang H., Chavarriaga R., Millán J. (2015). Discriminant brain connectivity patterns of performance monitoring at average and single-trial levels. NeuroImage.

[341] Tsang R.S., Stow D., Kwong A.S., Donnelly N.A., Fraser H., Barroso I., Khandaker G.M. (2025). Immunometabolic blood biomarkers of developmental trajectories of depressive symptoms: Findings from the ALSPAC birth cohort. Mol. Psychiatry.

[342] Abi-Dargham A., Moeller S.J., Ali F., DeLorenzo C., Domschke K., Horga G., Krystal J.H. (2023). Candidate biomarkers in psychiatric disorders: State of the field. World Psychiatry.

[343] Fatori D., Shephard E., Benette D., Naspolini N.F., Guzman G.C., Wang J.Y.T., Polanczyk G.V. (2025). Identifying biomarkers and trajectories of executive functions and language development in the first 3 years of life: Design, methods, and findings of the Germina cohort study. Dev. Psychopathol..

[344] Krainc D., Martin W.J., Casey B., Jensen F.E., Tishkoff S., Potter W.Z., Hyman S.E. (2023). Shifting the trajectory of therapeutic development for neurological and psychiatric disorders. Sci. Transl. Med..

[345] Wang Y., Ma L., Wang J., Ding Y., Liu N., Men W., Tao S. (2024). The neural and genetic underpinnings of different developmental trajectories of Attention-Deficit/Hyperactivity Symptoms in children and adolescents. BMC Med..

[346] Slevin H., Kehinde F., Begum-Ali J., Ellis C., Burkitt-Wright E., Green J., Garg S. (2024). Developmental trajectories in infants and pre-school children with Neurofibromatosis 1. Mol. Autism.

[347] Assaf R., Ouellet J., Bourque J., Stip E., Leyton M., Conrod P., Potvin S. (2022). A functional neuroimaging study of self-other processing alterations in atypical developmental trajectories of psychotic-like experiences. Sci. Rep..

[348] DeLouize A.M., Eick G., Karam S.D., Snodgrass J.J. (2022). Current and future applications of biomarkers in samples collected through minimally invasive methods for cancer medicine and population-based research. Am. J. Hum. Biol..

[349] Mielke M.M., Fowler N.R. (2024). Alzheimer disease blood biomarkers: Considerations for population-level use. Nat. Rev. Neurol..

[350] Gkintoni E., Halkiopoulos C. (2025). Digital twin cognition: AI-biomarker integration in biomimetic neuropsychology. Biomimetics.

[351] Siopis G., Porter J. (2022). Contribution of biological age-predictive biomarkers to nutrition research: A systematic review of the current evidence and implications for future research. Adv. Nutr..

[352] Alam M.K., Zaman M.U., Alqhtani N.R., Alqahtani A.S., Alqahtani F., Cicciù M., Minervini G. (2024). Salivary biomarkers and Temporomandibular disorders: A systematic review conducted according to PRISMA guidelines and the Cochrane Handbook for Systematic Reviews of Interventions. J. Oral Rehabil..

[353] Elguoshy A., Zedan H., Saito S. (2025). Machine learning-driven insights in cancer metabolomics: From subtyping to biomarker discovery and prognostic modeling. Metabolites.

[354] Gell M., Noble S., Laumann T.O., Nelson S.M., Tervo-Clemmens B. (2025). Psychiatric neuroimaging designs for individualised, cohort, and population studies. Neuropsychopharmacology.

[355] DeGroat W., Abdelhalim H., Patel K., Mendhe D., Zeeshan S., Ahmed Z. (2024). Discovering biomarkers associated and predicting cardiovascular disease with high accuracy using a novel nexus of machine learning techniques for precision medicine. Sci. Rep..

[356] Paganin W., Signorini S. (2024). Inflammatory biomarkers in depression: Scoping review. BJPsych Open.

[357] Xu X., Li J., Zhu Z., Zhao L., Wang H., Song C., Pei Y. (2024). A comprehensive review on synergy of multi-modal data and AI technologies in medical diagnosis. Bioengineering.

[358] Pratihar R., Sankar R. (2024). Advancements in Parkinson’s Disease Diagnosis: A comprehensive survey on biomarker integration and machine learning. Computers.

[359] Chen J., Yu K., Bi Y., Ji X., Zhang D. (2024). Strategic integration: A cross-disciplinary review of the fNIRS-EEG dual-modality imaging system for delivering multimodal neuroimaging to applications. Brain Sci..

[360] Steyaert S., Pizurica M., Nagaraj D., Khandelwal P., Hernandez-Boussard T., Gentles A.J., Gevaert O. (2023). Multimodal data fusion for cancer biomarker discovery with deep learning. Nat. Mach. Intell..

[361] Gkintoni E., Vantarakis A., Gourzis P. (2025). Insights into the public health burden of neuropsychiatric disorders: A systematic review of electroencephalography-based cognitive biomarkers. Medicina.

[362] Roshdy A., Karar A., Kork S.A., Beyrouthy T., Nait-ali A. (2024). Advancements in EEG emotion recognition: Leveraging multi-modal database integration. Appl. Sci..

[363] Boehm K.M., Khosravi P., Vanguri R., Gao J., Shah S.P. (2022). Harnessing multimodal data integration to advance precision oncology. Nat. Rev. Cancer.

[364] Alrawis M., Al-Ahmadi S., Mohammad F. (2024). Bridging modalities: A multimodal machine learning approach for Parkinson’s Disease diagnosis using EEG and MRI data. Appl. Sci..

[365] Rockholt M.M., Kenefati G., Doan L.V., Chen Z.S., Wang J. (2023). In search of a composite biomarker for chronic pain by way of EEG and machine learning: Where do we currently stand?. Front. Neurosci..

[366] Genovese A., Borna S., Gomez-Cabello C.A., Haider S.A., Prabha S., Forte A.J., Veenstra B.R. (2024). Artificial intelligence in clinical settings: A systematic review of its role in language translation and interpretation. Ann. Transl. Med..

[367] Geaney A., O’Reilly P., Maxwell P., James J.A., McArt D., Salto-Tellez M. (2023). Translation of tissue-based artificial intelligence into clinical practice: From discovery to adoption. Oncogene.

[368] Bernstam E.V., Shireman P.K., Meric-Bernstam F., Zozus M.N., Jiang X., Brimhall B.B., Becich M.J. (2022). Artificial intelligence in clinical and translational science: Successes, challenges and opportunities. Clin. Transl. Sci..

[369] Mohamed Y.A., Khanan A., Bashir M., Mohamed A.H.H., Adiel M.A., Elsadig M.A. (2024). The impact of artificial intelligence on language translation: A review. IEEE Access.

[370] Fahim Y.A., Hasani I.W., Kabba S., Ragab W.M. (2025). Artificial intelligence in healthcare and medicine: Clinical applications, therapeutic advances, and future perspectives. Eur. J. Med. Res..

[371] Alowais S.A., Alghamdi S.S., Alsuhebany N., Alqahtani T., Alshaya A.I., Almohareb S.N., Albekairy A.M. (2023). Revolutionizing healthcare: The role of artificial intelligence in clinical practice. BMC Med. Educ..

[372] Baxi V., Edwards R., Montalto M., Saha S. (2022). Digital pathology and artificial intelligence in translational medicine and clinical practice. Mod. Pathol..

[373] Lion K.C., Lin Y.H., Kim T. (2024). Artificial intelligence for language translation: The equity is in the details. JAMA.

[374] Keikhosrokiani P., Annunen J., Komulainen-Ebrahim J., Kortelainen J., Kallio M., Vieira P., Uusimaa J. (2025). Requirement analysis for data-driven electroencephalography seizure monitoring software to enhance quality and decision making in digital care pathways for epilepsy: A feasibility study from the perspectives of health care professionals. JMIR Hum. Factors.

[375] Biondi A., Simblett S.K., Viana P.F., Laiou P., Fiori A.M., Nurse E., Richardson M.P. (2024). Feasibility and acceptability of an ultra-long-term at-home EEG monitoring system (EEG@HOME) for people with epilepsy. Epilepsy Behav..

[376] Craik A., González-España J.J., Alamir A., Edquilang D., Wong S., Sánchez Rodríguez L., Contreras-Vidal J.L. (2023). Design and validation of a low-cost mobile EEG-based brain-computer interface. Sensors.

[377] Biondi A., Santoro V., Viana P.F., Laiou P., Pal D.K., Bruno E., Richardson M.P. (2022). Noninvasive mobile EEG as a tool for seizure monitoring and management: A systematic review. Epilepsia.

[378] Armand Larsen S., Klok L., Lehn-Schiøler W., Gatej R., Beniczky S. (2024). Low-cost portable EEG device for bridging the diagnostic gap in resource-limited areas. Epileptic Disord..

[379] Biondi A., Dursun E., Viana P.F., Laiou P., Richardson M.P. (2024). New wearable and portable EEG modalities in epilepsy: The views of hospital-based healthcare professionals. Epilepsy Behav..

[380] Alahaideb L., Al-Nafjan A., Aljumah H., Aldayel M. (2025). Brain-computer interface for EEG-based authentication: Advancements and practical implications. Sensors.

[381] Fidas C.A., Lyras D. (2023). A review of EEG-based user authentication: Trends and future research directions. IEEE Access.

[382] Ortega A.D.D., Camacho-Bustamante L.M., Navarro-Tuch S.A., Bustamante-Bello R. Proposal for an EEG-integrated tool for supporting cognitive assessment practices. Proceedings of the 2025 IEEE Mexican Humanitarian Technology Conference (MHTC).

