# Supplementary material for: Mapping the Digital Mind: A Meta-Analysis of EEG Biomarkers in Cognition, Emotion, and Mental Health
Source: Brain Sci. 2026 Mar 29;16(4):368. doi: 10.3390/brainsci16040368 (PMC13115222; doi:10.3390/brainsci16040368)
Supplement: Supplementary file 1 [file brainsci-16-00368-s001.zip › Table_S1_Manuscript_Final_MDM.pdf]

Supplementary Table S1

Characteristics of Studies Included in the Meta-Analysis

(k = 210 studies; N = 9,935 participants; 38 countries; 2015-2025)

Title: Mapping the Digital Mind: A Meta-Analysis of EEG Biomarkers in Cognition, Emotion, and Mental Health

RQ1: Cognitive Control & Executive Function [k = 35 studies]

Authors	Year	Design	Population	EEG Biomarker	Task/Paradigm
Adelhöfer et al.	2020	Experimental	Healthy adults	Theta	Cognitive task
Adelhöfer et al.	2019	Experimental	Healthy adults	Theta	Brain stimulation
Vahid et al.	2020	Experimental	Healthy adults	EEG/ERP	Cognitive task
Neuhäußer et al.	2023	Intervention	ADHD	Neural measures	Neurofeedback
Prochnow et al.	2024	Experimental	Healthy adults	Theta	Cognitive task
Winneke et al.	2019	Experimental	Healthy adults	Neural measures	Attention task
Barbazzeni et al.	2023	Intervention	Healthy adults	EEG/ERP	Neurofeedback
Barth et al.	2021	Randomized	Healthy adults	Neural measures	Neurofeedback
Sari et al.	2016	Intervention	Anxiety	Neural measures	Working memory
Lowe et al.	2018	Experimental	Healthy adults	Theta	Brain stimulation
Erb et al.	2019	Experimental	Healthy adults	EEG/ERP	Cognitive task
Liu et al.	2023	Experimental	Healthy adults	Alpha, ERN	Cognitive task
Dennis-Tiwary et al.	2016	Experimental	Anxiety	Neural measures	Attention task
Dierolf et al.	2017	Experimental	Healthy adults	EEG/ERP	Cognitive task
Incagli et al.	2019	Experimental	Healthy adults	EEG/ERP	Cognitive task
Bing-Canar et al.	2016	Experimental	Healthy adults	Alpha	Cognitive task
Wei et al.	2022	Intervention	Anxiety	EEG/ERP	Working memory
Nesterovsky et al.	2015	Experimental	ADHD	ERN	Attention task
Zhang et al.	2024	Randomized	Healthy adults	EEG/ERP	Emotion regulatio
Knoth et al.	2018	Experimental	Healthy adults	EEG/ERP	Cognitive task
Mückschel et al.	2020	Randomized	Healthy adults	Neural measures	Cognitive task
Nigbur et al.	2015	Experimental	Healthy adults	EEG/ERP	Attention task
Olfers et al.	2017	Intervention	Healthy adults	EEG/ERP	Attention task
Pietto et al.	2018	Intervention	Healthy adults	ERN	Cognitive task
Raghuraman et al.	2019	Experimental	Healthy adults	Neural measures	Cognitive task
Rauch et al.	2019	Experimental	Healthy adults	Neural measures	Cognitive task
Reis et al.	2016	Intervention	Older adults	Theta, Alpha	Neurofeedback
Olson et al.	2016	Experimental	Healthy adults	Neural measures	Cognitive task
Santarnecchi et al.	2017	Intervention	Healthy adults	EEG/ERP	Cognitive task
Schmeichel et al.	2016	Experimental	Healthy adults	Neural measures	Cognitive task
Chung et al.	2018	Experimental	Healthy adults	Theta	Brain stimulation
Ligeza et al.	2018	Randomized	Healthy adults	N2	Cognitive task
Zhao et al.	2020	Intervention	Anxiety	EEG/ERP	Working memory
Li et al.	2017	Experimental	Healthy adults	Neural measures	Cognitive task
van der Kolk et al.	2016	RCT	PTSD	Neural measures	Neurofeedback

RQ2: Learning, Memory & Cognitive Training [k = 34 studies]

Authors	Year	Design	Population	EEG Biomarker	Task/Paradigm
Parsons et al.	2021	Intervention	Healthy adults	EEG/ERP	Neurofeedback
Wirth et al.	2019	Experimental	Healthy adults	EEG/ERP	Cognitive task
Duan et al.	2022	Intervention	Healthy adults	SMR	Cognitive task
Alberca-Reina et al.	2015	Experimental	Healthy adults	Neural measures	Memory task
Fearnbach et al.	2017	Experimental	Children/Adol.	Neural measures	Cognitive task
Gram et al.	2015	Experimental	Healthy adults	Neural measures	Cognitive task
Guez et al.	2015	Randomized	Healthy adults	Neural measures	Neurofeedback
Volpert-Esmond et al	2018	Experimental	Healthy adults	Neural measures	Cognitive task
Zhang et al.	2023	Experimental	Healthy adults	Neural measures	Cognitive task
Hsueh et al.	2016	Intervention	Healthy adults	Alpha	Neurofeedback
Wang et al.	2020	Experimental	Healthy adults	Neural measures	Cognitive task
Jochumsen et al.	2019	Experimental	Healthy adults	EEG/ERP	Brain stimulation
Eschmann et al.	2022	Intervention	Healthy adults	Theta	Neurofeedback
Eschmann et al.	2020	Intervention	Healthy adults	Theta	Neurofeedback

Kis et al.	2017	Experimental	Healthy adults	EEG/ERP	Cognitive task
Kober et al.	2015	Intervention	Stroke	EEG/ERP	Neurofeedback
Kober et al.	2019	Intervention	Healthy adults	Neural measures	Neurofeedback
Lau et al.	2017	Experimental	Healthy adults	Neural measures	Cognitive task
Chen et al.	2023	Experimental	Healthy adults	EEG/ERP	Brain stimulation
Manuel et al.	2018	Experimental	Healthy adults	Neural measures	Resting-state
Mariman et al.	2023	Experimental	Healthy adults	Neural measures	Motor task
Kodama et al.	2023	Intervention	Healthy adults	EEG/ERP	Neurofeedback
Murphy et al.	2018	Intervention	Healthy adults	Neural measures	Cognitive task
Pinter et al.	2021	Pilot	Healthy adults	EEG/ERP	Neurofeedback
Pugin et al.	2015	Intervention	Children/Adol.	Neural measures	Working memory
Rozengurt et al.	2016	Intervention	Healthy adults	Theta	Neurofeedback
Sampedro-Piquero et	2024	Experimental	Healthy adults	ERN	Resting-state
Schranz et al.	2022	Experimental	Stroke	Neural measures	Brain stimulation
Chung et al.	2017	Experimental	Healthy adults	Theta	Brain stimulation
Chung et al.	2019	Experimental	Healthy adults	Neural measures	Brain stimulation
Wang et al.	2022	Intervention	Healthy adults	Neural measures	Neurofeedback
Nan et al.	2020	Intervention	Healthy adults	Alpha	Neurofeedback
Pourbehbahani et al.	2023	Intervention	Healthy adults	Neural measures	Neurofeedback
Liu et al.	2016	RCT	ADHD	Alpha	Working memory

RQ3: Emotion Regulation & Affective Processing [k = 61 studies]

**Authors**	**Year**	**Design**	**Population**	**EEG Biomarker**	**Task/Paradigm**
Naas et al.	2019	Intervention	Healthy adults	Alpha	Neurofeedback
Albein-Urios et al.	2022	Experimental	Healthy adults	ERN	Emotion regulatio
Sibalis et al.	2019	Intervention	ADHD	EEG/ERP	Attention task
Arazi et al.	2017	Experimental	Healthy adults	Neural measures	Cognitive task
Bigliassi et al.	2020	Experimental	Healthy adults	Neural measures	Cognitive task
Brown et al.	2022	Intervention	Healthy adults	Neural measures	Emotion regulatio
Ciorciari et al.	2019	Experimental	Healthy adults	EEG/ERP	Cognitive task
Compton et al.	2017	Experimental	Healthy adults	Neural measures	Cognitive task
Cao et al.	2017	Experimental	Healthy adults	Theta	Brain stimulation
Dennis-Tiwary et al.	2017	Experimental	Anxiety	Neural measures	Attention task
Mohan et al.	2016	Experimental	Healthy adults	Neural measures	Cognitive task
Pan et al.	2019	Experimental	Anxiety	Neural measures	Attention task
Mizrahi et al.	2025	Experimental	Healthy adults	Neural measures	Cognitive task
Duan et al.	2015	Experimental	Healthy adults	EEG/ERP	Cognitive task
Engelbregt et al.	2016	Intervention	Healthy adults	EEG/ERP	Neurofeedback
Garland et al.	2025	Experimental	Healthy adults	ERN	Emotion regulatio
Faehling et al.	2015	Experimental	Healthy adults	LPP	Brain stimulation
Tian et al.	2021	Experimental	Healthy adults	Neural measures	Cognitive task
Fischer et al.	2017	Experimental	Healthy adults	ERN	Cognitive task
Friedrich et al.	2015	Intervention	ASD	Neural measures	Neurofeedback
Gladhill et al.	2022	Experimental	Healthy adults	Neural measures	Cognitive task
Goldway et al.	2019	Experimental	Healthy adults	Neural measures	Cognitive task
Kim et al.	2020	Experimental	Healthy adults	EEG/ERP	Cognitive task
Hill et al.	2022	Experimental	Healthy adults	LPP	Cognitive task
Hsieh et al.	2024	Experimental	Athletes	Neural measures	Cognitive task
Fietz et al.	2025	Intervention	Children/Adol.	EEG/ERP	Neurofeedback
Ortmann et al.	2025	Experimental	Healthy adults	Neural measures	Cognitive task
Kolijn et al.	2019	RCT	Children/Adol.	EEG/ERP	Cognitive task
Koller-Schlaud et al	2021	Experimental	Depression	Neural measures	Cognitive task
Lackner et al.	2016	Intervention	Children/Adol.	EEG/ERP	Neurofeedback
Dickey et al.	2023	Experimental	Depression	Neural measures	Emotion regulatio
Wu et al.	2024	Experimental	Anxiety	Neural measures	Emotion regulatio
Loheswaran et al.	2017	Experimental	Healthy adults	Neural measures	Cognitive task
Lohse et al.	2020	Experimental	Healthy adults	Neural measures	Cognitive task
Magee et al.	2023	Experimental	Depression	Neural measures	Go/NoGo
Mallorquí-Bagué et a	2020	Experimental	Healthy adults	ERN	Emotion regulatio
Marlats et al.	2020	Pilot	Older adults	Theta, SMR	Neurofeedback
Mavros et al.	2022	Experimental	Healthy adults	EEG/ERP	Cognitive task
Mayer et al.	2021	Experimental	Healthy adults	Neural measures	Attention task
McFarland et al.	2015	Intervention	Healthy adults	Neural measures	Motor task
Mennella et al.	2017	Intervention	Anxiety	Alpha asym.	Neurofeedback
Hu et al.	2019	Experimental	Healthy adults	Neural measures	Cognitive task
Egana-delSol et al.	2023	Experimental	Healthy adults	Neural measures	Emotion regulatio

Parr et al.	2019	Intervention	Healthy adults	Alpha	Attention task
Perchtold-Stefan et	2023	Experimental	Anxiety	Neural measures	Cognitive task
Poole et al.	2021	Experimental	Anxiety	Theta, Beta	Cognitive task
Rodriguez-Larios et	2024	Intervention	Healthy adults	Neural measures	Cognitive task
Eldeeb et al.	2021	Experimental	ASD	EEG/ERP	Cognitive task
Schreiter et al.	2018	Experimental	Healthy adults	Neural measures	Cognitive task
Zeng et al.	2021	Experimental	Healthy adults	EEG/ERP	Memory task
Li et al.	2024	Intervention	Depression	Neural measures	Attention task
Stolz et al.	2022	Experimental	Healthy adults	Theta	Cognitive task
Chandra et al.	2016	Experimental	Healthy adults	EEG/ERP	Cognitive task
Tipple et al.	2024	Pilot	Healthy adults	Neural measures	Neurofeedback
Ligeza et al.	2022	Experimental	Depression	LPP	Cognitive task
Muralidharan et al.	2019	Experimental	Healthy adults	Beta	Motor task
Lin et al.	2020	Experimental	Healthy adults	EEG/ERP	Cognitive task
Allen et al.	2021	Experimental	Healthy adults	EEG/ERP	Cognitive task
Wiens et al.	2022	Experimental	Healthy adults	Neural measures	Cognitive task
Li et al.	2025	Intervention	Healthy adults	Theta	Neurofeedback
Xu et al.	2018	Experimental	Healthy adults	Alpha asym.	Cognitive task

RQ4: Mental Health & Clinical Applications [k = 19 studies]

**Authors**	**Year**	**Design**	**Population**	**EEG Biomarker**	**Task/Paradigm**
Haendel et al.	2021	Experimental	ASD	Neural measures	Cognitive task
Arns et al.	2016	Randomized	Depression	Alpha asym.	Cognitive task
Arns et al.	2015	Experimental	Depression	Theta	Cognitive task
Schwartzmann et al.	2024	Experimental	Depression	Neural measures	Cognitive task
Bryant et al.	2021	Experimental	PTSD	EEG/ERP	Cognitive task
Rolle et al.	2020	Randomized	Depression	Neural measures	Cognitive task
Diaz Hernandez et al	2015	Intervention	Schizophrenia	ERN	Neurofeedback
Kang et al.	2019	Experimental	Anxiety	ERN	Cognitive task
Hochberger et al.	2018	Intervention	Schizophrenia	Neural measures	Cognitive task
Iosifescu et al.	2020	Experimental	Depression	Neural measures	Cognitive task
Kratzke et al.	2020	Pilot	Burnout/MDD (healthcare workers)	Neural measures	Neurofeedback
Blume et al.	2021	RCT	Healthy adults	EEG/ERP	Neurofeedback
Murias et al.	2018	Experimental	ADHD	Neural measures	Cognitive task
Parmar et al.	2021	Pilot	ADHD	Neural measures	Brain stimulation
Wang et al.	2019	Intervention	Depression	Alpha asym., Beta	Neurofeedback
Santopetro et al.	2020	Experimental	Depression	P300	Flanker
Tan et al.	2021	Intervention	Healthy adults	ERN	Attention task
Chen et al.	2020	Intervention	Depression	Beta	Neurofeedback
Yuan et al.	2024	Experimental	Depression	Neural measures	Cognitive task

RQ5: Neural Oscillations & Biomarker Methodology [k = 61 studies]

**Authors**	**Year**	**Design**	**Population**	**EEG Biomarker**	**Task/Paradigm**
Al-kaysi et al.	2017	Experimental	Depression	EEG/ERP	Brain stimulation
John et al.	2018	Experimental	Healthy adults	EEG/ERP	Cognitive task
Ammar et al.	2023	Intervention	Athletes	Neural measures	Motor task
Anil et al.	2022	Intervention	Healthy adults	Neural measures	Neurofeedback
Baskaran et al.	2018	Pilot	Depression	Neural measures	Cognitive task
Azarpaikan et al.	2019	Experimental	Healthy adults	Neural measures	Brain stimulation
Bailey et al.	2018	Experimental	Depression	Theta	Cognitive task
Barth et al.	2021	Experimental	Healthy adults	Neural measures	Cognitive task
Donaldson et al.	2019	Experimental	Healthy adults	EEG/ERP	Brain stimulation
Duma et al.	2017	Experimental	Healthy adults	EEG/ERP	Cognitive task
Gilbreath et al.	2023	Experimental	Healthy adults	EEG/ERP	Cognitive task
Evans et al.	2015	Experimental	Healthy adults	Neural measures	Cognitive task
Grosselin et al.	2021	Randomized	Healthy adults	Alpha	Neurofeedback
Gangemi et al.	2023	Randomized	Stroke	Neural measures	Cognitive task
Leodori et al.	2021	Experimental	Healthy adults	Theta	Brain stimulation
Li et al.	2020	Intervention	Healthy adults	Alpha	Cognitive task
Hasan et al.	2021	Intervention	Healthy adults	Neural measures	Neurofeedback
Hill et al.	2017	Experimental	Healthy adults	Neural measures	Working memory
Hill et al.	2018	Experimental	Healthy adults	Neural measures	Working memory
Wang et al.	2015	Experimental	Healthy adults	EEG/ERP	Cognitive task
Juras et al.	2025	Intervention	Older adults	Theta, Alpha	Cognitive task
Yu et al.	2015	Experimental	Healthy adults	EEG/ERP	Cognitive task

Jones et al.	2020	Intervention	Healthy adults	Theta, Gamma	Working memory
Kober et al.	2018	Intervention	Healthy adults	Neural measures	Neurofeedback
Küssner et al.	2016	Experimental	Healthy adults	Beta	Cognitive task
Lo et al.	2024	Intervention	Athletes	EEG/ERP	Neurofeedback
Ciria et al.	2019	Experimental	Healthy adults	Neural measures	Cognitive task
Bachman et al.	2021	Experimental	Healthy adults	Theta, ERN	Cognitive task
Lin et al.	2022	Experimental	Healthy adults	Neural measures	Cognitive task
Best et al.	2017	Intervention	Schizophrenia	EEG/ERP	Cognitive task
Nagy et al.	2022	Intervention	Older adults	Neural measures	Resting-state
Nelson et al.	2020	Experimental	Healthy adults	Neural measures	Cognitive task
Nikolin et al.	2022	Experimental	Healthy adults	Neural measures	Working memory
De Pascalis et al.	2020	Experimental	Anxiety	Beta	Resting-state
Paul et al.	2020	Experimental	Healthy adults	Neural measures	Cognitive task
Nawaz et al.	2020	Intervention	Healthy adults	Neural measures	Neurofeedback
Hack et al.	2024	Intervention	Healthy adults	Neural measures	Emotion regulatio
Reteig et al.	2019	Experimental	Healthy adults	Neural measures	Attention task
Robertson et al.	2015	Experimental	Healthy adults	EEG/ERP	Motor task
Robertson et al.	2023	Experimental	Healthy adults	Neural measures	Cognitive task
Luijcks et al.	2015	Experimental	Healthy adults	Neural measures	Cognitive task
Wriessnegger et al.	2024	Experimental	Healthy adults	Neural measures	Cognitive task
Kim et al.	2022	Experimental	PTSD	EEG/ERP	Brain stimulation
Jaiswal et al.	2019	Experimental	Anxiety	Alpha	Working memory
Bhakta et al.	2022	Experimental	Healthy adults	EEG/ERP	Cognitive task
Sehatpour et al.	2020	Experimental	Healthy adults	Neural measures	Brain stimulation
Liu et al.	2023	Experimental	Healthy adults	EEG/ERP	Brain stimulation
Strüber et al.	2021	Experimental	Healthy adults	Neural measures	Motor task
Chung et al.	2018	Experimental	Healthy adults	Theta	Working memory
Xu et al.	2022	Intervention	Older adults	Neural measures	Working memory
Tatti et al.	2017	Experimental	Healthy adults	ERN	Cognitive task
Aktürk et al.	2022	Experimental	Healthy adults	Theta	Memory task
Ulam et al.	2015	Experimental	PTSD	EEG/ERP	Working memory
da Paz et al.	2018	Intervention	Older adults	SMR	Neurofeedback
Hsu et al.	2017	Experimental	Healthy adults	ERN	Brain stimulation
Wischnewski et al.	2016	Experimental	Healthy adults	Theta, ERN	Brain stimulation
Kim et al.	2025	Experimental	Healthy adults	EEG/ERP	Brain stimulation
Li et al.	2025	Experimental	ADHD	EEG/ERP	Cognitive task
Sun et al.	2015	Experimental	Depression	Theta, Gamma	Brain stimulation
Ke et al.	2023	Intervention	Healthy adults	Neural measures	Working memory
Zhang et al.	2015	Experimental	Healthy adults	ERN	Cognitive task

Note: Studies are organized by research question domain. Study characteristics were inferred from reference information. Abbreviations: FMθ = frontal-midline theta; LPP = late positive potential; Alpha asym. = frontal alpha asymmetry; ERD = event-related desynchronization; SMR = sensorimotor rhythm; ERN = error-related negativity.
