# Supplementary material for: Mapping the Digital Mind: A Meta-Analysis of EEG Biomarkers in Cognition, Emotion, and Mental Health
Source: Brain Sci. 2026 Mar 29;16(4):368. doi: 10.3390/brainsci16040368 (PMC13115222; doi:10.3390/brainsci16040368)
Supplement: Supplementary file 1 [file brainsci-16-00368-s001.zip › Table_S2_PRISMA_2020_checklist_MDM.pdf]

PRISMA 2020 Checklist

Section and Topic	Item #	Checklist item	Location where item is reported
**TITLE Mapping the Digital Mind: A Meta-Analysis of EEG Biomarkers in Cognition, Emotion, and Mental Health**			
Title	1	Identify the report as a systematic review.	Title page: "Mapping the Digital Mind: A Meta-Analysis of EEG Biomarkers in Cognition, Emotion, and Mental Health"
ABSTRACT			
Abstract	2	See the PRISMA 2020 for Abstracts checklist.	Abstract section: Structured abstract with Background, Methods, Results, and Conclusions subsections
INTRODUCTION			
Rationale	3	Describe the rationale for the review in the context of existing knowledge.	Section 1.1-1.7: Background and rationale covering EEG methodology, cognitive control, learning, emotion, and clinical applications
Objectives	4	Provide an explicit statement of the objective(s) or question(s) the review addresses.	Section 1.8: Five research questions (RQ1-RQ5) explicitly stated with specific aims
**METHODS**			
Eligibility criteria	5	Specify the inclusion and exclusion criteria for the review and how studies were grouped for the syntheses.	Section 2.3.1 (Inclusion) and 2.3.2 (Exclusion)
Information sources	6	Specify all databases, registers, websites, organisations, reference lists and other sources searched or consulted to identify studies. Specify the date when each source was last searched or consulted.	Section 2.3: PubMed/MEDLINE (n=1,423), PsycINFO (n=892), Web of Science (n=1,012), Scopus (n=520)
Search strategy	7	Present the full search strategies for all databases, registers and websites, including any filters and limits used.	Section 2.3: Search terms for EEG, cognition, emotion regulation, mental health; date range 2015-2025
Selection process	8	Specify the methods used to decide whether a study met the inclusion criteria of the review, including how many reviewers screened each record and each report retrieved, whether they worked independently, and if applicable, details of automation tools used in the process.	Section 2.4: Two independent reviewers; Figure 1 (PRISMA flow diagram); Cohen's $\kappa = 0.89$ inter-rater reliability
Data collection process	9	Specify the methods used to collect data from reports, including how many reviewers collected data from each report, whether they worked independently, any processes for obtaining or confirming data from study investigators, and if applicable, details of automation tools used in the process.	Section 2.4: Standardized data extraction form; dual independent coding; discrepancies resolved by consensus
Data items	10a	List and define all outcomes for which data were sought. Specify whether all results that were compatible with each outcome domain in each study were sought (e.g. for all measures, time points, analyses), and if not, the methods used to decide which results to collect.	Section 2.2.1: Primary outcomes (EEG biomarkers: FMθ, LPP, alpha, N2, ERN, P300); Secondary outcomes (behavioral measures)
	10b	List and define all other variables for which data were sought (e.g. participant and intervention characteristics, funding sources). Describe any assumptions made about any missing or unclear information.	Section 2.4: Study design, sample size, population characteristics, paradigm type, statistical data for effect size calculation
Study risk of bias assessment	11	Specify the methods used to assess risk of bias in the included studies, including details of the tool(s) used, how many reviewers assessed each study and whether they worked independently, and if applicable, details of automation tools used in the process.	Section 2.5: Cochrane RoB 2 for RCTs; Newcastle-Ottawa Scale for observational studies; two independent raters
Effect measures	12	Specify for each outcome the effect measure(s) (e.g. risk ratio, mean difference) used in	Section 2.6: Cohen's d (standardized mean difference) with 95%

Section and Topic	Item #	Checklist item	Location where item is reported
		the synthesis or presentation of results.	confidence intervals; Hedges' g correction for small samples
Synthesis methods	13a	Describe the processes used to decide which studies were eligible for each synthesis (e.g. tabulating the study intervention characteristics and comparing against the planned groups for each synthesis (item #5)).	Section 2.6: Studies grouped by research question (RQ1-RQ5); Section 3.1 describes distribution across domains
	13b	Describe any methods required to prepare the data for presentation or synthesis, such as handling of missing summary statistics, or data conversions.	Section 2.6: Effect sizes calculated from means/SDs, t-values, F-values, or reported statistics; formulas specified
	13c	Describe any methods used to tabulate or visually display results of individual studies and syntheses.	Section 2.6: Forest plots (Figures 2-7), funnel plots (Figure 8), summary tables (Tables 1-4)
	13d	Describe any methods used to synthesize results and provide a rationale for the choice(s). If meta-analysis was performed, describe the model(s), method(s) to identify the presence and extent of statistical heterogeneity, and software package(s) used.	Section 2.6: Random-effects models (REML estimator); Cochran's Q, I^2, τ^2; R packages metafor and meta
	13e	Describe any methods used to explore possible causes of heterogeneity among study results (e.g. subgroup analysis, meta-regression).	Section 2.6: Subgroup analyses by clinical condition; moderator analyses; meta-regression for continuous variables
	13f	Describe any sensitivity analyses conducted to assess robustness of the synthesized results.	Section 2.6: Leave-one-out analysis; Section 3.9: Sensitivity analyses; quality-based sensitivity analysis
Reporting bias assessment	14	Describe any methods used to assess risk of bias due to missing results in a synthesis (arising from reporting biases).	Section 2.6: Funnel plots, Egger's regression test (p-values reported), Duval & Tweedie trim-and-fill method
Certainty assessment	15	Describe any methods used to assess certainty (or confidence) in the body of evidence for an outcome.	Section 2.5: Quality assessment via risk of bias tools; Discussion Section 4: GRADE-informed evaluation of evidence certainty
RESULTS			
Study selection	16a	Describe the results of the search and selection process, from the number of records identified in the search to the number of studies included in the review, ideally using a flow diagram.	Section 3.1; Figure 1: 3,847 initial records → 2,955 after deduplication → 210 included (k = 210)
	16b	Cite studies that might appear to meet the inclusion criteria, but which were excluded, and explain why they were excluded.	Section 3.1: 114 articles excluded with reasons (57 no EEG biomarker, 32 no relevant outcome, 25 insufficient data)
Study characteristics	17	Cite each included study and present its characteristics.	Table 1 (summary by RQ); Table S1 (complete characteristics for k = 210 studies); References [131-340]
Risk of bias in studies	18	Present assessments of risk of bias for each included study.	Section 3.8: 71% high quality (n=149), 24% moderate (n=50), 5% low (n=11); 76% RCTs low risk of bias
Results of individual studies	19	For all outcomes, present, for each study: (a) summary statistics for each group (where appropriate) and (b) an effect estimate and its precision (e.g. confidence/credible interval), ideally using structured tables or plots.	Sections 3.2-3.7: Forest plots (Figures 2-7) with individual study effects, 95% CIs, and weights
Results of syntheses	20a	For each synthesis, briefly summarise the characteristics and risk of bias among contributing studies.	Table 1: Summary characteristics by RQ including study count, N, design types, quality distribution
	20b	Present results of all statistical syntheses conducted. If meta-analysis was done, present for each the summary estimate and its precision (e.g. confidence/credible interval) and measures of statistical heterogeneity. If comparing groups, describe the direction of the effect.	Sections 3.2-3.7: Pooled d with 95% CI; I^2 and τ^2 for each analysis; Table 4 comprehensive summary
	20c	Present results of all investigations of possible causes of heterogeneity among study results.	Sections 3.2.2, 3.4.1-3.4.3, 3.5.2: Moderator analyses; clinical condition subgroups explain I^2 = 75.4% in RQ4

PRISMA 2020 Checklist

Section and Topic	Item #	Checklist item	Location where item is reported
	20d	Present results of all sensitivity analyses conducted to assess the robustness of the synthesized results.	Section 3.9: Leave-one-out analysis shows stable estimates; quality sensitivity analysis confirms robustness
Reporting biases	21	Present assessments of risk of bias due to missing results (arising from reporting biases) for each synthesis assessed.	Section 3.8: Egger's test p-values (RQ1 $p=.032$, RQ3 $p<.001$, RQ5 $p<.001$); trim-and-fill $\Delta d < 0.02$
Certainty of evidence	22	Present assessments of certainty (or confidence) in the body of evidence for each outcome assessed.	Section 4 Discussion: $I^2 = 0.0\%$ for 6/7 analyses indicates high replicability; GRADE-informed certainty ratings
DISCUSSION			
Discussion	23a	Provide a general interpretation of the results in the context of other evidence.	Section 4.1-4.4: Interpretation of biomarker findings in context of cognitive neuroscience and clinical literature
	23b	Discuss any limitations of the evidence included in the review.	Section 4.6: Technical and methodological considerations; heterogeneity in RQ4; sample size limitations
	23c	Discuss any limitations of the review processes used.	Section 4.7: Limitations including language bias (English only), publication bias, heterogeneous paradigms
	23d	Discuss implications of the results for practice, policy, and future research.	Section 4.5: Clinical decision-making implications; Section 4.8: Four-phase implementation framework; Section 5: Future directions
OTHER INFORMATION			
Registration and protocol	24a	Provide registration information for the review, including register name and registration number, or state that the review was not registered.	Section 2.1: Open Science Framework registration (osf.io/aunks); registered prior to data extraction
	24b	Indicate where the review protocol can be accessed, or state that a protocol was not prepared.	Section 2.1: Protocol pre-registered with OSF and available at osf.io/aunks
	24c	Describe and explain any amendments to information provided at registration or in the protocol.	No amendments to registered protocol reported
Support	25	Describe sources of financial or non-financial support for the review, and the role of the funders or sponsors in the review.	Funding statement: "This research received no external funding"
Competing interests	26	Declare any competing interests of review authors.	Conflicts of Interest statement: "The authors declare no conflicts of interest"
Availability of data, code and other materials	27	Report which of the following are publicly available and where they can be found: template data collection forms; data extracted from included studies; data used for all analyses; analytic code; any other materials used in the review.	Data Availability Statement: Extraction data and analytic code available upon reasonable request from corresponding author

From: Page MJ, McKenzie JE, Bossuyt PM, Boutron I, Hoffmann TC, Mulrow CD, et al. The PRISMA 2020 statement: an updated guideline for reporting systematic reviews. BMJ 2021;372:n71. doi: 10.1136/bmj.n71.
This work is licensed under CC BY 4.0. To view a copy of this license, visit https://creativecommons.org/licenses/by/4.0/
