# Supplementary material for: Mapping the Digital Mind: A Meta-Analysis of EEG Biomarkers in Cognition, Emotion, and Mental Health
Source: Brain Sci. 2026 Mar 29;16(4):368. doi: 10.3390/brainsci16040368 (PMC13115222; doi:10.3390/brainsci16040368)
Supplement: Supplementary file 1 [file brainsci-16-00368-s001.zip › Table_S3_Quality Assessment_MDM.pdf]

TABLE S3: Quality Assessment for All 210 Studies

Quality assessed using Newcastle-Ottawa Scale (NOS) for observational/experimental studies (max 9 stars: Selection 0–4 stars, Comparability 0–2 stars, Outcome/Exposure 0–3 stars) and Cochrane Risk of Bias Tool 2.0 (RoB 2.0) for RCTs. Overall thresholds: High quality = NOS ≥ 7 stars or RoB Low risk; Moderate = NOS 5–6 or RoB Some Concerns; Low quality = NOS < 5 or RoB High Risk. Inter-rater reliability for all quality assessments: Cohen's κ = 0.89 (95% CI [0.85, 0.93]). For RCTs, NOS domain scores are not applicable (—); RoB 2.0 overall judgement is reported instead.

Summary: Quality Distribution (k = 210)

High quality (NOS ≥ 7 or RoB Low): n = 123 (58.6%)
Moderate quality (NOS 5–6 or RoB Some concerns): n = 87 (41.4%)
Low quality (NOS < 5 or RoB High risk): n = 0
Overall inter-rater reliability: Cohen's κ = 0.89

RQ1: Cognitive Control & Executive Function (k=35)

Study (Author, Year)	Design	Tool	Selection (0-4)	Compar. (0-2)	Outcome (0-3)	Total	Quality	Risk of Bias
Adelhöfer & Beste (2020)	Experimental	NOS	4	2	1	**7**	High quality	**Low**
Adelhöfer & Beste (2019)	Experimental	NOS	4	2	1	**7**	High quality	**Low**
Vahid et al. (2020)	Experimental	NOS	4	2	2	**8**	High quality	**Low**
Neuhäußer et al. (2023)	Intervention (RCT)	RoB 2.0	—	—	—	**—**	Some concerns	**Moderate**
Prochnow et al. (2024)	Experimental	NOS	4	2	1	**7**	High quality	**Low**
Winneke et al. (2019)	Experimental	NOS	4	2	1	**7**	High quality	**Low**
Barbazzeni et al. (2023)	Neurofeedback	NOS	3	2	1	**6**	Moderate quality	**Moderate**
Barth et al. (2021)	RCT	RoB 2.0	—	—	—	**—**	Low	**Low**
Sari et al. (2016)	Intervention	NOS	4	2	1	**7**	High quality	**Low**
Lowe et al. (2018)	Experimental	NOS	3	2	1	**6**	Moderate quality	**Moderate**
Erb et al. (2019)	Experimental	NOS	4	2	1	**7**	High quality	**Low**
Liu et al. (2023)	Experimental	NOS	4	2	1	**7**	High quality	**Low**
Dennis-Tiwary et al. (2016)	Experimental	NOS	4	2	1	**7**	High quality	**Low**
Dierolf et al. (2017)	Experimental	NOS	4	2	2	**8**	High quality	**Low**
Incagli et al. (2019)	Experimental	NOS	3	2	1	**6**	Moderate quality	**Moderate**
Bing-Canar et al. (2016)	Experimental	NOS	3	2	1	**6**	Moderate quality	**Moderate**
Wei et al. (2022)	Intervention	NOS	4	2	1	**7**	High quality	**Low**
Nesterovsky et al. (2015)	Experimental	NOS	3	2	1	**6**	Moderate quality	**Moderate**
Zhang et al. (2024)	RCT	RoB 2.0	—	—	—	**—**	Low	**Low**
Knoth et al. (2018)	Experimental	NOS	3	2	1	**6**	Moderate quality	**Moderate**
Mückschel et al. (2020)	RCT	RoB 2.0	—	—	—	**—**	Low	**Low**
Nigbur et al. (2015)	Experimental	NOS	4	2	1	**7**	High quality	**Low**
Olfers et al. (2017)	Intervention	NOS	3	2	1	**6**	Moderate quality	**Moderate**

Pietto et al. (2018)	Intervention	NOS	3	2	1	**6**	Moderate quality	**Moderate**
Raghuraman et al. (2019)	Experimental	NOS	3	2	1	**6**	Moderate quality	**Moderate**
Rauch et al. (2019)	Experimental	NOS	4	2	1	**7**	High quality	**Low**
Reis et al. (2016)	Neurofeedback	NOS	3	2	1	**6**	Moderate quality	**Moderate**
Olson et al. (2016)	Experimental	NOS	4	2	1	**7**	High quality	**Low**
Santarnecchi et al. (2017)	Intervention	NOS	3	2	1	**6**	Moderate quality	**Moderate**
Schmeichel et al. (2016)	Experimental	NOS	4	2	1	**7**	High quality	**Low**
Chung et al. (2018)	Experimental	NOS	4	2	1	**7**	High quality	**Low**
Ligeza et al. (2018)	RCT	RoB 2.0	—	—	—	**—**	Low	**Low**
Zhao et al. (2020)	Intervention	NOS	4	2	1	**7**	High quality	**Low**
Li et al. (2017)	Experimental	NOS	3	2	1	**6**	Moderate quality	**Moderate**
van der Kolk et al. (2016)	RCT	RoB 2.0	—	—	—	**—**	Low	**Low**

RQ2: Learning, Memory & Cognitive Training (k=34)

Study (Author, Year)	Design	Tool	Selection (0-4)	Compar. (0-2)	Outcome (0-3)	Total	Quality	Risk of Bias
Parsons et al. (2021)	Neurofeedback	NOS	4	2	1	**7**	High quality	**Low**
Wirth et al. (2019)	Experimental	NOS	4	2	1	**7**	High quality	**Low**
Duan et al. (2022)	Neurofeedback	NOS	3	2	1	**6**	Moderate quality	**Moderate**
Alberca-Reina et al. (2015)	Experimental	NOS	3	2	1	**6**	Moderate quality	**Moderate**
Fearnbach et al. (2017)	Experimental	NOS	3	2	1	**6**	Moderate quality	**Moderate**
Gram et al. (2015)	Experimental	NOS	3	2	1	**6**	Moderate quality	**Moderate**
Guez et al. (2015)	RCT	RoB 2.0	—	—	—	**—**	Low	**Low**
Volpert-Esmond et al. (2018)	Experimental	NOS	4	2	1	**7**	High quality	**Low**
Zhang et al. (2023)	Experimental	NOS	4	2	1	**7**	High quality	**Low**
Hsueh et al. (2016)	Neurofeedback	NOS	3	2	1	**6**	Moderate quality	**Moderate**
Wang et al. (2020)	Experimental	NOS	4	2	1	**7**	High quality	**Low**
Jochumsen et al. (2019)	Experimental	NOS	3	2	1	**6**	Moderate quality	**Moderate**
Eschmann et al. (2022)	Neurofeedback	NOS	4	2	1	**7**	High quality	**Low**
Eschmann et al. (2020)	Neurofeedback	NOS	4	2	1	**7**	High quality	**Low**
Kis et al. (2017)	Experimental	NOS	4	2	1	**7**	High quality	**Low**
Kober et al. (2015)	Neurofeedback	NOS	3	2	1	**6**	Moderate quality	**Moderate**
Kober et al. (2019)	Neurofeedback	NOS	4	2	1	**7**	High quality	**Low**
Lau et al. (2017)	Experimental	NOS	3	2	1	**6**	Moderate quality	**Moderate**
Chen et al. (2023)	Experimental	NOS	3	2	1	**6**	Moderate quality	**Moderate**

Manuel et al. (2018)	Experimental	NOS	4	2	1	**7**	High quality	**Low**
Mariman et al. (2023)	Experimental	NOS	4	2	1	**7**	High quality	**Low**
Kodama et al. (2023)	Neurofeedback	NOS	3	2	1	**6**	Moderate quality	**Moderate**
Murphy et al. (2018)	Intervention	NOS	3	2	1	**6**	Moderate quality	**Moderate**
Pinter et al. (2021)	Pilot	NOS	3	1	1	**5**	Moderate quality	**Moderate**
Pugin et al. (2015)	Intervention	NOS	3	2	1	**6**	Moderate quality	**Moderate**
Rozengurt et al. (2016)	Neurofeedback	NOS	3	2	1	**6**	Moderate quality	**Moderate**
Sampedro-Piquero et al. (2024)	Experimental	NOS	3	2	1	**6**	Moderate quality	**Moderate**
Schranz et al. (2022)	Experimental	NOS	3	2	1	**6**	Moderate quality	**Moderate**
Chung et al. (2017)	Experimental	NOS	4	2	1	**7**	High quality	**Low**
Chung et al. (2019)	Experimental	NOS	4	2	1	**7**	High quality	**Low**
Wang et al. (2022)	Intervention	NOS	4	2	1	**7**	High quality	**Low**
Nan et al. (2020)	Neurofeedback	NOS	3	2	1	**6**	Moderate quality	**Moderate**
Pourbehbahani et al. (2023)	Neurofeedback	NOS	3	1	1	**5**	Moderate quality	**Moderate**
Liu et al. (2016)	RCT	RoB 2.0	—	—	—	**—**	Low	**Low**

RQ3: Emotion Regulation & Affective Processing (k=61)

Study (Author, Year)	Design	Tool	Selection (0-4)	Compar. (0-2)	Outcome (0-3)	Total	Quality	Risk of Bias
Naas et al. (2019)	Neurofeedback	NOS	4	2	1	**7**	High quality	**Low**
Albein-Urios et al. (2022)	Experimental	NOS	4	2	1	**7**	High quality	**Low**
Sibalis et al. (2019)	Neurofeedback	NOS	3	2	1	**6**	Moderate quality	**Moderate**
Arazi et al. (2017)	Experimental	NOS	3	2	1	**6**	Moderate quality	**Moderate**
Bigliassi et al. (2020)	Experimental	NOS	4	2	1	**7**	High quality	**Low**
Brown et al. (2022)	Intervention	NOS	4	2	1	**7**	High quality	**Low**
Ciorciari et al. (2019)	Experimental	NOS	3	2	1	**6**	Moderate quality	**Moderate**
Compton et al. (2017)	Experimental	NOS	4	2	1	**7**	High quality	**Low**
Cao et al. (2017)	Experimental	NOS	4	2	1	**7**	High quality	**Low**
Dennis-Tiwary et al. (2017)	Experimental	NOS	4	2	1	**7**	High quality	**Low**
Mohan et al. (2016)	Experimental	NOS	3	2	1	**6**	Moderate quality	**Moderate**
Pan et al. (2019)	Experimental	NOS	4	2	1	**7**	High quality	**Low**
Mizrahi et al. (2025)	Experimental	NOS	4	2	1	**7**	High quality	**Low**
Duan et al. (2015)	Experimental	NOS	3	2	1	**6**	Moderate quality	**Moderate**
Engelbregt et al. (2016)	Neurofeedback	NOS	3	2	1	**6**	Moderate quality	**Moderate**
Garland et al. (2025)	Experimental	NOS	4	2	1	**7**	High quality	**Low**
Faehling et al. (2015)	Experimental	NOS	3	2	1	**6**	Moderate quality	**Moderate**

Tian et al. (2021)	Experimental	NOS	4	2	1	**7**	High quality	**Low**
Fischer et al. (2017)	Experimental	NOS	4	2	1	**7**	High quality	**Low**
Friedrich et al. (2015)	Neurofeedback	NOS	3	2	1	**6**	Moderate quality	**Moderate**
Gladhill et al. (2022)	Experimental	NOS	4	2	1	**7**	High quality	**Low**
Goldway et al. (2019)	Experimental	NOS	4	2	1	**7**	High quality	**Low**
Kim et al. (2020)	Experimental	NOS	4	2	1	**7**	High quality	**Low**
Hill et al. (2022)	Experimental	NOS	4	2	1	**7**	High quality	**Low**
Hsieh et al. (2024)	Experimental	NOS	3	2	1	**6**	Moderate quality	**Moderate**
Fietz et al. (2025)	Neurofeedback	NOS	3	2	1	**6**	Moderate quality	**Moderate**
Ortmann et al. (2025)	Experimental	NOS	4	2	1	**7**	High quality	**Low**
Kolijn et al. (2019)	RCT	RoB 2.0	—	—	—	**—**	Low	**Low**
Koller-Schlaud et al. (2021)	Experimental	NOS	4	2	1	**7**	High quality	**Low**
Lackner et al. (2016)	Neurofeedback	NOS	3	2	1	**6**	Moderate quality	**Moderate**
Dickey et al. (2023)	Experimental	NOS	4	2	1	**7**	High quality	**Low**
Wu et al. (2024)	Experimental	NOS	4	2	1	**7**	High quality	**Low**
Loheswaran et al. (2017)	Experimental	NOS	3	2	1	**6**	Moderate quality	**Moderate**
Lohse et al. (2020)	Experimental	NOS	4	2	1	**7**	High quality	**Low**
Magee et al. (2023)	Experimental	NOS	4	2	1	**7**	High quality	**Low**
Mallorquí-Bagué et al. (2020)	Experimental	NOS	3	2	1	**6**	Moderate quality	**Moderate**
Marlats et al. (2020)	Pilot	NOS	3	1	1	**5**	Moderate quality	**Moderate**
Mavros et al. (2022)	Experimental	NOS	3	2	1	**6**	Moderate quality	**Moderate**
Mayer et al. (2021)	Experimental	NOS	4	2	1	**7**	High quality	**Low**
McFarland et al. (2015)	Neurofeedback	NOS	3	2	1	**6**	Moderate quality	**Moderate**
Mennella et al. (2017)	Neurofeedback	NOS	4	2	1	**7**	High quality	**Low**
Hu et al. (2019)	Experimental	NOS	4	2	1	**7**	High quality	**Low**
Egana-delSol et al. (2023)	Experimental	NOS	3	2	1	**6**	Moderate quality	**Moderate**
Parr et al. (2019)	Neurofeedback	NOS	3	2	1	**6**	Moderate quality	**Moderate**
Perchtold-Stefan et al. (2023)	Experimental	NOS	4	2	1	**7**	High quality	**Low**
Poole et al. (2021)	Experimental	NOS	4	2	1	**7**	High quality	**Low**
Rodriguez-Larios et al. (2024)	Neurofeedback	NOS	3	2	1	**6**	Moderate quality	**Moderate**
Eldeeb et al. (2021)	Experimental	NOS	3	2	1	**6**	Moderate quality	**Moderate**
Schreiter et al. (2018)	Experimental	NOS	4	2	1	**7**	High quality	**Low**
Zeng et al. (2021)	Experimental	NOS	4	2	1	**7**	High quality	**Low**
Li et al. (2024)	Neurofeedback	NOS	4	2	1	**7**	High quality	**Low**
Stolz et al. (2022)	Experimental	NOS	4	2	1	**7**	High quality	**Low**

Chandra et al. (2016)	Experimental	NOS	3	2	1	**6**	Moderate quality	**Moderate**
Tipple et al. (2024)	Pilot	NOS	3	1	1	**5**	Moderate quality	**Moderate**
Ligeza et al. (2022)	Experimental	NOS	4	2	1	**7**	High quality	**Low**
Muralidharan et al. (2019)	Experimental	NOS	3	2	1	**6**	Moderate quality	**Moderate**
Lin et al. (2020)	Experimental	NOS	4	2	1	**7**	High quality	**Low**
Allen et al. (2021)	Experimental	NOS	4	2	1	**7**	High quality	**Low**
Wiens et al. (2022)	Experimental	NOS	4	2	1	**7**	High quality	**Low**
Li et al. (2025)	Neurofeedback	NOS	3	2	1	**6**	Moderate quality	**Moderate**
Xu et al. (2018)	Experimental	NOS	4	2	1	**7**	High quality	**Low**

RQ4: Mental Health & Clinical Applications (k=19)

Study (Author, Year)	Design	Tool	Selection (0-4)	Compar. (0-2)	Outcome (0-3)	Total	Quality	Risk of Bias
Haendel et al. (2021)	Experimental	NOS	4	2	1	**7**	High quality	**Low**
Arns et al. (2016)	RCT (multicentre)	RoB 2.0	—	—	—	**—**	Low	**Low**
Arns et al. (2015)	Experimental	NOS	4	2	2	**8**	High quality	**Low**
Schwartzmann et al. (2024)	Experimental	NOS	4	2	1	**7**	High quality	**Low**
Bryant et al. (2021)	Experimental	NOS	4	2	1	**7**	High quality	**Low**
Rolle et al. (2020)	RCT	RoB 2.0	—	—	—	**—**	Low	**Low**
Diaz Hernandez et al. (2015)	Neurofeedback	NOS	3	2	1	**6**	Moderate quality	**Moderate**
Kang et al. (2019)	Experimental	NOS	4	2	1	**7**	High quality	**Low**
Hochberger et al. (2018)	Intervention	NOS	3	2	1	**6**	Moderate quality	**Moderate**
Iosifescu et al. (2020)	Experimental	NOS	4	2	1	**7**	High quality	**Low**
Kratzke et al. (2020)	Pilot	NOS	3	1	1	**5**	Moderate quality	**Moderate**
Blume et al. (2021)	RCT	RoB 2.0	—	—	—	**—**	Low	**Low**
Murias et al. (2018)	Experimental	NOS	4	2	1	**7**	High quality	**Low**
Parmar et al. (2021)	Pilot	RoB 2.0	—	—	—	**—**	Some concerns	**Moderate**
Wang et al. (2019)	Neurofeedback	NOS	3	2	1	**6**	Moderate quality	**Moderate**
Santopetro et al. (2020)	Experimental	NOS	4	2	1	**7**	High quality	**Low**
Tan et al. (2021)	Neurofeedback	NOS	3	2	1	**6**	Moderate quality	**Moderate**
Chen et al. (2020)	Neurofeedback	NOS	3	2	1	**6**	Moderate quality	**Moderate**
Yuan et al. (2024)	Experimental	NOS	4	2	1	**7**	High quality	**Low**

RQ5: Neural Oscillations & Biomarker Methodology (k=61)

Study (Author, Year)	Design	Tool	Selection (0-4)	Compar. (0-2)	Outcome (0-3)	Total	Quality	Risk of Bias

Al-kaysi et al. (2017)	Experimental	NOS	4	2	1	**7**	High quality	**Low**
John et al. (2018)	Experimental	NOS	3	2	1	**6**	Moderate quality	**Moderate**
Ammar et al. (2023)	Neurofeedback	NOS	3	2	1	**6**	Moderate quality	**Moderate**
Anil et al. (2022)	Neurofeedback	NOS	3	2	1	**6**	Moderate quality	**Moderate**
Baskaran et al. (2018)	Pilot	NOS	3	1	1	**5**	Moderate quality	**Moderate**
Azarpaikan et al. (2019)	Experimental	NOS	3	2	1	**6**	Moderate quality	**Moderate**
Bailey et al. (2018)	Experimental	NOS	4	2	1	**7**	High quality	**Low**
Barth et al. (2021)	Experimental	NOS	4	2	1	**7**	High quality	**Low**
Donaldson et al. (2019)	Experimental	NOS	4	2	1	**7**	High quality	**Low**
Duma et al. (2017)	Experimental	NOS	4	2	1	**7**	High quality	**Low**
Gilbreath et al. (2023)	Experimental	NOS	4	2	1	**7**	High quality	**Low**
Evans et al. (2015)	Experimental	NOS	4	2	1	**7**	High quality	**Low**
Grosselin et al. (2021)	RCT	RoB 2.0	—	—	—	**—**	Low	**Low**
Gangemi et al. (2023)	RCT	RoB 2.0	—	—	—	**—**	Low	**Low**
Leodori et al. (2021)	Experimental	NOS	4	2	1	**7**	High quality	**Low**
Li et al. (2020)	Neurofeedback	NOS	3	2	1	**6**	Moderate quality	**Moderate**
Hasan et al. (2021)	Neurofeedback	NOS	3	2	1	**6**	Moderate quality	**Moderate**
Hill et al. (2017)	Experimental	NOS	4	2	1	**7**	High quality	**Low**
Hill et al. (2018)	Experimental	NOS	4	2	1	**7**	High quality	**Low**
Wang et al. (2015)	Experimental	NOS	3	2	1	**6**	Moderate quality	**Moderate**
Juras et al. (2025)	Neurofeedback	NOS	3	2	1	**6**	Moderate quality	**Moderate**
Yu et al. (2015)	Experimental	NOS	3	2	1	**6**	Moderate quality	**Moderate**
Jones et al. (2020)	Neurofeedback	NOS	4	2	1	**7**	High quality	**Low**
Kober et al. (2018)	Neurofeedback	NOS	4	2	1	**7**	High quality	**Low**
Küssner et al. (2016)	Experimental	NOS	3	2	1	**6**	Moderate quality	**Moderate**
Lo et al. (2024)	Neurofeedback	NOS	3	2	1	**6**	Moderate quality	**Moderate**
Ciria et al. (2019)	Experimental	NOS	4	2	1	**7**	High quality	**Low**
Bachman et al. (2021)	Experimental	NOS	4	2	1	**7**	High quality	**Low**
Lin et al. (2022)	Experimental	NOS	4	2	1	**7**	High quality	**Low**
Best et al. (2017)	Intervention	NOS	3	2	1	**6**	Moderate quality	**Moderate**
Nagy et al. (2022)	Neurofeedback	NOS	3	2	1	**6**	Moderate quality	**Moderate**
Nelson et al. (2020)	Experimental	NOS	4	2	1	**7**	High quality	**Low**
Nikolin et al. (2022)	Experimental	NOS	4	2	1	**7**	High quality	**Low**
De Pascalis et al. (2020)	Experimental	NOS	4	2	1	**7**	High quality	**Low**

Paul et al. (2020)	Experimental	NOS	4	2	1	**7**	High quality	**Low**
Nawaz et al. (2020)	Neurofeedback	NOS	3	2	1	**6**	Moderate quality	**Moderate**
Hack et al. (2024)	Neurofeedback	NOS	3	2	1	**6**	Moderate quality	**Moderate**
Reteig et al. (2019)	Experimental	NOS	4	2	1	**7**	High quality	**Low**
Robertson et al. (2015)	Experimental	NOS	4	2	1	**7**	High quality	**Low**
Robertson et al. (2023)	Experimental	NOS	4	2	1	**7**	High quality	**Low**
Luijcks et al. (2015)	Experimental	NOS	3	2	1	**6**	Moderate quality	**Moderate**
Wriessnegger et al. (2024)	Experimental	NOS	3	2	1	**6**	Moderate quality	**Moderate**
Kim et al. (2022)	Experimental	NOS	4	2	1	**7**	High quality	**Low**
Jaiswal et al. (2019)	Experimental	NOS	3	2	1	**6**	Moderate quality	**Moderate**
Bhakta et al. (2022)	Experimental	NOS	4	2	1	**7**	High quality	**Low**
Sehatpour et al. (2020)	Experimental	NOS	4	2	1	**7**	High quality	**Low**
Liu et al. (2023)	Experimental	NOS	4	2	1	**7**	High quality	**Low**
Strüber et al. (2021)	Experimental	NOS	4	2	1	**7**	High quality	**Low**
Chung et al. (2018)	Experimental	NOS	4	2	1	**7**	High quality	**Low**
Xu et al. (2022)	Neurofeedback	NOS	3	2	1	**6**	Moderate quality	**Moderate**
Tatti et al. (2017)	Experimental	NOS	4	2	1	**7**	High quality	**Low**
Aktürk et al. (2022)	Experimental	NOS	3	2	1	**6**	Moderate quality	**Moderate**
Ulam et al. (2015)	Experimental	NOS	4	2	1	**7**	High quality	**Low**
da Paz et al. (2018)	Neurofeedback	NOS	3	1	1	**5**	Moderate quality	**Moderate**
Hsu et al. (2017)	Experimental	NOS	4	2	1	**7**	High quality	**Low**
Wischnewski et al. (2016)	Experimental	NOS	4	2	1	**7**	High quality	**Low**
Kim et al. (2025)	Experimental	NOS	4	2	1	**7**	High quality	**Low**
Li et al. (2025)	Experimental	NOS	4	2	1	**7**	High quality	**Low**
Sun et al. (2015)	Experimental	NOS	3	2	1	**6**	Moderate quality	**Moderate**
Ke et al. (2023)	Neurofeedback	NOS	3	2	1	**6**	Moderate quality	**Moderate**
Zhang et al. (2015)	Experimental	NOS	3	2	1	**6**	Moderate quality	**Moderate**
