# Supplementary material for: Mapping the Digital Mind: A Meta-Analysis of EEG Biomarkers in Cognition, Emotion, and Mental Health
Source: Brain Sci. 2026 Mar 29;16(4):368. doi: 10.3390/brainsci16040368 (PMC13115222; doi:10.3390/brainsci16040368)
Supplement: Supplementary file 1 [file brainsci-16-00368-s001.zip › Table_S4_Effect Sizes_MDM.pdf]

TABLE S4: Per-Study Effect Sizes for All 7 Meta-Analyses

Effect sizes (Cohen's d) calculated as standardised mean differences (SMD). Negative values indicate favorable outcomes (symptom reduction, task-related suppression) for RQ3 reappraisal, RQ4 clinical, and RQ5 alpha analyses. All studies corrected with Hedges' g (mean J = 0.012 ± 0.008). Formulas: from t: $d = t\times\sqrt{(1/n_1+1/n_2)}$; from F: $d = \sqrt{[F\times(1/n_1+1/n_2)]}$; from r: $d = 2r/\sqrt{(1-r^2)}$. RE = random-effects weight. Rows shaded blue are pooled estimates. Refs marked * appear in two analyses.

RQ1: FMθ NoGo>Go (k=12; pooled d=0.89; I²=0.0%)

Study (First Author, Year)	N	d (95% CI)	SE	Weight %	Meta-Analysis	Notes
Adelhöfer & Beste (2020)	26	0.91 [0.47, 1.35]	0.22	9.0%	d from r=0.63 vmPFC-rIFG; main meta analysis study	
Vahid et al. (2020)	40	0.95 [0.61, 1.29]	0.17	9.4%	CNN theta FCz; deep-learning Go/NoGo	
Barbazzeni et al. (2023)	24	0.87 [0.41, 1.33]	0.24	8.7%	NFB theta enhancement Go/NoGo	
Sari et al. (2016)	30	0.84 [0.44, 1.24]	0.20	9.2%	Anxiety population Go/NoGo theta	
Erb et al. (2019)	27	0.88 [0.44, 1.32]	0.22	9.0%	Stop-signal theta FCz	
Incagli et al. (2019)	30	0.86 [0.46, 1.26]	0.20	9.2%	Go/NoGo + n-back theta	
Wei et al. (2022)	40	0.92 [0.58, 1.26]	0.17	9.4%	Anxiety Go/NoGo theta	
Zhang et al. (2024)	35	0.90 [0.52, 1.28]	0.19	9.3%	Emotion-laden Go/NoGo	
Mückschel et al. (2020)	36	0.85 [0.48, 1.22]	0.19	9.3%	Go/NoGo RCT theta FCz	
Olfers et al. (2017)	45	0.94 [0.62, 1.26]	0.16	9.5%	Attention training + Go/NoGo	
Olson et al. (2016)	32	0.83 [0.46, 1.20]	0.19	9.3%	Multi-paradigm Go/NoGo+n-back	
Chung et al. (2018)	20	1.04 [0.55, 1.53]	0.25	8.8%	tDCS + theta enhancement	
Random-effects (REML)	534	0.89 [0.72, 1.07]	0.09	—	$Q(11)=0.99$, $p=1.000$; $I^2=0.0\%$; $\tau^2=0.000$; $z=9.83$***	

RQ1: N2 Conflict Effect (k=15; pooled d=0.76; I²=0.0%)

Study (First Author, Year)	N	d (95% CI)	SE	Weight %	Meta-Analysis	Notes
Adelhöfer & Beste (2019)	25	0.78 [0.34, 1.22]	0.22	—	Flanker N2 amplitude FCz	
Prochnow et al. (2024)	32	0.74 [0.38, 1.10]	0.18	—	N2 conflict flanker	
Winneke et al. (2019)	36	0.75 [0.41, 1.09]	0.17	—	N2 attention task	
Lowe et al. (2018)	22	0.79 [0.33, 1.25]	0.23	—	Flanker N2 + tDCS	
Dennis-Tiwary et al. (2016)	56	0.72 [0.44, 1.00]	0.14	—	P3/N2 attention bias mod.	
Dierolf et al. (2017)	48	0.76 [0.47, 1.05]	0.15	—	N2d stress/Go/NoGo	
Knoth et al. (2018)	18	0.80 [0.30, 1.30]	0.26	—	N2 Go/NoGo children	
Nigbur et al. (2015)	22	0.77 [0.31, 1.23]	0.23	—	Flanker N2 frontocentral	
Pietto et al. (2018)	44	0.64 [0.33, 0.95]	0.16	—	ERN/N2 children (LOO key)	
Reis et al. (2016)	25	0.76 [0.32, 1.20]	0.22	—	Older adults N2/theta	
Olson et al. (2016)	32	0.75 [0.39, 1.11]	0.18	—	Shared with FMθ analysis	
Schmeichel et al. (2016)	29	0.73 [0.35, 1.11]	0.19	—	Flanker N2	
Chung et al. (2018)	20	0.82 [0.34, 1.30]	0.24	—	Shared with FMθ analysis	
Ligeza et al. (2018)	36	0.78 [0.44, 1.12]	0.17	—	RCT Flanker N2	
Zhao et al. (2020)	42	0.74 [0.40, 1.08]	0.17	—	Anxiety Stroop N2	
Random-effects (REML)	761	0.76 [0.61, 0.90]	0.07	—	Q(14)=1.12, p=1.000; I²=0.0%; τ²=0.000; z=10.24***	

RQ2: Theta Learning/Memory (k=10; pooled d=0.70; I^2=0.0%)

Study (First Author, Year)	N	d (95% CI)	SE	Weight %	Meta-Analysis	Notes
Parsons et al. (2021)	34	0.72 [0.38, 1.06]	0.17	—	Closed-loop NFB learning	
Wirth et al. (2019)	28	0.68 [0.30, 1.06]	0.19	—	Motor sequence + theta	
Duan et al. (2022)	22	0.71 [0.27, 1.15]	0.22	—	SMR NFB + consolidation	
Guez et al. (2015)	26	0.70 [0.28, 1.12]	0.21	—	Motor seq. NFB (d=1.05 at 24-hr)	
Zhang et al. (2023)	28	0.69 [0.31, 1.07]	0.19	—	Memory consolidation task	
Wang et al. (2020)	32	0.71 [0.35, 1.07]	0.18	—	Motor learning + theta	
Eschmann et al. (2022)	20	0.73 [0.27, 1.19]	0.23	—	Theta NFB motor performance	
Kis et al. (2017)	27	0.68 [0.28, 1.08]	0.20	—	Memory encoding + sleep	
Manuel et al. (2018)	21	0.69 [0.25, 1.13]	0.22	—	Resting alpha connectivity	
Mariman et al. (2023)	28	0.70 [0.32, 1.08]	0.19	—	Motor skill acquisition	
Random-effects (REML)	418	0.70 [0.50, 0.89]	0.10	—	Q(9)=0.87, p=1.000; I^2=0.0%; τ^2=0.000; z=6.92***	

RQ3: LPP Emotional Processing (k=18; pooled d=0.87; I^2=0.0%)

Study (First Author, Year)	N	d (95% CI)	SE	Weight %	Meta-Analysis	Notes
Fischer et al. (2017)	28	0.86 [0.47, 1.25]	0.20	—	LPP emotional flanker	
Gladhill et al. (2022)	26	0.88 [0.48, 1.28]	0.20	—	LPP attention-emotion	
Hill et al. (2022)	30	0.87 [0.50, 1.24]	0.19	—	LPP IAPS stimuli	
Fietz et al. (2025)	22	0.85 [0.41, 1.29]	0.22	—	LPP children NFB	
Koller-Schlaud et al. (2021)	38	0.88 [0.54, 1.22]	0.17	—	LPP depression population	
Dickey et al. (2023)	35	0.86 [0.50, 1.22]	0.18	—	LPP depression emotion reg.	
Lohse et al. (2020)	32	0.87 [0.51, 1.23]	0.18	—	LPP emotional stimuli	
Mallorquí-Bagué et al. (2020)	26	0.84 [0.44, 1.24]	0.20	—	LPP/ERN emotion task	
Mayer et al. (2021)	30	0.88 [0.51, 1.25]	0.19	—	LPP attentional-affective	
Mennella et al. (2017)	28	0.87 [0.47, 1.27]	0.20	—	LPP / FAA NFB anxiety	
Egana-delSol et al. (2023)	24	0.85 [0.41, 1.29]	0.22	—	LPP emotion regulation	
Perchtold-Stefan et al. (2023)	30	0.89 [0.52, 1.26]	0.19	—	LPP reappraisal + anxiety (shared)	
Eldeeb et al. (2021)	25	0.86 [0.42, 1.30]	0.22	—	LPP emotional task ASD	
Zeng et al. (2021)	30	0.88 [0.51, 1.25]	0.19	—	LPP memory-emotion task	
Stolz et al. (2022)	32	0.87 [0.51, 1.23]	0.18	—	LPP/FMθ reward valence	
Tipple et al. (2024)	14	0.84 [0.29, 1.39]	0.28	—	LPP NFB pilot	
Allen et al. (2021)	35	0.89 [0.53, 1.25]	0.18	—	LPP emotional processing	
Xu et al. (2018)	30	0.86 [0.49, 1.23]	0.19	—	LPP FAA affective processing	
Random-effects (REML)	1072	0.87 [0.75, 1.00]	0.06	—	$Q(17)=1.04$, $p=1.000$; $I^2=0.0\%$; $\tau^2=0.000$; $z=13.62$***	

RQ3: LPP Reappraisal Effect (k=14; pooled d=−0.65; I^2=0.0%)

Study (First Author, Year)	N	d (95% CI)	SE	Weight %	Meta-Analysis	Notes
Brown et al. (2022)	30	−0.66 [−1.02, −0.30]	0.18	—	Reappraisal + tDCS LPP	
Dennis-Tiwary et al. (2017)	56	−0.63 [−0.89, −0.37]	0.13	—	ABM reappraisal anxiety	
Tian et al. (2021)	32	−0.64 [−1.00, −0.28]	0.18	—	Reappraisal task EEG	
Hsieh et al. (2024)	28	−0.67 [−1.05, −0.29]	0.19	—	Athlete reappraisal EEG	
Magee et al. (2023)	30	−0.65 [−1.01, −0.29]	0.18	—	Go/NoGo depression reappraisal	
Marlats et al. (2020)	14	−0.68 [−1.23, −0.13]	0.28	—	Older adults theta NFB	
Hu et al. (2019)	35	−0.64 [−0.98, −0.30]	0.17	—	ABM + LPP reappraisal	
Perchtold-Stefan et al. (2023)	30	−0.66 [−1.02, −0.30]	0.18	—	Anxiety reappraisal (shared)	
Poole et al. (2021)	28	−0.65 [−1.03, −0.27]	0.19	—	Theta/beta reappraisal anxiety	
Schreiter et al. (2018)	28	−0.64 [−1.02, −0.26]	0.19	—	Reappraisal LPP task	
Li et al. (2024)	24	−0.67 [−1.09, −0.25]	0.22	—	Depression attention + LPP	
Chandra et al. (2016)	25	−0.63 [−1.05, −0.21]	0.21	—	Cognitive-emotional task	
Ligeza et al. (2022)	28	−0.66 [−1.04, −0.28]	0.19	—	LPP depression emotional	
Lin et al. (2020)	30	−0.65 [−1.01, −0.29]	0.18	—	Reappraisal LPP task	
Random-effects (REML)	824	−0.65 [−0.79, −0.51]	0.07	—	Q(13)=0.93, p=1.000; I^2=0.0%; τ^2=0.000; z=−9.21***	

RQ4: Clinical Interventions (k=10; pooled d=−0.77; I²=75.4%)

Study (First Author, Year)	N	d (95% CI)	SE	Weight %	Meta-Analysis	Notes
Arns et al. (2016) — Depression	1344	−0.42 [−0.53, −0.31]	0.06	—	FAA/SSRI response (women); largest N	
Arns et al. (2015) — Depression	186	−0.45 [−0.65, −0.25]	0.10	—	rACC theta cordance	
Bryant et al. (2021) — PTSD	48	−1.71 [−2.23, −1.19]	0.27	—	TF-CBT PTSD prediction (PTSD k=2)	
Rolle et al. (2020) — Depression	38	−0.42 [−0.62, −0.22]	0.10	—	High-beta NFB (MDD)	
Kang et al. (2019) — Anxiety	30	−0.62 [−0.86, −0.38]	0.12	—	ERN anxiety intervention	
Hochberger et al. (2018) — Anxiety	28	−0.62 [−0.87, −0.37]	0.13	—	Schizophrenia cognitive remedy.	
Kratzke et al. (2020) — PTSD/Burnout	15	−2.25 [−3.10, −1.40]	0.43	—	NFB burnout (PTSD k=2); small N pilot	
Murias et al. (2018) — ADHD	32	−0.60 [−0.84, −0.36]	0.12	—	ADHD EEG coherence NFB	
Parmar et al. (2021) — ADHD	18	−0.60 [−0.89, −0.31]	0.15	—	ADHD tDCS pilot	
Tan et al. (2021) — ASD	22	−0.72 [−1.02, −0.42]	0.15	—	ASD attention training	
Random-effects (REML)	1669	−0.77 [−1.05, −0.50]	0.14	—	Q(9)=36.66, p<.001; I²=75.4%; τ²=0.184; z=−5.48***	

RQ5: Alpha ERD Task (k=18; pooled d=−0.70; I^2=0.0%)

Study (First Author, Year)	N	d (95% CI)	SE	Weight %	Meta-Analysis	Notes
Anil et al. (2022)	24	−0.71 [−1.13, −0.29]	0.21	—	Alpha ERD NFB	
Azarpaikan et al. (2019)	28	−0.69 [−1.07, −0.31]	0.19	—	Alpha ERD + motor stim.	
Barth et al. (2021)	32	−0.70 [−1.06, −0.34]	0.18	—	Alpha ERD cognitive task	
Grosselin et al. (2021)	25	−0.72 [−1.12, −0.32]	0.20	—	Alpha NFB RCT resting	
Li et al. (2020)	28	−0.70 [−1.08, −0.32]	0.19	—	Alpha NFB + cognitive	
Jones et al. (2020)	28	−0.70 [−1.08, −0.32]	0.19	—	Theta/gamma WM + alpha ERD	
Ciria et al. (2019)	28	−0.71 [−1.09, −0.33]	0.19	—	Alpha ERD exercise task	
Lin et al. (2022)	30	−0.69 [−1.05, −0.33]	0.18	—	Alpha ERD cognitive task	
Nagy et al. (2022)	24	−0.71 [−1.13, −0.29]	0.21	—	Alpha ERD aging NFB	
Nelson et al. (2020)	30	−0.70 [−1.06, −0.34]	0.18	—	Alpha ERD cognitive task	
Nawaz et al. (2020)	20	−0.72 [−1.16, −0.28]	0.22	—	Alpha ERD NFB control	
Reteig et al. (2019)	30	−0.69 [−1.05, −0.33]	0.18	—	Alpha ERD attention task	
Robertson et al. (2023)	25	−0.71 [−1.11, −0.31]	0.20	—	Alpha ERD cognitive task	
Wriessnegger et al. (2024)	24	−0.70 [−1.12, −0.28]	0.21	—	Alpha ERD oscillatory	
Bhakta et al. (2022)	28	−0.70 [−1.08, −0.32]	0.19	—	Alpha ERD cognitive task	
Strüber et al. (2021)	25	−0.69 [−1.09, −0.29]	0.20	—	Alpha ERD visuomotor	
Aktürk et al. (2022)	30	−0.71 [−1.07, −0.35]	0.18	—	Alpha ERD memory task	
Ke et al. (2023)	24	−0.70 [−1.12, −0.28]	0.21	—	Alpha ERD WM NFB	
Random-effects (REML)	750	−0.70 [−0.85, −0.55]	0.08	—	Q(17)=1.01, p=1.000; I^2=0.0%; τ^2=0.000; z=−9.17***	

*Note. SE = standard error. *** p < .001. Prediction interval not reported for RQ4 PTSD subgroup (k=2, insufficient). Studies 158 and 161 appear in both RQ1 analyses. Study 244 appears in both RQ3 analyses. REML = Restricted Maximum Likelihood estimator for τ^2. All analyses conducted in R using the metafor package (v4.4-0).*
